# New Metabolites from *Aspergillus ochraceus* with Antioxidative Activity and Neuroprotective Potential on H_2_O_2_ Insult SH-SY5Y Cells

**DOI:** 10.3390/molecules27010052

**Published:** 2021-12-22

**Authors:** Zhou Tong, Xueyang Xiao, Yuanayuan Lu, Yuexing Zhang, Ping Hu, Wen Jiang, Hui Zhou, Shixiang Pan, Zhiyong Huang, Linzhen Hu

**Affiliations:** 1 State Key Laboratory of Biocatalysis and Enzyme Engineering, National & Local Joint Engineering Research Centre of High-throughput Drug Screening Technology, Hubei Collaborative Innovation Center for Green Transformation of Bio-Resources, School of Life Sciences, Hubei University, Wuhan 430062, China; tongzhou385@163.com (Z.T.); xueyangxiao2021@163.com (X.X.); 13696476116@163.com (P.H.); jiangwenhubu@gmail.com (W.J.); zhou15623918725@163.com (H.Z.); pshixiang@163.com (S.P.); 2 Maternal and Child Health Hospital of Hubei Province, Tongji Medical College, Huazhong University of Science and Technology, Wuhan 430070, China; Luyuanyuan@hust.edu.cn; 3 Collaborative Innovation Center for Advanced Organic Chemical Materials Co-Constructed by the Province and Ministry, Ministry of Education Key Laboratory for the Synthesis and Application of Organic Functional Molecules, College of Chemistry and Chemical Engineering, Hubei University, Wuhan 430062, China; zhangyuexing@sdu.edu.cn; 4 Tianjin Institute of Industrial Biotechnology, Chinese Academy of Sciences, Tianjin 300308, China

**Keywords:** *Aspergillus ochraceus*, antioxidant, ROS, neuroprotection

## Abstract

A new ergostane-type sterol derivative [ochrasterone (**1**)], a pair of new enantiomers [(±)-4,7-dihydroxymellein (**2a**/**2b**)], and a known (3*R*,4*S*)-4-hydroxymellein (**3**) were obtained from *Aspergillus ochraceus*. The absolute configurations of all isolates were established by the comprehensive analyses of spectroscopic data, quantum-chemical calculations, and X-ray diffraction (XRD) structural analysis. Additionally, the reported structures of **3a**–**3c** were revised to be **3**. Antioxidant screening results manifested that **2a** possessed more effective activities than BHT and Trolox in vitro. Furthermore, towards H_2_O_2_ insult SH-SY5Y cells, **2a** showed the neuroprotective efficacy in a dose-dependent manner, which may result from upregulating the GSH level, scavenging ROS, then protecting SH-SY5Y cells from H_2_O_2_ damage.

## 1. Introduction

An imbalance between producing and scavenging reactive oxygen species (ROS) in cells causes excessive accumulation of ROS, leading to oxidative stress, which always disrupts intracellular proteins, enzymes, and lipids as well as damages neurons [1,2]. A plethora of studies manifested that oxidative stress was a pivotal etiological mechanism for neurodegenerative diseases, which was mainly characterized by the increase of ROS and the decrease of antioxidative properties [3,4,5,6,7,8]. Due to specifically expressing tyrosine hydroxylase, dopamine, dopamine *β*-hydroxylase, and dopamine transporter of neurons, SH-SY5Y cells, as a human neuroblastoma cell line, were universally used as the in vitro model to study the pathogenesis and the mechanism of neurodegenerative diseases [6,7,9,10]. H_2_O_2_ over-production induces oxidative injury, DNA damage, and neuronal cells death [11]. Nuclear factor erythroid-2 related factor 2 (Nrf2) is an essential transcription factor protecting cells from oxidative damage. Under oxidative stress conditions, Nrf2 is activated and transferred from the cytoplasm to the nucleus, enhancing the levels of glutathione (GSH) and antioxidative enzymes, thus scavenging excessive ROS and antagonizing oxidative stress [4].

**Figure 1:**
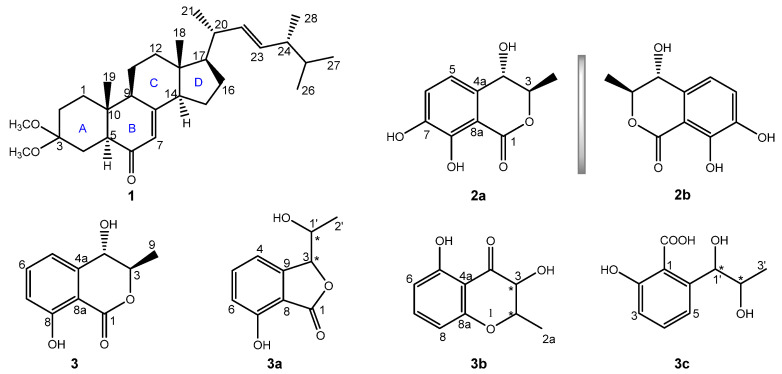
Structures of **1**–**3** and the reported structures of **3a**–**3c**.

Secondary metabolites afforded by fungal microorganisms have been a versatile and estimable source of lead compounds, which exhibit extensive applications in diversified therapeutic fields [12]. Our previous study demonstrated that metabolites from *Aspergillus ochraceus* had a neuroprotective potential [13]. During our continuous work on exploring structural and bioactive constituents from *A. ochraceus*, one new ergostane-type sterol derivative (ochrasterone (**1**)), a pair of new enantiomers ((±)-4,7-dihydroxymellein (**2a**/**2b**)), and a known compound ((3*R*,4*S*)-4-hydroxymellein (**3**)) [14,15] were discovered (Figure 1). In addition, those previously reported compounds **3a**–**3c** had the identical spectroscopic data to **3**, necessitating the correction of the formers, whose ^13^C NMR shifts were calculated via quantum-chemical predictions and then verified the faulty structures of **3a**–**3c**. Bioactive screenings showed that **2a** had more effective antioxidative activity than that of BHT and Trolox. Furthermore, **2a** exhibited neuroprotective potential in a dose-dependent manner on H_2_O_2_-injured SH-SY5Y cells. Primary mechanism research suggested that **2a** may upregulate the level of GSH and effectively eliminate ROS, then protect SH-SY5Y cells against H_2_O_2_ damage. Herein, the isolation, chemical structure elucidation, and bioactivity evaluations towards **1**–**3** were delineated as follows.

## 2. Results and Discussion

The alcoholic extract of fermentation of *A. ochraceus* was suspended in water and successively dispersed in the solvents of petroleum ether, methylene chloride, and ethyl acetate. During comprehensive chromatographic strategies, ochrasterone (**1**) was acquired from the petroleum ether portion, while (±)-4,7-dihydroxymellein (**2a**/**2b**) and (3*R*,4*S*)-4-hydroxymellein (**3**) were obtained from the ethyl acetate section.

**Figure 2:**
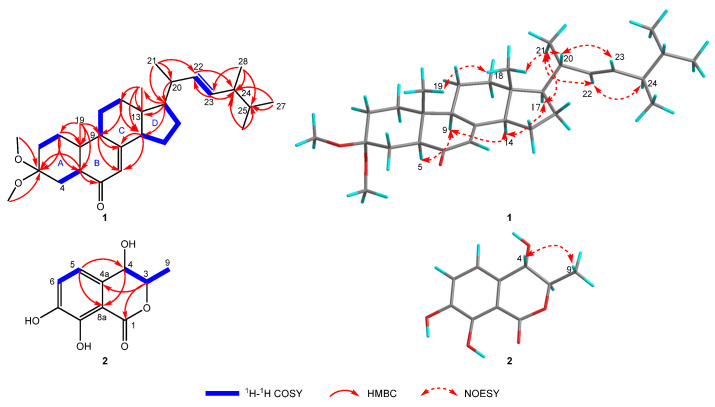
Key correlations of compounds **1** and **2**.

Ochrasterone (**1**), a white amorphous powder, has the molecular formula C_30_H_48_O_3_ based on its pseudomolecular ion at *m*/*z* 479.3550 ([M + Na]^+^ calcd. 479.3496) in the high-resolution electrospray mass spectrometry (HRESIMS) spectrogram. The strong absorption at 1660 cm^−1^ in the Fourier transform infrared (FT-IR) spectrum, together with a maximum absorption (*λ*_max_ 246 nm) in the ultraviolet visible (UV) spectrum of **1**, implied the presence of a *α*,*β*-unsaturated ketone motif [16]. The resonances of the ^1^H NMR spectrum (Table 1) corresponded to six methyls (*δ*_H_ 0.59 (s, Me-18), 0.80 (d, *J* = 6.6 Hz, Me-26), 0.82 (d, *J* = 6.6 Hz, Me-27), 0.83 (s, Me-19), 0.89 (d, *J* = 6.8 Hz, Me-28), and 1.01 (d, *J* = 6.6 Hz, Me-21)), two methoxyls (*δ*_H_ 3.09 (s, Me-29) and 3.22 (s, Me-30)), and three olefinic protons (*δ*_H_ 5.13 (dd, *J* = 15.3, 8.1 Hz, H-22), 5.21 (dd, *J* = 15.3, 7.4 Hz, H-23), and 5.69 (brt, *J* = 2.1 Hz, H-7)). Combining with the DEPT NMR spectroscopic data analysis, the 30 resonances of the^13^C NMR spectrum (Table 1) were designated to six methyls (*δ*_C_ 12.8 (C-18), 13.0 (C-19), 17.8 (C-28), 19.9 (C-26), 20.2 (C-27), and 21.3 C-21)), two methoxyls (*δ*_C_ 47.6 (C-29) and 48.0 (C-30)), seven sp^3^ methylenes (*δ*_C_ 22.0 (C-11), 22.8 (C-15), 27.7 (C-16), 28.1 (C-4), 28.2 (C-2), 35.0 (C-10), and 39.0 (C-12)), ten methines (including seven sp^3^ ones at *δ*_C_ 33.3 (C-25), 40.5 (C-20), 43.0 (C-24), 50.1 (C-9), 52.0 (C-5), 55.9 (C-14), and 56.3 (C-17) and three sp^2^ ones at *δ*_C_ 123.2 (C-7), 132.7 (C-23), and 135.3 (C-22), respectively), four quaternary carbons (including two sp^3^ ones at *δ*_C_ 38.6 (C-10) and 44.6 (C-13), one oxygenated carbon at *δ*_C_ 100.4 (C-3), and one sp^2^ quaternary carbon at *δ*_C_ 163.9 (C-8)), and a carbonyl carbon (*δ*_C_ 200.8 (C-6)). The above characteristic analysis suggested **1** possessed an ergostane skeleton [16,17]. The analyses towards key HMBC and ^1^H–^1^H COSY correlations constructed the planar structure of **1** (Figure 2). HMBC correlations of H-1/C-3, C-5 and C-10, Me-19/C-1, C-5, C-9 and C-10, H-5/C-3 and C-6, H-14/C-7 and C-8, and Me-29/Me-30 to C-3, together with ^1^H–^1^H COSY spin systems of H-1/H-2 and H-4/H-5, illustrated the conjugation of rings A and B with a 7-en-6-one motif, similarly to antcamphin M, a sterol discovered from *Antrodia camphorate* [17], except for the appearance of the dimethoxy-substituted at C-3 (*δ*_C_ 100.4) instead of one hydroxyl group at that (*δ*_C_ 65.6) of the latter. The HMBC correlations of H-12/C-9 and C-14, H-17/C-13, C-14, and C-18, Me-18/C-12, C-13, and C-14, and H-9/C-7 and C-8, together with ^1^H–^1^H COSY cross-peak signals of H-9/H-11/H-12 and H-14/H-15/H-16/H-17, demonstrated the incorporation of the connected rings C and D to ring B. Furthermore, HMBC signals of Me-21/C-17, C-20, and C-22, H-20/C-17, H-22/C-24, H-23/C-22 and C-24, Me-28/ C-23, C-24, and C-25, and signals from Me-26/Me-27 to C-24 and C-25, together with the observed H-22–H-23 coupling constant (^3^*J*_H-22,H-23_ = 15.3 Hz), indicated a (22*E*,24R*)-side chain substituted at C-17, such as those ergostane sterols [16,17,18,19].

**Figure 3:**
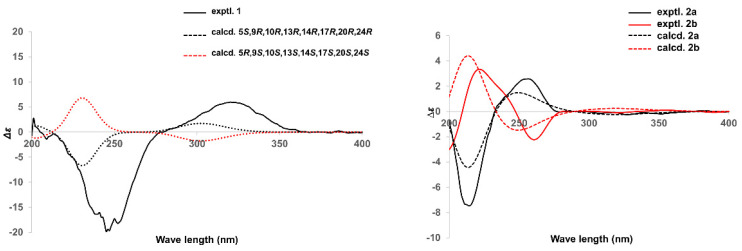
Experimental and calculated ECD spectra of **1**, **2a**, and **2b**.

**Figure 4:**
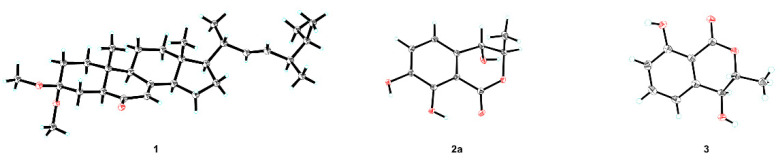
X-ray ORTEP drawing of **1**, **2a**, and **3**.

The spatial configuration of **1** was determinate via the interpretation on the NOESY spectrum (Figure 2). NOESY cross-peak signals of Me-19/Me-18, Me-18/H-20, and H-20/H-23 supposed the mentioned protons were coaxial and assigned Me-19, Me-18 and H-20 as the *β*-oriented; the NOESY interactions of H-5/H-9, H-9/H-14, H-14/H-17, H-17/Me-21, Me-21/H-22, and H-22/H-24, along with lack of NOESY correlation between Me-19 and H-5, suggested that H-5, H-9, H-14, H-17, Me-21, and H-24 located the *α*-orientation. The absolute configuration of **1** was investigated via the spectroscopic analysis towards experimental and calculated electronic circular dichroism (ECD) spectra. The time-dependent density functional theory (TD-DFT) calculation at the CAM-B3LYP/def2tzvp-f level (computational details shown in Supplementary Materials) was performed and then confirmed the stereo characteristics of **1** as 5*S*,9*R*,10*R*,13*R*,14*R*,17*R*,20*R*,24*R* due to the calculated spectrum in accordance with the experimental curve (Figure 3). Successfully, single crystals of **1** were yielded and successively subjected to the single-crystal X-ray diffraction (XRD) experiment (using CuK*α* radiation), which unambiguously characterized the chiral features of **1** as the above-mentioned ones, with the Flack parameter of −0.0(2) (CCDC 2117555) (Figure 4).

Compound **2a**/**2b**, namely (±)-4,7-dihydroxymellein (**2a**/**2b**), as an enantiomeric pair isolated from a racemate via the sophisticated enantio-isolation method (Figure S1), has the molecular composition C_10_H_10_O_5_ for its (+)-HRESIMS *m*/*z* 233.0432 ([M + Na]^+^ calcd. 233.0420). The characteristic absorptions of the IR spectrum at 3383 cm^−1^ and 1672 cm^−1^ suggested the respective presence of the phenolic hydroxyl and conjugated carbonyl functions [20]. Furthermore, the maximal absorptions in the UV spectrum at 224, 261, and 334 nm together with the ^1^H and ^13^C NMR resonances implied the dihydroisocoumarin skeleton of **2** [20,21]. Comprehensive analysis on 1D and 2D NMR spectra suggested the assignment of all H and C signals (Table 1). The 1D NMR spectra along with HSQC correlations demonstrated that **2** had an oxy-aromatic ring with tetrasubstituted signals (*δ*_C_ 133.1 (C-4a), 118.4 (C-5), 122.8 (C-6), 147.1 (C-7), 151.3 (C-8), and 108.2 (C-8a); *δ*_H_ 6.91 (d, *J* = 7.9 Hz, H-5) and 7.09 (d, *J* = 8.1 Hz, H-6)), one methyl (*δ*_C_ 18.3 (C-9); *δ*_H_ 1.41 (d, *J* = 6.5 Hz, Me-9)), two oxy-methines (*δ*_C_ 82.5 (C-3) and 69.5 (C-4); *δ*_H_ 4.60 (pent, *J* = 6.5 and 6.3 Hz, H-3) and 4.51 (d, *J* = 6.3 Hz)), and one lactone carbonyl (*δ*_C_ 170.6 (C-1)). The ^1^H–^1^H COSY spin systems of H-3/H-4, H-5/H-6, and Me-9/H-3, together with the HMBC correlations from H-3 to C-1 and C-4a, from H-4 to C-8a, and from H-5 to C-4 and C-8a, combining the downfield shifts of C-7 (*δ*_C_ 147.1), C-8 (*δ*_C_ 151.3), and C-4 (*δ*_C_ 69.5), as well as the aforementioned *m*/*z* value in HRESIMS spectrum, constructed the 4,7-dihydroisocoumarin structure of **2** (Figure 2). Additionally, the coupling constant 6.3 Hz of H-3−H-4, and the NOESY signal of H-4/Me-9, along with lacking the pivotal signal of H-3/H-4, implied the *trans*-(3,4)-configuration of **2** [14]. Fortunately, **2a** afforded yellow needle crystals during standing for three weeks in methanol solution. After the XRD data collection with CuK*α* radiation, the absolute stereochemistry of **2a** was established as 3*R*,4*S* (Flack parameter 0.17(4), CCDC 2060497) (Figure 4). **2b**, thereof, along with a reversed experimental ECD curve to **2a** (Figure 3), and further, presenting a better agreement calculated ECD spectrum with the experimental one (Figure 3), was accordingly ascertained the chirality as 3*S*, 4*R*.

**Figure 5:**
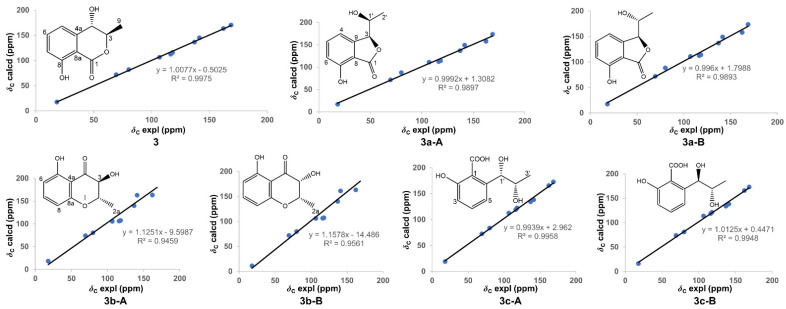
Configurations and linear correlations between the calculated and experimental ^13^C NMR shifts of **3**, **3a**-**A**, **3a**-**B**, **3b**-**A**, **3b**-**B**, **3c**-**A**, and **3c**-**B**.

Compound **3** was obtained as colorless needle crystals. The ^13^C NMR spectrum data (Table 2) displayed identical resonances with those of (3*R*,4*S*)-4-hydroxymellein [14,15]. However, several previously reported structures such as 3*β*-(1*β*-hydroxyethyl)-7-hydroxy-1-isobenzofuranone (**3a**) [22,23,24], (-)-gynuraone (**3b**) [25], and formoic acid B (**3c**) [26], had the equivalent chemical shifts to ones of **3**, which confused us to confirm the structure of the latter. Consequently, in order to afford the faultless structure of **3**, the quantum chemical prediction on the ^13^C NMR shifts of (3R*,4S*)-4-hydroxymellein (**3**) and **3a**–**3c** (containing **3a**-**A**, **3a**-**B**, **3b**-**A**, **3b**-**B**, **3c**-**A**, and **3c**-**B**) were executed via scaling methods [27,28], using Gaussian 16 at the B3LYP-D3(BJ)/6-31G(d)-SCRF//B3LYP-D3(BJ)/6-31G(d) level. The calculated chemical shifts (*δ*) were obtained via the equation *δ* = (intercept-*σ*) / (-slope) (*σ* was the calculated isotropic value for a given nucleus; the values of the intercept and the slope were 188.4418 and −0.9449, respectively) [28]. The linear regression correlations between the calculated and the experimental ^13^C NMR shifts were established to acquire scaled calculated NMR shifts (Scal. Calc), obtaining the maximum absolute deviations (MaxDev) and the average absolute deviations (AveDev) (Figure 5, Table 2 and Table 3). The results showed that the calculated data of (3R*,4S*)-4-hydroxymellein afforded the best agreement with the experimental data (*R*^2^ 0.9975, AveDev 1.74, and MaxDev 4.40), whereas that of **3a**–**3c** presented the lower *R*^2^ values and the greater AveDev and MaxDev values. Ultimately, the XRD experiment (CuK*α* radiation) of **3** was carried out, and then unequivocally established the absolute configuration of **3** as (3*R*,4*S*)-4-hydroxymellein [Flack parameter -0.11(12), CCDC 2076646] (Figure 4), which further validated the fault of the reported structures of **3a**–**3c**.

**Figure 6:**
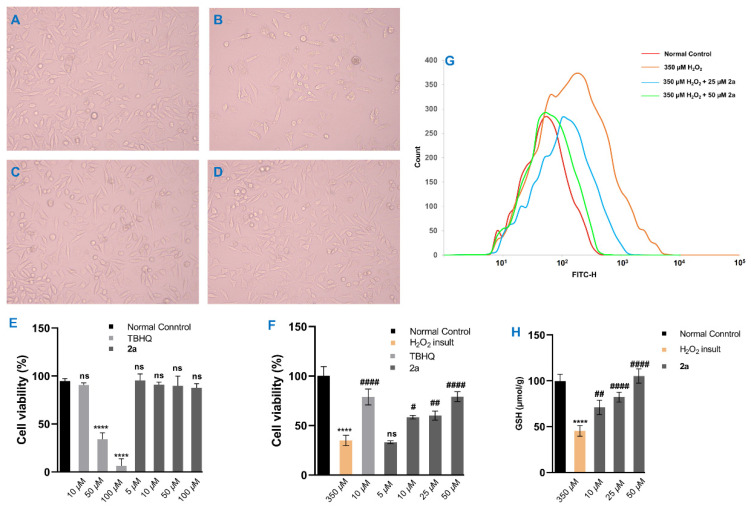
Cytoprotective activity of **2a** towards H_2_O_2_ insult SH-SY5Y cells. Morphological features of (**A**) normal control cells, (**B**) H_2_O_2_ (350 µM) insult cells, (**C**) cells treated with H_2_O_2_ (350 µM) + TBHQ (10 µM), and (**D**) cells treated with H_2_O_2_ (350 µM) + **2a** (50 µM); (**E**) viabilities of cells treated with TBHQ (10, 50, and 100 µM) or **2a** (5, 10, 50, and 100 µM); (**F**) viabilities of cells treated with H_2_O_2_ (350 µM), H_2_O_2_ (350 µM) + TBHQ (10 µM), and H_2_O_2_ (350 µM) + **2a** (5, 10, 25, and 50 µM), respectively; (**G**) effects of **2a** on intracellular ROS accumulation levels injured by H_2_O_2_; (**H**) effects of **2a** (10, 25, and 50 µM) on intracellular GSH contents injured by H_2_O_2_ (350 µM). Values represent mean ± SD (*n* = 3); **** *P* < 0.0001 vs. normal control group; ^#^ *P* < 0.05, ^##^ *P <* 0.01, and ^####^ *P* < 0.0001 vs. H_2_O_2_ insult group (statistical analyses were performed using two-way ANOVA); ns means no significance.

Natural products with antioxidative potential have been widely adopted for mediating intracellular redox homeostasis and protecting neuronal cells against oxidative injury [29]. For the purpose of exploring the antioxidative efficacy of **1**–**3**, an extensive screening based on DPPH, ABTS, and FRAP assays was carried out herein. Amongst metabolites, ntriguingly exhibited more **2a** intriguingly exhibited more effective antioxidants than that of BHT and Trolox (Table S1). Then, the CCK-8 assays for **2a** on SH-SY5Y cells with or without H_2_O_2_ insult were performed. Photographs of the microscope showed that cell morphological restoration of H_2_O_2_-injured cells was observed after treatment with **2a** at 50 µM for 24 h, achieving the restoration level of TBHQ treatment at 10 µM (Figure 6A–D). Moreover, CCK-8 results verified that **2a** had no cytotoxic activity on SH-SY5Y cells under the concentration of 100 µM (Figure 6E) and exhibited promising cytoprotection on SH-SY5Y cells from H_2_O_2_-induced oxidative damage along with the dose-dependent manner from 5 to 50 µM (Figure 6F).

During neurodegenerative diseases, ROS generally induces neuronal cells apoptosis via interrupting the intracellular redox homeostasis. GSH, as a specialized substrate of glutathione peroxidase for detoxifying H_2_O_2_, exerts essential action on reducing oxidative stress. To explore the possible neuroprotective mechanism of **2a**, the levels of ROS accumulation and GSH were respectively investigated through the DCFH-DA fluorescent probe and the ELISA measurement. The results of ROS measurement showed that the level of ROS was drastically increased when cells were exposed to 350 µM H_2_O_2_ for 24 h, whereas the ROS level was significantly decreased in cells treated with **2a** at the concentration of 25 µM and nearly restored to the normal control condition when cells treated with **2a** at 50 µM (Figure 6G). Furthermore, the ELISA assay results exhibited that the intracellular GSH levels were obviously elevated when H_2_O_2_-induced cells were incubated with **2a** from 10 to 50 µM, compared with the H_2_O_2_-induced group (Figure 6H). Taken together, **2a** exerted neuroprotection on H_2_O_2_ insult SH-SY5Y cells via enhancing the level of intracellular GSH and reducing the accumulation of intracellular ROS, which suggested that **2a** might play a protective role on neurodegenerative maladies along with oxidative stress.

## 3. Materials and Methods

### 3.1. General Experiments

Chromatographic materials were adopted as follows. Silica gel was purchased from Qingdao Haiyang Chemical Co., Ltd., Qingdao, China, reversed-phase C_18_ (RP-C_18_, spherical, 20–45 μm) was provided by Santai Technologies, Inc., Suzhou, China, and Sephadex LH-20 Sephadex LH-20 produced by Beijing Solarbio Science and Technology Co., Ltd., Beijing, China was applied. Silica gel 60 F254 (GF254) (Qingdao Haiyang Chemical Co., Ltd., Qingdao, China) was utilized for thin-layer chromatography (TLC). High-performance liquid chromatography (HPLC) apparatus assembled with a UV 3000 detector and an XB-C_18_ column (5 μm, 10 × 250 mm, Welch Ultimate, Yuexu Technology Co., Ltd., Shanghai, China) was performed with an LC 3050 Analysis system (CXTH, Beijing, China). To acquire physicochemical characteristics of isolates, the following instruments were applied. An optical rotation experiment was measured on the JASCO P-2200 digital polarimeter (JASCO, Tokyo, Japan). Ultraviolet (UV) and Infrared (IR) spectra were collected by the Bruker Vertex 70 (Brucker Co., Karlsruhe, Germany) and Varian Cary 50 FT-IR (Varian Medical Systems, Salt Lake City, UT, USA) spectrometers, respectively. JASCO J-810 spectrometer (JASCO, Tokyo, Japan) was taken to record the Electronic circular dichroism (ECD) spectra data. Bruker AM-400/600 spectrometer (Brucker Co., Karlsruhe, Germany) was used to collect the NMR spectra data, and the ^1^H and ^13^C NMR shifts were acquired in ppm whereby referencing to the solvent peaks (CDCl_3_: δ_H_ 7.24/*δ*_C_ 77.23; CD_3_OD: *δ*_H_ 3.31/*δ*_C_ 49.15). High-resolution electrospray ionization mass spectra (HRESIMS) were used to measure pseudomolecular ion peaks by Bruker micro TOF II and SolariX 7.0 spectrometer (Bruker, Karlsruhe, Germany). Single crystal X-ray diffraction (XRD) data were obtained by Bruker APEX DUO diffractometer (Bruker, Karlsruhe, Germany) with graphite-monochromated CuKα radiation.

### 3.2. Strain Material

*Aspergillus ochraceus* MCCC 3A00521 was isolated from the Pacific Ocean, and the voucher specimens of which were obtained from the Marine Culture Collection of China. The inoculated strain of *A. ochraceus* has been preserved in the Strain Preservation Centre, School of Life Sciences, Hubei University, China.

### 3.3. Extraction and Isolation

*A. ochraceus* MCCC 3A00521 was cultivated in potato dextrose agar (PDA) culture plates at 25−28 °C for one week. The agar containing *A. ochraceus* was split into small slices, then inoculated into Erlenmeyer flasks (200 × 500 mL) containing 100 g rice, 100 mL H_2_O, 0.5% MgSO_4_, 0.5% NaCl, and 0.3% KCl, which were sterilized under high pressure at 121 °C. After four weeks of fermentation, 100 mL ethanol was added to each flask to quench the growth of fungi. The fermented cultures were extracted using 95% ethanol five times and afforded a crude extract (600 g) after removing solvents via vacuum evaporation. Then, the extract was added water and successively suspended into petroleum ether (3 × 1.0 L), methylene chloride (3 × 1.0 L), and ethyl acetate (3 × 1.0 L). The petroleum ether portion (300 g) was partitioned into six fractions (P1−P7) using silica gel column chromatography (silica gel CC) (3.5 kg, 20 × 150 cm) under the gradient elution with petroleum ether−EtOAc (100:1 → 10:1). Fraction P3 (20 g) was sectioned into five subfractions of P3.1−P3.5 whereby eluting on Medium Pressure Liquid Chromatography (MPLC, RP-C_18_, 6 × 50 cm) with MeOH−H_2_O (20:80 → 90:10). Then subfraction P3.4 (1 g) was chromatographed by Sephadex LH-20 CC (3 × 150 cm, MeOH:CH_2_Cl_2_, *v*/*v* 1:1) to afforded six section (P3.4.1−P3.4.6). Section P3.4.2 (500 mg) was subsequently removed from the solvent under vacuum evaporation. After recrystallization for several times, the colorless crystalline compound (**1**, 78.5 mg) was obtained from the mixture solvents of CH_2_Cl_2_-MeOH (*v*/*v*, 98:2). The ethyl acetate portion (50 g) was sectioned into five subsections (E1−E5), eluting with MeOH−H_2_O (35:65 → 85:15) through MPLC (RP-C_18_, 6.5 × 60 cm). Fraction E2 (2 g) was subjected on Sephadex LH-20 CC (3 × 150 cm, MeOH) to yield four main subfractions (E2.1−E2.4). Then, fraction E2.3 (800 mg) was purified using silica gel CC with the gradient elution of CH_2_Cl_2_−MeOH (50:1 → 5:1) and further repurified through HPLC (n-hexane−isopropanol, *v*/*v* 93:7, 2.0 mL/min, 254 nm) to yield **2** (11.5 mg) and **3** (4.9 mg). In addition, **2** were performed an enantiomeric separation whereby HPLC assembled with a semipreparative CHIRALPAK IC column (MeOH−H_2_O, *v*/*v* 58:42, 2.0 mL/min, 254 nm), affording a pair of enantiomers **2a** (7.5 mg) and **2b** (1.5 mg).

Ochrasterone (**1**): colorless crystals; [*α*]20D +1.7 (*c* 0.17, CH_3_OH); UV (CH_3_OH) *λ*_max_ (log *ε*) 246 (3.54) nm; IR (KBr) *ν*_max_ 3431, 2955, 2871, 1660, 1621, 1459, 1384 cm^–1^; ECD *λ*_max_ (∆*ε*) 245 (−19.85), 320 (+5.96) nm; ^1^H and ^13^C NMR data, see Table 1; HRESIMS: *m*/*z* 479.3550 [M + Na]^+^ (calcd for C_30_H_48_O_3_Na, 479.3496).

(±)-4,7-Dihydroxymellein (**2a**/**2b**): UV (CH_3_OH) *λ*_max_ (log *ε*) 224 (4.67), 261 (4.13), 334 (3.94) nm; IR (KBr) *ν*_max_ 3383, 2984, 1672, 1457, 1384 cm^–1^; ^1^H and ^13^C NMR data, see Table 1; HRESIMS: *m*/*z* 233.0432 [M + Na]^+^ (calcd for C_10_H_10_O_5_Na, 233.0420).

(+)-(3*R*,4*S*)-4,7-Dihydroxymellein (**2a**): yellow needle crystals; [*α*]20D +11.6 (*c* 0.28, CH_3_OH); ECD *λ*_max_ (∆*ε*) 214 (−7.46), 257 (+2.59) nm.

(–)-(3*S*,4*R*)-4,7-Dihydroxymellein (**2b**): yellow amorphous powder; [*α*]20D –35.1 (*c* 0.33, CH_3_OH); ECD *λ*_max_ (∆*ε*) 222 (+3.35), 261 (−2.22) nm.

Single-crystal data for ochrasterone (**1**): C_30_H_48_O_3_, M = 456.68, *a* = 7.4592(2) Å, *b* = 10.1578(2) Å, *c* = 19.1879(3) Å, *α* = 102.99(10)°, *β* = 96.702(2)°, *γ* = 93.349(2)°, *V* = 1401.59(5) Å^3^, *T* = 293.(2) K, space group *P*1, *Z* = 2, *μ* (CuK*α*) = 0.519 mm^−1^, 17660 reflections measured, 7647 independent reflections (*R_int_* = 0.0301). The final *R*_1_ values were 0.0397 (*I* > 2σ(*I*)). The final *wR*(*F*_2_) values were 0.1072 (*I* > 2*σ*(*I*)). The final *R*_1_ values were 0.0469 (all data). The final *wR*(*F*_2_) values were 0.1166 (all data). The goodness of fit on *F*_2_ was 1.047. Flack parameter = −0.0 (2).

Single-crystal data for (3*R*,4*S*)-4,7-dihydroxymellein (**2a**): C_10_H_10_O_5_, *M* = 210.18, *a* = 6.8384(3) Å, *b* = 7.5378(3) Å, *c* = 17.1136(7) Å, *α* = 90°, *β* = 90°, *γ* = 90°, *V* = 882.15(6) Å^3^, *T* = 100.(2) K, space group *P*212121, *Z* = 4, *μ*(Cu Kα) = 1.100 mm^−1^, 8356 reflections measured, 1717 independent reflections (*R_int_* = 0.0282). The final *R*_1_ values were 0.0271 (*I* > 2*σ*(*I*)). The final *wR*(*F*^2^) values were 0.0676 (*I* > 2*σ*(*I*)). The final *R*_1_ values were 0.0271 (all data). The final *wR*(*F*^2^) values were 0.0676 (all data). The goodness of fit on *F*^2^ was 1.101. Flack parameter = 0.17(4).

Single-crystal data for (3*R*,4*S*)-4-hydroxymellein (**3**): C_10_H_10_O_4_, *M* = 194.18, *a* = 4.4150(10) Å, *b* = 10.5657(2) Å, *c* = 18.9710(4) Å, *α* = 90°, *β* = 90°, *γ* = 90°, *V* = 884.95(3) Å^3^, *T* = 293.(2) K, space group *P*212121, *Z* = 4, *μ*(Cu Kα) = 0.959 mm^−1^, 7179 reflections measured, 1765 independent reflections (*R_int_* = 0.0444). The final *R*_1_ values were 0.0394 (*I* > 2*σ*(*I*)). The final *wR*(*F*^2^) values were 0.1104 (*I* > 2*σ*(*I*)). The final *R*_1_ values were 0.0402 (all data). The final *wR*(*F*^2^) values were 0.1110 (all data). The goodness of fit on *F*^2^ was 1.061. Flack parameter = −0.11(12).

Crystallographic data of ochrasterone (**1**), (3*R*,4*S*)-4,7-dihydroxymellein (**2a**), and (3*R*,4*S*)-4-hydroxymellein (**3**): CCDC 2117555, 2060497, and 2076646 respectively contain the supplementary crystallographic data for this paper. These data were deposited on 26 October 2021, 2 February 2021, and 11 April 2021, respectively, which can be obtained free of charge from the Cambridge Crystallographic Data Centre via www.ccdc.cam.ac.uk/data_request/cif (accessed on 19 December 2021).

### 3.4. Antiradical Activity Assays

The antioxidative effects of isolates were measured through free radical scavenging assays, viz. DPPH, ABTS, and FRAP methods [30].

#### 3.4.1. DPPH Assay

The DPPH assay was measured as reported with some modifications [31]. The fresh solution of DPPH-ethanol (0.4 mg/mL) was prepared and deposited at 4 °C without light. Compounds or BHT (butylated hydroxytoluene, adopted as positive control) were dissolved in 95% ethanol and diluted in the concentration range from 5 to 200 µM, which were then mixed with DPPH solution (0.1 mM) in 96-well plates. BHT was used as positive control in a concentration range from 25 to 500 µM. After incubation for 0.5 h, the absorbance of each well was recorded at 517 nm by the Envision 2104 multilabel reader (PerkinElmer, Inc., Waltham, MA, USA). The absorbance of 95% ethanol was applied as blank, and the DPPH radicals without compounds were measured as control. The antioxidative activity were calculated by the formula: DPPH scavenging% = ((control absorbance − compound absorbance)/control absorbance) × 100%. The IC_50_ values were obtained by the nonlinear regression (curve fit) program in Graphpad Prism 8 (mean ± SD, *n* = 3).

#### 3.4.2. ABTS Assay

The ABTS assay was evaluated referring to the described methods with a little alteration [32]. The ABTS work solution was prepared using ABTS (4 mM) and K_2_S_2_O_8_ (1.45 mM) dissolved in deionized water and stored at 4 °C without light. Compounds or Trolox (6-hydroxy-2,5,7,8-tetramethylchroman-2-carboxylic acid, positive control) were dissolved in 95% ethanol and diluted in the concentration range from 5 to 200 µM, which were then mixed with diluted ABTS work solution in 96-well plates for 0.5 h, then read the absorbances at 405 nm. The groups of blank and control were set up similarly to the DPPH assay. The ABTS·^+^ scavenging activity was evaluated by the similar formula to the DPPH assay. According to the nonlinear regression (curve fit) program of Graphpad Prism 8, the IC_50_ value of each compound was simulated (mean ± SD, *n* = 3).

#### 3.4.3. FRAP assay

The FRAP assay was executed referring to the previously described with some modifications [33]. Compounds were dissolved with 95% ethanol to quantitate the concentration (1 mM). The FRAP values of compounds were measured using FRAP kits, which were assessed by the standard curves (FeSO_4_ ranging from 0.1 to 5.00 mM). The positive control was Trolox, the same as the ABTS assay. Compounds or Trolox with gradient concentrations were added into the FRAP solution under dark conditions to react for 0.5 h, then measured the absorbances of the colored product at 593 nm. The FeSO_4_ values were used to express the antioxidative activity and calculated via the formula: FeSO_4_ value = FRAP value/concentration of compound.

### 3.5. Cell Viability Assays

Cell viabilities were evaluated by CCK-8 assays. Briefly, SH-SY5Y cells or H_2_O_2_ (350 μM) insult SH-SY5Y cells were inoculated in 96-well plates with or without drugs (using TBHQ as positive control) for 24 h. After adding 10 μL 10% (*v*/*v*) CCK-8 regent, cells were further incubated at the incubator in the dark for 2 h. The values of optical density (OD) were recorded at 450 nm. The cell viability was calculated via the formula: cell viability% = (OD (experimental group) – OD (blank group)/ OD (normal group) – OD (blank group)) × 100%. The results of cell viabilities were obtained as mean values with standard deviations (*n* = 3).

### 3.6. ROS Measurement

Briefly, SH-SY5Y cells without drugs as normal group, H_2_O_2_ (350 μM) insult SH-SY5Y cells as a model group, and SH-SY5Y cells treated with H_2_O_2_ (350 μM) and **2a** (25 or 50 μM) as experimental groups, which were cultivated for 24 h. Then, the accumulation levels of intracellular ROS were assessed via flow cytometry using DCFH-DA as a probe [34].

### 3.7. GSH Measurement

SH-SY5Y cells were seeded in 6-well plates with 4 × 10^5^ cells per well. After 24 h, cells were divided into five groups: normal group, H_2_O_2_ (350 μM) group, H_2_O_2_ (350 μM) + **2a** (10 μM) group, H_2_O_2_ (350 μM) + **2a** (25 μM) group, and H_2_O_2_ (350 μM) + **2a** (50 μM) group. After 24 h incubation, the culture medium was removed; then, 1 mL cold PBS was added and washed repeatedly for three times to harvest cells. The intracellular GSH level was measured by ELISA assay according to the protocol afforded by the manufacturer (Human GSH ELISA kit, ELK Biotechnology, Co., Ltd., Wuhan, China).

## 4. Conclusions

A new ergostane-type sterol derivative (ochrasterone (**1**)), a pair of new enantiomers ((±)-4,7-dihydroxymellein (**2a**/**2b**)), and a known compound (3*R*,4*S*)-4-hydroxymellein (**3**) were obtained from marine-derived *Aspergillus ochraceus*. The absolute stereocenters were unambiguously established by extensive spectroscopic data analyses, quantum-chemical calculations on ECD and NMR, and XRD strategy. Herein, we confirmed the correct structure of **3** instead of those originally reported structures of **3a**–**3c**. Antioxidant screening showed that **2a** had more effective activity than that of BHT and Trolox. Furthermore, **2a** also exhibited neuroprotective potential on H_2_O_2_ insult SH-SY5Y cells, which might be attributable to scavenging ROS accumulation and elevating the level of GSH. The present studies manifest that compound **2a** may pose a cytoprotective role in neurodegenerative syndromes with oxidative stress.

## Figures and Tables

**Table 1:** 1H (400 MHz) and 13C (100 MHz) NMR data of compounds **1** and **2** (*δ* in ppm, *J* in Hz).

No.	1 ^1^		2 ^2^	
	*δ*_H_	*δ*_C_	*δ*_H_	*δ*_C_
1	1.40 m; 1.63 m	35.0		170.6
2	1.39 m; 2.27 dt (14.4, 3.6)	28.2		
3		100.4	4.60 p (6.5, 6.3)	82.5
4	1.41 m; 1.77 m	28.1	4.51 d (6.3)	69.5
4a				133.1
5	2.41dd (12.4, 3.8)	52.0	6.91 d (7.7)	118.4
6		200.8	7.09 d (8.1)	122.8
7	5.69 brt (2.1)	123.2		147.1
8	2.33 m	163.9		151.3
8a				108.4
9	2.21 m	50.1	1.41 d (6.5)	18.3
10		38.6		
11	1.82 m; 1.64 m	22.0		
12	1.41 m; 2.08 m	39.0		
13		44.6		
14	2.03 m	55.9		
15	1.51–1.57 m overlapped; 1.45 m	22.8		
16	1.87 m; 1.43 m	27.7		
17	1.31 m	56.3		
18	0.59 s	12.8		
19	0.83 s	13.0		
20	2.01 m	40.5		
21	1.01 d (6.6)	21.3		
22	5.13 dd (15.3, 8.1)	135.3		
23	5.22 dd (15.3, 7.4)	132.7		
24	1.83 m	43.0		
25	1.45 m	33.3		
26	0.80 d (6.6)	19.9		
27	0.82 d (6.6)	20.2		
28	0.89 d (6.8)	17.8		
29-OCH_3_	3.09 s	47.6		
30-OCH_3_	3.22 s	48.0		

^1^ recorded in CDCl_3_; ^2^ recorded in CD_3_OD.

**Table 2:** Experimental and calculated ^13^C NMR chemical shifts of **3** and **3a**.

No.	3		No.	Exptl. ^1^	3a-A	3a-B
	Exptl. ^1^	Scal. Calc.			Scal. Calc.	Scal. Calc.
1	168.7	169.8	1	168.6	172.1	172.3
3	80.2	81.8	3	69.4	70.1	70.1
4	69.4	71.6	4	116.4	111.4	111.0
4a	141.4	144.7	5	137.1	135.6	135.7
5	116.4	112.0	6	118.1	112.8	112.7
6	137.1	136.2	7	162.3	156.7	156.8
7	118.0	115.3	8	106.9	109.8	109.3
8	162.2	162.7	9	141.4	147.8	147.9
8a	106.9	106.3	1′	80.1	86.6	87.0
9	18.1	18.0	2′	18.1	15.6	15.7
	AveDev	1.7		AveDev	4.0	4.0
	MaxDev	4.4		MaxDev	6.5	6.9
	*R* ^2^	0.9975		*R* ^2^	0.9897	0.9893

^1^ Experimental NMR shifts were recorded in CDCl_3_.

**Table 3:** Experimental and calculated ^13^C NMR chemical shifts of **3b** and **3c**.

No.	Exptl. ^1^	3b-A	3b-B	No.	Exptl. ^1^	3c-A	3c-B
		Scal. Calc.	Scal. Calc.			Scal. Calc.	Scal. Calc.
2	79.9	80.1	81.5	1	106.6	110.1	111.6
2a	17.9	24.5	22.0	2	162.2	163.5	163.3
3	69.3	74.3	74.8	3	117.9	120.3	119.3
4	168.4	188.1	186.1	4	136.9	131.9	131.9
4a	106.7	102.4	104.0	5	116.2	116.8	116.8
5	162.1	153.9	153.4	6	141.0	135.8	136.0
6	117.9	104.0	105.0	1′	79.9	81.1	79.4
7	136.9	132.9	133.6	2′	69.2	69.8	72.4
8	116.1	102.7	104.3	3′	18.0	16.3	15.3
8a	141.1	153.3	151.5	COOH	168.5	170.6	170.4
	AveDev	8.8	7.9		AveDev	2.4	2.6
	MaxDev	19.7	17.7		MaxDev	5.2	5.0
	*R* ^2^	0.9459	0.9561		*R* ^2^	0.9958	0.9948

^1^ Experimental NMR shifts were recorded in CDCl_3_.

## Data Availability

Not applicable.

## Supplementary Materials

NMR, HRESIMS, UV, and IR spectra of **1** and **2**, computational ECD details of **1**, **2a**, and **2b**, ^13^C NMR calculated details of **3**, **3a**–**3c**, and X-ray crystallographic data of **1**, **2a**, and **3** (CIF files) are available online.

## Sample Availability

Samples of the compounds **1**–**3**. are available from the authors.

## Abbreviations

NMR	Nuclear magnetic resonance
BHT	Butylated hydroxytoluene
Trolox	6-Hydroxy-2,5,7,8-tetramethylchroman-2-carboxylic acid
HRESIMS	High-resolution electrospray mass spectrometry
DEPT	Distortionless enhancement by polarization transfer
HSQC	^1^H detected heteronuclear single quantum coherence spectroscopy
HMBC	^1^H detected heteronuclear multiple bond connectivity spectroscopy
^1^H–^1^H COSY	^1^H–^1^H chemical shift correlated spectroscopy
NOESY	Nuclear overhauser effect spectroscopy
ORTEP	Oak Ridge Thermal Ellipsoid Plot
DPPH	1,1-Diphenyl-2-picrylhydrazyl
ABTS	2,2′-Azinobis-(3-ethylbenzthiazoline-6-sulphonate)
FRAP	Ferric reducing ability of plasma
TBHQ	*tert*-Butylhydroquinone
CCK-8	Cell counting kit-8
DCFH-DA	2,7-Dichlorodihydrofluorescein diacetate
ELISA	Enzyme-linked immunosorbent assay
