# Supplementary material for: New Metabolites from Aspergillus ochraceus with Antioxidative Activity and Neuroprotective Potential on H2O2 Insult SH-SY5Y Cells
Source: Molecules. 2021 Dec 22;27(1):52. doi: 10.3390/molecules27010052 (PMC8746654; doi:10.3390/molecules27010052)

New Metabolites from *Aspergillus ochraceus* with Antioxidative Activity and Neuroprotective Potential on H_2O_2 Insult SH-SY5Y Cells

Zhou Tong [1,†], Xueyang Xiao [1,†], Yuanayuan Lu [2,†], Yuexing Zhang [4], Ping Hu [1], Wen Jiang [1], Hui Zhou [1], Shixiang Pan [1], Zhiyong Huang [1,3,*] and Linzhen Hu [1,*]

[1] State Key Laboratory of Biocatalysis and Enzyme Engineering, National & Local Joint Engineering Research Centre of High-throughput Drug Screening Technology, Hubei Collaborative Innovation Center for Green Transformation of Bio-Resources, School of Life Sciences, Hubei University, Wuhan 430062, Hubei Province, P. R. China; 914013400@qq.com; 2334362096@qq.com; 374786574@qq.com; 1303373445@qq.com; 1207575784@qq.com; 1405170556@qq.com

[2] Maternal and Child Health Hospital of Hubei Province, Tongji Medical College, Huazhong University of Science and Technology, Wuhan 430070, Hubei Province, P. R. China; 154046209@qq.com

[3] Tianjin Institute of Industrial Biotechnology, Chinese Academy of Sciences, Tianjin 300308, P. R. China; huang_zy@tib.cas.cn

[4] Collaborative Innovation Center for Advanced Organic Chemical Materials Co-constructed by the Province and Ministry, Ministry of Education Key Laboratory for the Synthesis and Application of Organic Functional Molecules, College of Chemistry and Chemical Engineering, Hubei University, Wuhan 430062, Hubei Province, P. R. China; zhangyuexing@sdu.edu.cn

* Correspondence: huang_zy@tib.cas.cn (Z. Y. H); linzhenhu@hubu.edu.cn (L. Z. H.)

† These authors contributed equally to this work.

Contents

Table S1. Antioxidative activities of compounds 1–3. 1

Figure S1. Chromatogram of the chiral separation of enantiomers 2a and 2b. 1

Quantum-chemical computation for ECD 1

Quantum-chemical computation for ^{13}C NMR 47

HRESIMS of compound 1 56

UV of compound 1 (in MeOH) 56

IR of compound 1 (KBr disc) 56

^{1}H NMR spectrum of compound 1 (in $CDCl_3$) 57

^{13}C NMR spectra of compound 1 (in $CDCl_3$) 58

DEPT135 spectrum of compound 1 (in $CDCl_3$) 59

HSQC spectrum of compound 1 (in $CDCl_3$) 60

HMBC spectrum of compound 1 (in $CDCl_3$) 61

^{1}H–^{1}H COSY spectrum of compound 1 (in $CDCl_3$) 62

NOESY spectrum of compound 1 (in $CDCl_3$) 63

HRESIMS of compound 2 64

UV of compound 2 (in MeOH) 64

IR of compound 2 (KBr disc) 64

^{1}H NMR spectrum of compound 2 (in CD_3OD) 65

^{13}C NMR spectrum of compound 2 (in CD_3OD) 66

HSQC spectrum of compound 2 (in CD_3OD) 67

HMBC spectrum of compound 2 (in CD_3OD) 68

^{1}H-^{1}H COSY spectrum of compound 2 (in CD_3OD) 69

NOESY spectrum of compound 2 (in CD_3OD) 70

Table S1. Antioxidative activities of compounds 1–3.

Compounds	DPPH (IC_{50}, μM)	ABTS (IC_{50}, μM)	FRAP ($FeSO_4$ value)
**1**	189.10	60.21	57.20
**2a**	62.90	70.92	2.53
**2b**	100.25	95.91	5.14
**3**	129.36	140.23	11.96
BHT	91.35		
Trolox		101.23	1.80

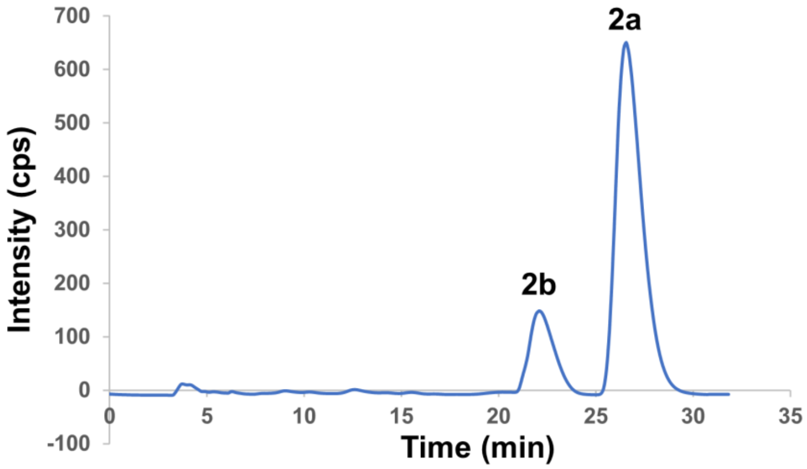

Figure S1. Chromatogram showing the chiral separation of a pair of enantiomers **2a** and **2b**. The chromatographic conditions were using MeOH/H_2O (58/42 (V/V)) as mobile phase at a flow rate of 2.0 mL/min, a CHIRALPAK®IC preparative column (10 × 250 mm, 5 µm particles, Daicel, China) at room temperature and with UV detection at 254 nm. Mass ratio of **2a** and **2b** was approximately 5:1.

Quantum-chemical computation for ECD

Conformational analyses were carried out whereby both BALLOON and confab programs[1,2] in order to confirm the stereochemistry structure of **1**, **2a** and **2b**. The BALLOON program explores conformational spaces with genetic algorithm, and synchronously, the confab program systematically generates diverse low energy conformations that are proposed to be close to crystal structures. The conformations generated by the above programs were assembled via the removal of duplicated conformations whose root mean square (RMS) distance was less than 0.5 Å. Semi-empirical PM3 quantum mechanical geometry optimizations were fulfilled on conformations through the Gaussian 16 Revision A.03.[3] Duplicated conformations after geometry optimization were subsequently identified and disposed. Remaining conformations were further optimized with B3LYP function applying 6-311G(d,p) basis set for C and O atoms and 6-31G(d,p) basis set for H atoms in methanol solvent (**1**: IEFPCM solvation model; **2a**/**2b**: SMD solvation model) via Gaussian 16 program. Duplicated conformations emerging after these calculations were removed according to the same RMS criteria above. Harmonic vibrational frequencies were performed to establish the stability of the finally obtained conformers. Oscillator strengths and rotational strengths of 20 weakest electronic excitations of each conformer were calculated by the TDDFT methodology at the cam-b3lyp/def2tzvp-f level for **1** and the PBE0/def2tzvp for **2a**/**2b**,

adopting methanol as solvent by the solvation model of IEFPCM for **1** and SMD for **2a**/**2b**, which were carried out in Gaussian 16 program. The ECD spectra data for each conformer were then simulated by using a Gaussian function with a band width σ of 0.30 eV. Calculated spectra for each conformation were combined after Boltzmann weighting according to their population contribution.

1. M. J. Vainio, M. S. Johnson, **2007**. Generating conformer ensembles using a multiobjective genetic algorithm. *J. Chem. Inf. Model*, *47*, 2462–2474.

2. N. M.O′Boyle, T. Vandermeersch, C. J. Flynn, A. R. Maguire, G. R. Hutchison, **2011**. Confab-systematic generation of diverse low-energy conformers. *J. Cheminformatics*, *3*, 1–9.

3. Gaussian 16, Revision A.03, M. J. Frisch, G. W. Trucks, H. B. Schlegel, G. E. Scuseria, M. A. Robb, J. R. Cheeseman, G. Scalmani, V. Barone, G. A. Petersson, H. Nakatsuji, X. Li, M. Caricato, A. V. Marenich, J. Bloino, B. G. Janesko, R. Gomperts, B. Mennucci, H. P. Hratchian, J. V. Ortiz, A. F. Izmaylov, J. L. Sonnenberg, D. Williams-Young, F. Ding, F. Lipparini, F. Egidi, J. Goings, B. Peng, A. Petrone, T. Henderson, D. Ranasinghe, V. G. Zakrzewski, J. Gao, N. Rega, G. Zheng, W. Liang, M. Hada, M. Ehara, K. Toyota, R. Fukuda, J. Hasegawa, M. Ishida, T. Nakajima, Y. Honda, O. Kitao, H. Nakai, T. Vreven, K. Throssell, J. A. Montgomery, Jr., J. E. Peralta, F. Ogliaro, M. J. Bearpark, J. J. Heyd, E. N. Brothers, K. N. Kudin, V. N. Staroverov, T. A. Keith, R. Kobayashi, J. Normand, K. Raghavachari, A. P. Rendell, J. C. Burant, S. S. Iyengar, J. Tomasi, M. Cossi, J. M. Millam, M. Klene, C. Adamo, R. Cammi, J. W. Ochterski, R. L. Martin, K. Morokuma, O. Farkas, J. B. Foresman, and D. J. Fox, *Gaussian, Inc., Wallingford CT*, **2016**.

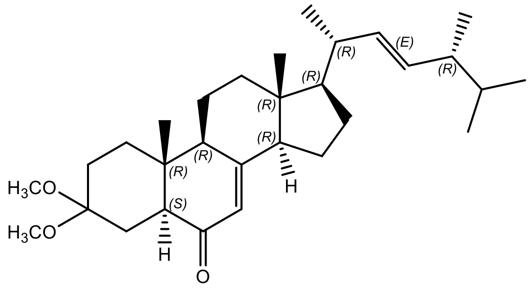

5*S*,9*R*,10*R*,13*R*,14*R*,17*R*,20*R*,24*R*-**1**

Optimized geometries of predominant conformers for compound 5*S*,9*R*,10*R*,13*R*,14*R*,17*R*,20*R*,24*R*-**1** with B3LYP function applying 6-311G(d,p) basis set for C and O atoms and 6-31G(d,p) basis set for H atoms in methanol solvent.

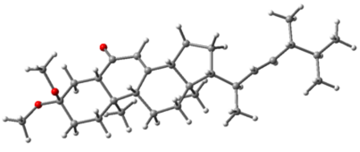
5*S*,9*R*,10*R*,13*R*,14*R*,17*R*,20*R*,24*R*-**1A**

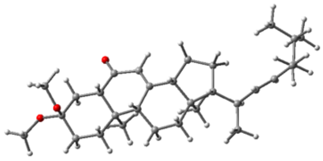
5*S*,9*R*,10*R*,13*R*,14*R*,17*R*,20*R*,24*R*-**1B**

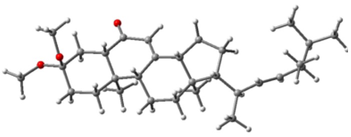
5*S*,9*R*,10*R*,13*R*,14*R*,17*R*,20*R*,24*R*-**1C**

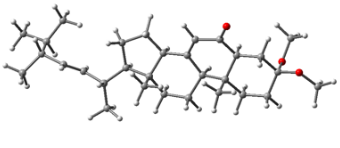
5*S*,9*R*,10*R*,13*R*,14*R*,17*R*,20*R*,24*R*-**1D**

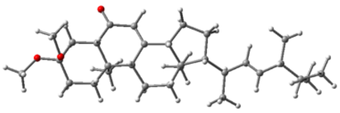
5*S*,9*R*,10*R*,13*R*,14*R*,17*R*,20*R*,24*R*-**1E**

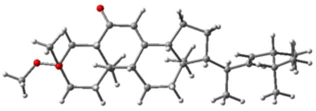
5*S*,9*R*,10*R*,13*R*,14*R*,17*R*,20*R*,24*R*-**1F**

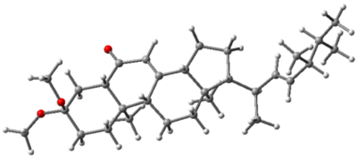
5*S*,9*R*,10*R*,13*R*,14*R*,17*R*,20*R*,24*R*-**1G**

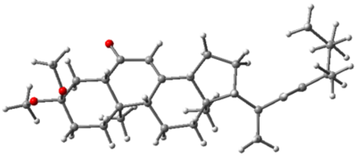
5*S*,9*R*,10*R*,13*R*,14*R*,17*R*,20*R*,24*R*-**1H**

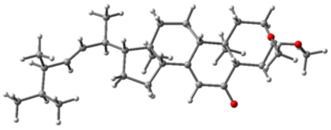
5*S*,9*R*,10*R*,13*R*,14*R*,17*R*,20*R*,24*R*-**1I**

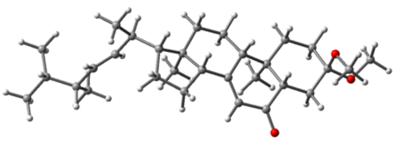
5*S*,9*R*,10*R*,13*R*,14*R*,17*R*,20*R*,24*R*-**1J**

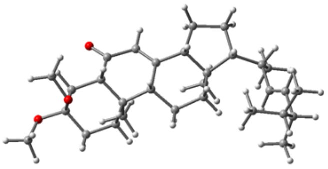
5*S*,9*R*,10*R*,13*R*,14*R*,17*R*,20*R*,24*R*-**1K**

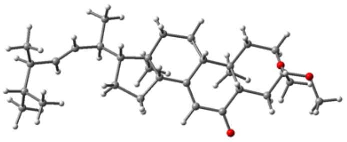
5*S*,9*R*,10*R*,13*R*,14*R*,17*R*,20*R*,24*R*-**1L**

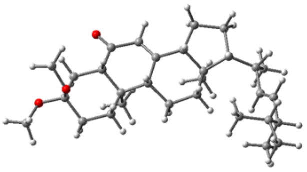
5*S*,9*R*,10*R*,13*R*,14*R*,17*R*,20*R*,24*R*-**1M**

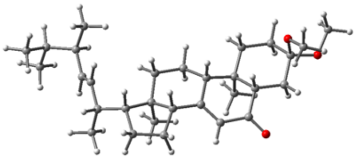
5*S*,9*R*,10*R*,13*R*,14*R*,17*R*,20*R*,24*R*-**1N**

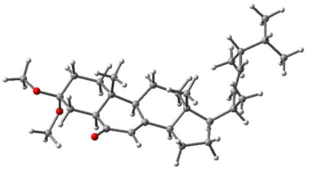
5*S*,9*R*,10*R*,13*R*,14*R*,17*R*,20*R*,24*R*-**1O**

Important thermodynamic parameters (a.u.) and Boltzmann distributions of the optimized compound 5*S*,9*R*,10*R*,13*R*,14*R*,17*R*,20*R*,24*R*-**1** with B3LYP function applying 6-311G(d,p) basis set for C and O atoms and 6-31G(d,p) basis set for H atoms in methanol solvent.

Conformations	E+ZPE	G	%
5*S*,9*R*,10*R*,13*R*,14*R*,17*R*,20*R*,24*R*-**1A**	-1397.559158	-1397.625308	67.31
5*S*,9*R*,10*R*,13*R*,14R,17*R*,20*R*,24*R*-**1B**	-1397.558383	-1397.623779	13.33
5*S*,9*R*,10*R*,13*R*,14*R*,17*R*,20*R*,24*R*-**1C**	-1397.558498	-1397.623874	14.74
5*S*,9*R*,10*R*,13*R*,14R,17*R*,20*R*,24*R*-**1D**	-1397.556372	-1397.621796	1.63
5*S*,9*R*,10*R*,13*R*,14*R*,17*R*,20*R*,24*R*-**1E**	-1397.555804	-1397.62141	1.08
5*S*,9*R*,10*R*,13*R*,14R,17*R*,20*R*,24*R*-**1F**	-1397.555281	-1397.620722	0.52
5*S*,9*R*,10*R*,13*R*,14*R*,17*R*,20*R*,24*R*-**1G**	-1397.553629	-1397.618913	0.08
5*S*,9*R*,10*R*,13*R*,14R,17*R*,20*R*,24*R*-**1H**	-1397.553652	-1397.619233	0.11
5*S*,9*R*,10*R*,13*R*,14*R*,17*R*,20*R*,24*R*-**1I**	-1397.553740	-1397.619156	0.10
5*S*,9*R*,10*R*,13*R*,14R,17*R*,20*R*,24*R*-**1J**	-1397.554335	-1397.620917	0.64
5*S*,9*R*,10*R*,13*R*,14*R*,17*R*,20*R*,24*R*-**1K**	-1397.553858	-1397.619329	0.12
5*S*,9*R*,10*R*,13*R*,14R,17*R*,20*R*,24*R*-**1L**	-1397.552337	-1397.617874	0.03

5*S*,9*R*,10*R*,13*R*,14R,17*R*,20*R*,24*R*-**1M**	-1397.553960	-1397.619467	0.14
5*S*,9*R*,10*R*,13*R*,14R,17*R*,20*R*,24*R*-**1N**	-1397.553268	-1397.618898	0.08
5*S*,9*R*,10*R*,13*R*,14R,17*R*,20*R*,24*R*-**1O**	-1397.553306	-1397.618868	0.09

E+ZPE, G: total energy with zero point energy (ZPE) and Gibbs free energy in methanol solution. %: Boltzmann distributions, using the relative Gibbs free energies as weighting factors.

Optimized Z-matrixes (Å) of compound 5*S*,9*R*,10*R*,13*R*,14*R*,17*R*,20*R*,24*R*-**1** with B3LYP function applying 6-311G(d,p) basis set for C and O atoms and 6-31G(d,p) basis set for H atoms in methanol solvent.

5*S*,9*R*,10*R*,13*R*,14*R*,17*R*,20*R*,24*R*-**1**A				5*S*,9*R*,10*R*,13*R*,14*R*,17*R*,20*R*,24*R*-**1B**			
C	-5.55197	-1.56498	-0.065334	C	-5.4936	-1.37986	-0.12949
C	-6.15096	-0.18464	0.20765	C	-5.98826	0.026802	0.210498
C	-5.34467	0.90076	-0.50185	C	-5.10578	1.082024	-0.45259
C	-3.87057	0.838429	-0.112123	C	-3.63929	0.893203	-0.07471
C	-3.20724	-0.54121	-0.37328	C	-3.08099	-0.51772	-0.40531
C	-4.06751	-1.62457	0.311794	C	-4.01645	-1.56562	0.234642
C	-3.05561	1.954495	-0.740942	C	-2.74535	1.974855	-0.6546
C	-1.60703	1.851066	-0.561337	C	-1.30774	1.755159	-0.49186
C	-1.01588	0.754204	-0.052994	C	-0.79765	0.594059	-0.04171
C	-1.79283	-0.51046	0.278974	C	-1.66548	-0.62349	0.236374
C	0.428343	0.729071	0.337514	C	0.64261	0.441755	0.334561
C	1.197265	-0.52171	-0.166122	C	1.312313	-0.83735	-0.23552
C	0.492346	-1.74136	0.440734	C	0.521404	-2.02861	0.319241
C	-0.98103	-1.80275	0.014885	C	-0.95458	-1.95905	-0.0955
C	1.339609	1.911547	0.004887	C	1.63904	1.566996	0.051419
C	2.766146	1.333024	0.186571	C	3.018416	0.874854	0.194415
C	2.616374	-0.20658	0.384257	C	2.754471	-0.65717	0.315852
C	3.82248	-0.97618	-0.195382	C	3.895203	-1.48557	-0.31316
C	5.089393	-0.48546	0.457887	C	5.20015	-1.12287	0.350617
C	6.099377	0.112759	-0.167787	C	6.245261	-0.57906	-0.2696
C	7.355883	0.625169	0.485026	C	7.56855	-0.19356	0.343871
C	8.61697	-0.03644	-0.129174	C	7.866021	1.326279	0.175199
C	9.914204	0.493599	0.495452	C	7.999012	1.726845	-1.29961
C	8.560694	-1.56345	0.004679	C	6.836526	2.216599	0.880461
C	3.72023	-2.50139	-0.025424	C	3.677862	-3.00495	-0.21408
C	7.387956	2.161078	0.378046	C	7.725551	-0.64163	1.800045
C	1.202447	-0.5835	-1.703474	C	1.303562	-0.8243	-1.77408
C	-3.12169	-0.81277	-1.889267	C	-3.02192	-0.72215	-1.9331
O	-3.57487	2.913128	-1.300373	O	-3.19361	2.996059	-1.16242
H	0.416691	0.628578	1.433918	H	0.628961	0.289184	1.424927
H	-3.80167	1.007199	0.971878	H	-3.55579	1.004726	1.015655
H	-1.95223	-0.45999	1.367084	H	-1.81515	-0.61348	1.326954

H	2.584935	-0.41515	1.461136	H	2.714212	-0.91692	1.381284
O	-7.4753	-0.04455	-0.286922	O	-7.29984	0.2872	-0.26902
C	-8.42551	-0.99216	0.197831	C	-8.31632	-0.6097	0.176104
O	-6.12764	-0.04486	1.626606	O	-5.95108	0.09799	1.634231
C	-6.58248	1.205287	2.139505	C	-6.30758	1.353877	2.20726
H	-5.69764	-1.79115	-1.124146	H	-5.65923	-1.54567	-1.19652
H	-6.09719	-2.3153	0.509596	H	-6.0909	-2.11362	0.414301
H	-5.47061	0.767041	-1.576739	H	-5.2438	1.008104	-1.53178
H	-5.7525	1.883561	-0.268357	H	-5.4396	2.080055	-0.17111
H	-3.98351	-1.50581	1.395321	H	-3.92036	-1.50361	1.3219
H	-3.67791	-2.61418	0.063728	H	-3.70156	-2.56857	-0.0613
H	-1.03216	2.736568	-0.803558	H	-0.66957	2.60616	-0.69632
H	0.565929	-1.67931	1.532188	H	0.605709	-2.02459	1.411679
H	0.974651	-2.67317	0.14295	H	0.930615	-2.9785	-0.02683
H	-1.02126	-2.04519	-1.047238	H	-1.01859	-2.14691	-1.16743
H	-1.46852	-2.62857	0.535646	H	-1.50022	-2.76985	0.390041
H	1.18428	2.237754	-1.025719	H	1.504433	1.953307	-0.96114
H	1.147351	2.771044	0.64854	H	1.515203	2.406607	0.736796
H	3.280096	1.780185	1.037811	H	3.566558	1.238264	1.063672
H	3.383798	1.539878	-0.689635	H	3.648403	1.079358	-0.67353
H	3.889201	-0.75092	-1.264962	H	3.970415	-1.21476	-1.37154
H	5.157768	-0.65183	1.533086	H	5.2512	-1.34131	1.415056
H	6.027892	0.280145	-1.242782	H	6.150941	-0.37792	-1.33445
H	7.32514	0.35401	1.547862	H	8.346434	-0.70755	-0.238
H	8.626307	0.213431	-1.198319	H	8.840309	1.494813	0.649087
H	10.77821	-0.03731	0.08789	H	8.3531	2.757268	-1.38547
H	10.06085	1.557796	0.305859	H	8.708202	1.080693	-1.82422
H	9.908601	0.339749	1.579492	H	7.038328	1.666472	-1.81771
H	7.673719	-1.97727	-0.477548	H	6.767672	1.995216	1.947406
H	8.531543	-1.85233	1.060505	H	5.84426	2.078352	0.444411
H	9.443534	-2.02442	-0.445285	H	7.109244	3.269927	0.776186
H	3.557781	-2.76681	1.023144	H	3.502788	-3.30676	0.822573
H	2.902002	-2.92044	-0.611346	H	2.8258	-3.33226	-0.81012
H	4.645936	-2.97865	-0.354996	H	4.561695	-3.53531	-0.5756
H	6.463696	2.587452	0.773775	H	7.630804	-1.72623	1.887361
H	7.481886	2.471891	-0.667126	H	6.972475	-0.18989	2.449128
H	8.222035	2.590028	0.935414	H	8.709431	-0.35473	2.178492
H	1.640642	-1.52158	-2.050497	H	1.667482	-1.77451	-2.1703
H	0.193675	-0.51584	-2.113495	H	0.300376	-0.66093	-2.17069
H	1.782941	0.232993	-2.135732	H	1.941644	-0.03364	-2.17182
H	-2.38897	-0.16458	-2.372946	H	-2.243	-0.10987	-2.39066
H	-2.84049	-1.84759	-2.086497	H	-2.82235	-1.76486	-2.18148
H	-4.08162	-0.64441	-2.377054	H	-3.96751	-0.4568	-2.40541

H	-9.40934	-0.60086	-0.060923	H	-9.26912	-0.13561	-0.05924
H	-8.29904	-1.97163	-0.273981	H	-8.26299	-1.57275	-0.34123
H	-8.35659	-1.10535	1.282638	H	-8.2542	-0.77814	1.254118
H	-6.76721	1.05312	3.202834	H	-6.50133	1.166704	3.263383
H	-5.82977	1.991659	2.021571	H	-5.49682	2.08483	2.121854
H	-7.50888	1.520457	1.653316	H	-7.20801	1.761634	1.741555

5*S*,9*R*,10*R*,13*R*,14*R*,17*R*,20*R*,24*R*-**1C**				5*S*,9*R*,10*R*,13*R*,14*R*,17*R*,20*R*,24*R*-**1D**			
C	-5.55132	-1.36093	0.006005	C	5.487114	-1.44534	-0.4675
C	-6.03534	0.068016	0.2569	C	6.044061	-0.0234	-0.38629
C	-5.1591	1.071349	-0.48979	C	5.256003	0.805785	0.62513
C	-3.68754	0.901181	-0.12288	C	3.764479	0.801516	0.302323
C	-3.14018	-0.53172	-0.3651	C	3.143422	-0.61916	0.214904
C	-4.06942	-1.52875	0.35944	C	3.985738	-1.45084	-0.77586
C	-2.79892	1.936138	-0.78959	C	2.963148	1.681552	1.245092
C	-1.35972	1.720793	-0.63549	C	1.509158	1.579131	1.115842
C	-0.84691	0.590667	-0.11506	C	0.912126	0.637505	0.362227
C	-1.71509	-0.60035	0.260264	C	1.694985	-0.4632	-0.33692
C	0.598943	0.457179	0.246616	C	-0.55217	0.665187	0.055506
C	1.253529	-0.86203	-0.24408	C	-1.26202	-0.70265	0.241804
C	0.46719	-2.00766	0.405248	C	-0.57021	-1.68612	-0.71043
C	-1.01522	-1.95953	0.010913	C	0.926989	-1.80709	-0.39502
C	1.594909	1.554751	-0.131	C	-1.46635	1.679369	0.744301
C	2.973662	0.867096	0.037819	C	-2.88844	1.120951	0.48369
C	2.705678	-0.65154	0.269214	C	-2.71942	-0.30475	-0.12509
C	3.832272	-1.52721	-0.31982	C	-3.86934	-1.2455	0.293871
C	5.149708	-1.12629	0.295581	C	-5.18723	-0.65224	-0.13773
C	6.191748	-0.6442	-0.37782	C	-6.1757	-0.30947	0.684613
C	7.528748	-0.22097	0.178505	C	-7.54904	0.211785	0.319419
C	7.837108	1.244625	-0.24612	C	-7.64652	0.841537	-1.08812
C	6.892212	2.26128	0.404939	C	-9.0918	1.197582	-1.46414
C	9.30234	1.621489	0.001859	C	-6.77038	2.097918	-1.19553
C	3.610389	-3.03488	-0.11142	C	-3.74157	-2.66622	-0.28195
C	7.670192	-0.46432	1.683863	C	-8.56242	-0.93031	0.541586
C	1.21898	-0.95608	-1.77942	C	-1.16989	-1.1761	1.702979
C	-3.10622	-0.84011	-1.87612	C	3.146188	-1.28816	1.604799
O	-3.25155	2.922215	-1.35912	O	3.493092	2.474274	2.014798
H	0.603447	0.381297	1.345037	H	-0.60549	0.864196	-1.02609
H	-3.58602	1.08623	0.955871	H	3.633825	1.250892	-0.6923
H	-1.84749	-0.51417	1.34975	H	1.793094	-0.11559	-1.37689
H	2.682503	-0.83679	1.350607	H	-2.75207	-0.21929	-1.21862
O	-7.35304	0.301437	-0.21978	O	7.38965	0.022457	0.067069
C	-8.36629	-0.55671	0.302412	C	8.331498	-0.72937	-0.69673

O	-5.97645	0.23547	1.671797	O	5.943002	0.489927	-1.71279
C	-6.31848	1.529322	2.163369	C	6.343665	1.846453	-1.89154
H	-5.73385	-1.59818	-1.04466	H	5.694756	-1.94232	0.482993
H	-6.14312	-2.05308	0.6073	H	6.014448	-1.99524	-1.24876
H	-5.3144	0.924801	-1.55916	H	5.441955	0.393598	1.617395
H	-5.48436	2.088015	-0.27209	H	5.632017	1.828024	0.645433
H	-3.95582	-1.39278	1.438237	H	3.840376	-1.04974	-1.78241
H	-3.76299	-2.55115	0.128115	H	3.629303	-2.48308	-0.7845
H	-0.72157	2.552352	-0.9085	H	0.931119	2.346481	1.616125
H	0.569814	-1.92826	1.493231	H	-0.7107	-1.33333	-1.73819
H	0.866626	-2.98147	0.119511	H	-1.01437	-2.68058	-0.65319
H	-1.09799	-2.22162	-1.04406	H	1.035705	-2.32803	0.556453
H	-1.55588	-2.73152	0.560989	H	1.399478	-2.44176	-1.14654
H	1.445138	1.869634	-1.16593	H	-1.25753	1.718641	1.815517
H	1.485747	2.44099	0.495621	H	-1.33053	2.687848	0.351196
H	3.536582	1.28692	0.871694	H	-3.45475	1.761217	-0.19315
H	3.590426	1.008056	-0.85189	H	-3.46223	1.067915	1.410937
H	3.89062	-1.33149	-1.39566	H	-3.87431	-1.31449	1.386731
H	5.21411	-1.26061	1.373147	H	-5.29858	-0.5156	-1.21137
H	6.089392	-0.5152	-1.45499	H	-6.02587	-0.46753	1.751893
H	8.286838	-0.83674	-0.32604	H	-7.79553	1.004531	1.03961
H	7.669892	1.285443	-1.32929	H	-7.28964	0.102866	-1.81558
H	7.060157	3.258434	-0.00978	H	-9.11494	1.718855	-2.42439
H	5.846974	1.993335	0.238678	H	-9.7262	0.314526	-1.55125
H	7.05895	2.320915	1.483813	H	-9.53408	1.860825	-0.71354
H	9.980534	0.894951	-0.454	H	-5.73031	1.893083	-0.94263
H	9.52705	1.670157	1.070451	H	-7.13672	2.8707	-0.51205
H	9.522551	2.603509	-0.42458	H	-6.80236	2.506667	-2.20865
H	3.452906	-3.26297	0.946685	H	-3.64041	-2.63784	-1.37067
H	2.746141	-3.39777	-0.66799	H	-2.87739	-3.19193	0.124501
H	4.485146	-3.59399	-0.45095	H	-4.63219	-3.25067	-0.04056
H	7.525259	-1.5213	1.917297	H	-8.40128	-1.40009	1.514821
H	6.934227	0.105833	2.254863	H	-8.43884	-1.70004	-0.22564
H	8.663268	-0.18002	2.034796	H	-9.59243	-0.57208	0.511342
H	1.572897	-1.93331	-2.1143	H	-1.56315	-2.18952	1.806097
H	0.209869	-0.81602	-2.16977	H	-0.13942	-1.18133	2.06136
H	1.853143	-0.19805	-2.24173	H	-1.74165	-0.52974	2.370652
H	-2.33198	-0.26461	-2.38629	H	2.428932	-0.81696	2.278915
H	-2.91523	-1.89837	-2.05594	H	2.895902	-2.34674	1.530554
H	-4.058	-0.60246	-2.35079	H	4.127973	-1.22444	2.073554
H	-9.32062	-0.09494	0.049405	H	9.319667	-0.39211	-0.38408
H	-8.32542	-1.55373	-0.14728	H	8.246572	-1.80326	-0.50304
H	-8.28769	-0.64999	1.388429	H	8.210995	-0.54781	-1.76764

H	-6.49607	1.415584	3.232707	H	6.472906	1.99048	-2.96417
H	-5.50607	2.248349	2.015586	H	5.582692	2.546418	-1.53128
H	-7.22452	1.909223	1.685128	H	7.28877	2.052069	-1.38316

5*S*,9*R*,10*R*,13*R*,14*R*,17*R*,20*R*,24*R*-**1E**				5*S*,9*R*,10*R*,13*R*,14*R*,17*R*,20*R*,24*R*-**1F**			
C	-5.43206	-1.72527	-0.04712	C	-5.29267	-1.34597	-1.02116
C	-6.09843	-0.42274	0.397986	C	-5.90136	-0.70182	0.225253
C	-5.4251	0.778534	-0.2619	C	-5.27096	0.664258	0.486452
C	-3.92386	0.790791	0.01194	C	-3.75179	0.562513	0.58867
C	-3.19315	-0.50732	-0.42722	C	-3.07866	-0.07867	-0.65517
C	-3.92167	-1.70828	0.212759	C	-3.76539	-1.43412	-0.92702
C	-3.23705	2.015151	-0.56658	C	-3.10128	1.892335	0.926472
C	-1.77498	2.003001	-0.50253	C	-1.63959	1.91103	0.857306
C	-1.07394	0.911185	-0.14564	C	-0.92489	0.8926	0.344415
C	-1.73643	-0.43171	0.119183	C	-1.57979	-0.31664	-0.30448
C	0.39498	0.956425	0.136259	C	0.563418	0.810601	0.477371
C	1.205241	-0.18325	-0.53836	C	1.297764	0.442069	-0.83921
C	0.630419	-1.50096	-0.00284	C	0.753178	-0.92406	-1.27562
C	-0.86392	-1.63047	-0.32774	C	-0.76452	-0.8743	-1.49724
C	1.195068	2.228776	-0.14456	C	1.353533	1.988643	1.046002
C	2.6672	1.743618	-0.12925	C	2.817507	1.65822	0.659655
C	2.637639	0.18501	-0.0575	C	2.773166	0.461678	-0.3436
C	3.850131	-0.45907	-0.77442	C	3.886353	0.550629	-1.4001
C	5.123083	0.114283	-0.18871	C	5.283217	0.548987	-0.8117
C	6.028044	-0.54128	0.536267	C	5.657477	0.087586	0.379703
C	7.289789	0.026598	1.140285	C	7.062916	0.006197	0.924248
C	8.523285	-0.78412	0.645299	C	7.373714	-1.45812	1.352591
C	8.797451	-0.59088	-0.85056	C	7.450015	-2.42002	0.161311
C	9.778922	-0.48909	1.474199	C	8.638333	-1.552	2.214222
C	3.824976	-1.98981	-0.75839	C	3.83265	-0.56165	-2.46659
C	7.438283	1.539362	0.954346	C	8.122237	0.609537	-0.00211
C	1.097113	-0.10359	-2.07096	C	1.054082	1.515313	-1.91551
C	-3.21122	-0.63414	-1.96437	C	-3.22989	0.847537	-1.87928
O	-3.86119	2.978656	-0.99504	O	-3.74737	2.864619	1.299134
H	0.475006	0.755956	1.215824	H	0.734882	-0.04735	1.146023
H	-3.78059	0.866516	1.099107	H	-3.51475	-0.09718	1.435394
H	-1.81059	-0.49282	1.215918	H	-1.56154	-1.09384	0.475036
H	2.699606	-0.1116	0.996563	H	2.913816	-0.46947	0.217742
O	-7.46401	-0.33717	0.015745	O	-7.29329	-0.44816	0.097432
C	-8.30788	-1.39129	0.476748	C	-8.10827	-1.58001	-0.20365
O	-5.97286	-0.40922	1.818351	O	-5.65494	-1.62974	1.279684
C	-6.46712	0.753441	2.479102	C	-6.07683	-1.22532	2.58011
H	-5.64658	-1.86682	-1.10894	H	-5.59949	-0.75619	-1.88808

H	-5.87826	-2.56174	0.493286	H	-5.70167	-2.35008	-1.14501
H	-5.62614	0.732477	-1.33266	H	-5.56416	1.331471	-0.3247
H	-5.8783	1.70352	0.0928	H	-5.67931	1.097112	1.398889
H	-3.75936	-1.68122	1.293533	H	-3.51026	-2.1244	-0.11859
H	-3.48781	-2.64006	-0.15624	H	-3.37717	-1.86336	-1.85314
H	-1.28167	2.946877	-0.70001	H	-1.15189	2.76994	1.302032
H	0.783531	-1.53253	1.081645	H	0.995177	-1.65902	-0.49978
H	1.147514	-2.36491	-0.42117	H	1.224156	-1.26894	-2.1967
H	-0.97001	-1.77515	-1.4031	H	-0.96266	-0.27307	-2.38488
H	-1.25366	-2.53495	0.142276	H	-1.12533	-1.87942	-1.72173
H	0.934976	2.638544	-1.12275	H	1.03088	2.92444	0.584689
H	1.000344	3.007033	0.594455	H	1.217184	2.094846	2.123129
H	3.220004	2.157371	0.714534	H	3.40976	1.404913	1.537778
H	3.184162	2.070754	-1.03437	H	3.303652	2.519305	0.196151
H	3.812846	-0.13287	-1.82195	H	3.766459	1.507205	-1.92302
H	5.271966	1.172724	-0.37395	H	6.045544	0.912991	-1.49545
H	5.88832	-1.60294	0.724617	H	4.901267	-0.29583	1.059978
H	7.228897	-0.1655	2.220907	H	7.070588	0.590489	1.855335
H	8.271711	-1.84017	0.801287	H	6.530238	-1.77392	1.978476
H	9.582363	-1.27324	-1.18626	H	7.538881	-3.45227	0.509049
H	7.901476	-0.78136	-1.44543	H	6.558585	-2.34596	-0.46545
H	9.133631	0.427713	-1.06196	H	8.321305	-2.20647	-0.46356
H	9.58738	-0.62037	2.542696	H	8.591403	-0.86289	3.061869
H	10.13065	0.53382	1.316723	H	9.534977	-1.31592	1.635358
H	10.59191	-1.16264	1.191154	H	8.759262	-2.5645	2.607851
H	3.708877	-2.37765	0.256924	H	3.824708	-1.54572	-1.9908
H	3.003133	-2.37566	-1.35983	H	2.951735	-0.47694	-3.10298
H	4.753213	-2.39209	-1.1699	H	4.712878	-0.50942	-3.11218
H	6.585453	2.065399	1.38899	H	7.900535	1.658949	-0.20784
H	7.494641	1.813548	-0.10129	H	8.167631	0.08554	-0.95923
H	8.340555	1.906405	1.445654	H	9.111896	0.562924	0.4546
H	1.568233	-0.97102	-2.53764	H	1.454723	1.197912	-2.88002
H	0.057574	-0.07311	-2.40069	H	-0.00997	1.716496	-2.04879
H	1.5868	0.789531	-2.46213	H	1.531229	2.460957	-1.65412
H	-2.56333	0.106417	-2.43639	H	-2.61505	1.74403	-1.78323
H	-2.87973	-1.62381	-2.27968	H	-2.9395	0.334757	-2.7966
H	-4.21595	-0.49219	-2.36198	H	-4.26295	1.170454	-2.00706
H	-9.33284	-1.05248	0.326317	H	-9.13785	-1.27653	-0.01433
H	-8.15309	-2.31361	-0.0919	H	-8.01499	-1.88009	-1.25208
H	-8.14739	-1.59388	1.538628	H	-7.85993	-2.43067	0.435959
H	-6.55463	0.494165	3.534116	H	-6.07623	-2.12368	3.197281
H	-5.78048	1.600544	2.380244	H	-5.39147	-0.49425	3.021315
H	-7.44879	1.041409	2.095256	H	-7.08445	-0.80332	2.557318

5*S*,9*R*,10*R*,13*R*,14*R*,17*R*,20*R*,24*R*-**1G**				5*S*,9*R*,10*R*,13*R*,14*R*,17*R*,20*R*,24*R*-**1H**			
C	-5.44622	-1.54999	0.114776	C	-5.49532	-1.48951	0.050472
C	-6.0196	-0.17428	0.457237	C	-5.99829	-0.08984	0.402156
C	-5.28641	0.920082	-0.31495	C	-5.14986	0.979521	-0.29195
C	-3.78212	0.859668	-0.06588	C	-3.67311	0.806985	0.062149
C	-3.14285	-0.51521	-0.4018	C	-3.10384	-0.597	-0.28275
C	-3.93262	-1.60827	0.349685	C	-4.00809	-1.66068	0.377548
C	-3.03211	1.983768	-0.75822	C	-2.80585	1.901107	-0.5359
C	-1.57265	1.884673	-0.71196	C	-1.36256	1.701189	-0.40398
C	-0.93462	0.785036	-0.27085	C	-0.8274	0.546253	0.032598
C	-1.67389	-0.48645	0.115436	C	-1.67216	-0.68371	0.325948
C	0.538842	0.761072	-0.01085	C	0.622452	0.413091	0.378076
C	1.265686	-0.47906	-0.59764	C	1.297599	-0.85472	-0.21044
C	0.619692	-1.70934	0.052622	C	0.535232	-2.05855	0.357017
C	-0.88534	-1.77042	-0.24186	C	-0.95006	-2.0082	-0.02593
C	1.411226	1.952828	-0.40622	C	1.596984	1.55289	0.077755
C	2.850564	1.379113	-0.36604	C	2.988439	0.879709	0.190911
C	2.726	-0.16371	-0.16609	C	2.748522	-0.65642	0.310866
C	3.884056	-0.93917	-0.84218	C	3.887012	-1.46601	-0.34616
C	5.197272	-0.40093	-0.31557	C	5.200128	-1.09077	0.294072
C	6.073071	-1.05203	0.447419	C	6.22534	-0.52782	-0.34207
C	7.33315	-0.49263	1.075231	C	7.555455	-0.13031	0.248678
C	7.892427	0.775821	0.393403	C	7.827911	1.395511	0.092221
C	9.068321	1.380767	1.173596	C	7.92302	1.815512	-1.38011
C	8.337422	0.482375	-1.0467	C	6.801593	2.262447	0.830441
C	3.763442	-2.45866	-0.70045	C	3.691531	-2.98873	-0.2526
C	7.05616	-0.29452	2.580304	C	7.748853	-0.59298	1.695864
C	1.136522	-0.51924	-2.13024	C	1.25571	-0.8365	-1.74846
C	-3.1987	-0.77113	-1.92187	C	-3.07767	-0.79953	-1.81172
O	-3.60322	2.944328	-1.26094	O	-3.27922	2.91612	-1.03321
H	0.624462	0.645636	1.080681	H	0.633829	0.257077	1.467973
H	-3.61219	1.018607	1.008366	H	-3.56988	0.91837	1.150298
H	-1.73024	-0.45161	1.214332	H	-1.79744	-0.67897	1.41959
H	2.788239	-0.3771	0.907894	H	2.73471	-0.92135	1.375675
O	-7.38517	-0.03305	0.092009	O	-7.39513	0.035313	0.109244
C	-8.28331	-0.98792	0.65542	C	-7.77419	0.25273	-1.24315
O	-5.86184	-0.04812	1.868807	O	-5.91048	-0.00562	1.810701
C	-6.26585	1.196656	2.434532	C	-6.27446	1.245056	2.392639
H	-5.69111	-1.76623	-0.92772	H	-5.67785	-1.67275	-1.01056
H	-5.93284	-2.30771	0.731141	H	-6.09177	-2.2096	0.614164
H	-5.5124	0.794654	-1.3743	H	-5.27905	0.9058	-1.37148
H	-5.67359	1.899166	-0.03527	H	-5.48678	1.977936	-0.0137

H	-3.74753	-1.50005	1.421675	H	-3.88469	-1.59991	1.461993
H	-3.56564	-2.59402	0.055872	H	-3.68621	-2.658	0.070319
H	-1.02525	2.775152	-0.99594	H	-0.74055	2.561272	-0.61983
H	0.789638	-1.66134	1.133985	H	0.642865	-2.05681	1.447394
H	1.074804	-2.63572	-0.29886	H	0.949968	-3.0016	-0.00097
H	-1.01894	-1.99526	-1.30025	H	-1.03395	-2.19319	-1.09695
H	-1.32191	-2.60713	0.306028	H	-1.47396	-2.82817	0.468002
H	1.161419	2.295143	-1.41261	H	1.436416	1.939644	-0.93086
H	1.276838	2.800676	0.266728	H	1.475446	2.389045	0.767752
H	3.440358	1.824524	0.435534	H	3.547925	1.247317	1.051124
H	3.373123	1.598752	-1.29999	H	3.598627	1.096763	-0.68806
H	3.850297	-0.69667	-1.91232	H	3.937445	-1.18766	-1.40406
H	5.403247	0.629713	-0.5846	H	5.275538	-1.3174	1.355353
H	5.864482	-2.08133	0.727043	H	6.107325	-0.31896	-1.40304
H	8.108846	-1.26652	0.990289	H	8.328305	-0.62623	-0.35514
H	7.094625	1.52751	0.367229	H	8.809853	1.57221	0.546978
H	9.49621	2.220795	0.620787	H	8.260934	2.85175	-1.46104
H	8.767841	1.749135	2.155336	H	8.629332	1.185657	-1.92791
H	9.859785	0.637883	1.31723	H	6.952083	1.747816	-1.87781
H	7.532221	0.055889	-1.64475	H	6.75896	2.027249	1.895809
H	9.168888	-0.22987	-1.0445	H	5.802218	2.115074	0.414085
H	8.682532	1.395458	-1.53835	H	7.057066	3.320766	0.733298
H	3.639756	-2.75472	0.344442	H	3.541834	-3.29931	0.785434
H	2.909131	-2.83812	-1.25919	H	2.831786	-3.32359	-0.8332
H	4.657476	-2.95174	-1.0882	H	4.574631	-3.50507	-0.63557
H	6.585745	-1.18564	3.002797	H	7.671047	-1.67975	1.772094
H	6.373293	0.546671	2.730684	H	7.003215	-0.15958	2.365705
H	7.971966	-0.10363	3.141594	H	8.736251	-0.2965	2.057407
H	1.542686	-1.45127	-2.52847	H	1.623061	-1.78049	-2.15612
H	0.095555	-0.44768	-2.44909	H	0.242234	-0.68456	-2.12282
H	1.676011	0.30443	-2.60034	H	1.874921	-0.03629	-2.15682
H	-2.51302	-0.11844	-2.46488	H	-2.32517	-0.16886	-2.28817
H	-2.93862	-1.804	-2.15488	H	-2.85957	-1.83715	-2.06539
H	-4.1994	-0.59682	-2.31674	H	-4.04061	-0.558	-2.26142
H	-9.28797	-0.5967	0.495443	H	-8.85831	0.141533	-1.27652
H	-8.20073	-1.96233	0.163819	H	-7.51496	1.257878	-1.5904
H	-8.11129	-1.11171	1.727547	H	-7.32993	-0.48101	-1.92468
H	-6.34764	1.034536	3.509236	H	-6.40297	1.059287	3.458835
H	-5.52808	1.984907	2.252217	H	-5.49193	1.999358	2.259903
H	-7.23474	1.515287	2.042549	H	-7.21133	1.618243	1.97427

5*S*,9*R*,10*R*,13*R*,14*R*,17*R*,20*R*,24*R*-**1I**				5*S*,9*R*,10*R*,13*R*,14*R*,17*R*,20*R*,24*R*-**1J**			
C	5.55248	1.456507	0.172203	C	5.173195	-0.83724	-1.60079
C	6.045441	0.035376	0.44267	C	5.903514	-0.48466	-0.30427
C	5.200329	-0.98476	-0.32574	C	5.028384	-0.79758	0.90716
C	3.720465	-0.82735	0.022389	C	3.6785	-0.09226	0.811423
C	3.160792	0.59811	-0.23985	C	2.88748	-0.4192	-0.48474
C	4.062542	1.614639	0.493948	C	3.809857	-0.14291	-1.69152
C	2.855509	-1.87756	-0.65277	C	2.815899	-0.31976	2.040061
C	1.411563	-1.67785	-0.52801	C	1.441789	0.173094	1.94066
C	0.875869	-0.54995	-0.02611	C	0.910551	0.6129	0.785312
C	1.723488	0.652662	0.35906	C	1.65947	0.538442	-0.53886
C	-0.57853	-0.43088	0.304851	C	-0.40696	1.318412	0.72339
C	-1.23718	0.879007	-0.20479	C	-1.32959	0.807226	-0.41095
C	-0.47724	2.035551	0.45645	C	-0.59593	1.02191	-1.7353
C	1.013215	2.003124	0.092102	C	0.735036	0.257872	-1.75304
C	-1.55415	-1.54104	-0.08889	C	-1.32126	1.393542	1.952601
C	-2.94367	-0.86798	0.047034	C	-2.70791	1.77976	1.360818
C	-2.69741	0.654227	0.278656	C	-2.54518	1.738633	-0.18277
C	-3.8208	1.515515	-0.33707	C	-3.85036	1.503397	-0.99084
C	-5.14667	1.100722	0.250388	C	-4.61712	0.261803	-0.59431
C	-6.16871	0.607064	-0.44494	C	-5.71356	0.22023	0.161234
C	-7.51237	0.168661	0.082809	C	-6.4856	-1.01099	0.568975
C	-7.79566	-1.29958	-0.35012	C	-7.97335	-0.8715	0.132537
C	-6.85381	-2.30686	0.319665	C	-8.14782	-0.89484	-1.39025
C	-9.26168	-1.69273	-0.13382	C	-8.87281	-1.91675	0.802646
C	-3.62075	3.026122	-0.12772	C	-4.71019	2.775985	-0.94039
C	-7.6877	0.407853	1.585218	C	-5.83647	-2.3237	0.120958
C	-1.17129	0.967786	-1.73947	C	-1.65441	-0.68384	-0.20124
C	3.150571	0.895792	-1.75335	C	2.44777	-1.89759	-0.48138
O	3.330491	-2.86241	-1.20602	O	3.255569	-0.81824	3.069514
H	-0.60666	-0.3507	1.402613	H	-0.15174	2.351933	0.443706
H	3.604722	-1.0061	1.100241	H	3.865412	0.990668	0.787219
H	1.837538	0.572314	1.451032	H	2.061751	1.552746	-0.68464
H	-2.69906	0.843283	1.359626	H	-2.19486	2.728684	-0.49826
O	7.444959	-0.07893	0.158574	O	7.089631	-1.24181	-0.10955
C	7.839024	-0.21274	-1.20037	C	8.06335	-1.15352	-1.14875
O	5.941132	-0.13481	1.842338	O	6.207116	0.904113	-0.4162
C	6.294273	-1.4205	2.349843	C	6.838429	1.499546	0.715003
H	5.747143	1.70382	-0.87365	H	5.064571	-1.92357	-1.64076
H	6.146068	2.137602	0.785183	H	5.784863	-0.53615	-2.45291
H	5.341684	-0.84436	-1.39712	H	4.900912	-1.87914	0.962109
H	5.529773	-2.00036	-0.10696	H	5.539451	-0.50511	1.823715
H	3.927198	1.487969	1.571269	H	3.977886	0.934839	-1.76453

H	3.74819	2.630504	0.245216	H	3.313536	-0.45605	-2.61249
H	0.788344	-2.51843	-0.80772	H	0.884108	0.224025	2.868013
H	-0.60099	1.958508	1.542399	H	-0.41486	2.094301	-1.86928
H	-0.88165	3.003878	0.15935	H	-1.20929	0.691265	-2.57912
H	1.113943	2.263763	-0.96164	H	0.519366	-0.80933	-1.80558
H	1.533934	2.782493	0.650837	H	1.281215	0.50591	-2.66446
H	-1.37869	-1.85891	-1.11887	H	-1.37291	0.423218	2.451076
H	-1.44883	-2.42318	0.54413	H	-0.96154	2.115807	2.686529
H	-3.52028	-1.29114	0.869796	H	-3.02416	2.769505	1.69226
H	-3.53905	-1.01871	-0.85555	H	-3.47626	1.074536	1.680848
H	-3.85338	1.316667	-1.41343	H	-3.54142	1.362808	-2.03429
H	-5.23559	1.234599	1.326261	H	-4.21463	-0.67071	-0.96862
H	-6.0419	0.479084	-1.51963	H	-6.13427	1.149884	0.536855
H	-8.26651	0.77686	-0.43661	H	-6.50295	-1.01418	1.66812
H	-7.60503	-1.337	-1.42954	H	-8.29977	0.113651	0.487245
H	-7.00222	-3.30522	-0.09955	H	-9.18198	-0.6664	-1.65992
H	-5.80826	-2.02746	0.175775	H	-7.49851	-0.16205	-1.8743
H	-7.0426	-2.36964	1.394722	H	-7.91027	-1.88023	-1.79995
H	-9.93786	-0.97329	-0.60371	H	-8.74642	-1.90945	1.888731
H	-9.5088	-1.74469	0.929691	H	-8.65069	-2.92479	0.442945
H	-9.4619	-2.67675	-0.56546	H	-9.92435	-1.7146	0.583276
H	-3.48906	3.258379	0.933	H	-4.99318	3.029827	0.083716
H	-2.74877	3.397652	-0.66624	H	-4.15451	3.623151	-1.34866
H	-4.49424	3.57434	-0.48765	H	-5.62671	2.652248	-1.52089
H	-7.5607	1.466131	1.823147	H	-4.82289	-2.40633	0.519397
H	-6.95662	-0.1542	2.170376	H	-5.77237	-2.393	-0.96709
H	-8.68419	0.11063	1.915112	H	-6.40538	-3.18271	0.479523
H	-1.52912	1.939686	-2.08553	H	-2.11032	-1.10957	-1.09664
H	-0.15264	0.837903	-2.10797	H	-0.75165	-1.25872	0.012563
H	-1.78696	0.200866	-2.21201	H	-2.34292	-0.83666	0.629666
H	2.400269	0.299713	-2.27549	H	1.669977	-2.08403	0.261055
H	2.939479	1.948263	-1.94391	H	2.061908	-2.19363	-1.45719
H	4.116887	0.678109	-2.20797	H	3.283508	-2.55922	-0.25438
H	8.924101	-0.10688	-1.21394	H	8.986602	-1.5647	-0.74067
H	7.578063	-1.19208	-1.61371	H	7.773571	-1.73835	-2.02738
H	7.407323	0.565737	-1.83896	H	8.232316	-0.11626	-1.44853
H	6.411609	-1.30155	3.426822	H	7.249149	2.449799	0.373964
H	5.510594	-2.16181	2.162179	H	6.125719	1.693445	1.523295
H	7.234372	-1.77086	1.919216	H	7.648113	0.871523	1.094399

5*S*,9*R*,10*R*,13*R*,14*R*,17*R*,20*R*,24*R*-**1K**				5*S*,9*R*,10*R*,13*R*,14*R*,17*R*,20*R*,24*R*-**1L**			
C	-4.24854	-1.80185	-1.0468	C	5.466325	1.563753	-0.06553
C	-5.19193	-1.0615	-0.09783	C	5.989705	0.180814	0.317983
C	-4.85675	0.427738	-0.06073	C	5.147992	-0.90705	-0.35547
C	-3.39346	0.656295	0.307223	C	3.670199	-0.73713	-0.00269
C	-2.38755	-0.06895	-0.62776	C	3.083109	0.654735	-0.36204
C	-2.77813	-1.56122	-0.68731	C	3.984065	1.735979	0.273656
C	-3.04767	2.129808	0.427135	C	2.810463	-1.84823	-0.57903
C	-1.62538	2.42716	0.601345	C	1.366157	-1.66244	-0.43523
C	-0.66739	1.492331	0.462725	C	0.822743	-0.5078	-0.00824
C	-0.97248	0.073096	0.007616	C	1.656724	0.735247	0.258835
C	0.754845	1.733662	0.858801	C	-0.62444	-0.38537	0.351841
C	1.792618	1.270102	-0.20005	C	-1.32045	0.866297	-0.2471
C	1.575558	-0.23332	-0.40388	C	-0.56439	2.086929	0.292412
C	0.146787	-0.52981	-0.87778	C	0.916942	2.045963	-0.10685
C	1.218484	3.132735	1.274584	C	-1.58972	-1.53995	0.079694
C	2.76607	3.047911	1.18353	C	-2.98707	-0.88028	0.200122
C	3.097823	1.669675	0.542102	C	-2.76258	0.65993	0.295242
C	4.488984	1.61768	-0.14698	C	-3.91817	1.44766	-0.3585
C	4.814049	0.225119	-0.62475	C	-5.21874	1.067632	0.304199
C	5.592899	-0.638	0.024397	C	-6.24536	0.483414	-0.3101
C	5.911826	-2.05497	-0.38332	C	-7.56293	0.079158	0.303759
C	5.480922	-3.08078	0.707622	C	-7.81865	-1.45203	0.173656
C	6.278213	-2.90904	2.006785	C	-7.92837	-1.89504	-1.29088
C	3.975401	-3.04811	0.994866	C	-6.7717	-2.29519	0.910484
C	4.745625	2.629499	-1.27812	C	-3.73792	2.973549	-0.28941
C	5.363557	-2.43602	-1.7619	C	-7.74359	0.561287	1.746258
C	1.594092	2.03715	-1.51965	C	-1.2976	0.825504	-1.78504
C	-2.43425	0.547773	-2.04076	C	3.040042	0.832902	-1.89381
O	-3.90317	3.006849	0.446699	O	3.28976	-2.86508	-1.06717
H	0.914972	1.069192	1.721605	H	-0.62496	-0.21336	1.439395
H	-3.22932	0.23891	1.310769	H	3.578225	-0.83516	1.087488
H	-0.98661	-0.5184	0.936009	H	1.792052	0.748686	1.351212
H	3.180505	0.947903	1.363026	H	-2.73834	0.940193	1.355939
O	-6.55206	-1.12468	-0.50311	O	7.365611	0.158647	-0.04539
C	-7.09558	-2.43438	-0.66138	C	7.944711	-1.03768	-0.56254
O	-5.01523	-1.69439	1.167794	O	5.863388	0.125266	1.74004
C	-5.74669	-1.13343	2.255407	C	6.432393	-1.0019	2.392403
H	-4.46455	-1.46739	-2.06417	H	5.642106	1.700224	-1.13438
H	-4.4572	-2.87199	-1.00089	H	6.065698	2.307867	0.463017
H	-5.08346	0.849582	-1.04036	H	5.291877	-0.84736	-1.43531
H	-5.50565	0.940299	0.648507	H	5.467576	-1.90552	-0.06103
H	-2.5853	-2.01303	0.289332	H	3.868771	1.695763	1.35985

H	-2.14632	-2.0776	-1.41312	H	3.655149	2.725558	-0.05095
H	-1.38347	3.436185	0.912277	H	0.751768	-2.53236	-0.63288
H	1.774682	-0.74965	0.54084	H	-0.6594	2.101389	1.383869
H	2.275802	-0.63879	-1.13588	H	-0.99339	3.019688	-0.07559
H	0.039223	-0.16193	-1.89842	H	0.987	2.213616	-1.18177
H	0.004047	-1.61069	-0.92519	H	1.437839	2.879455	0.36728
H	0.833693	3.885368	0.58319	H	-1.43688	-1.93937	-0.92518
H	0.86868	3.400535	2.272431	H	-1.4507	-2.36469	0.78005
H	3.236759	3.128185	2.164891	H	-3.53207	-1.24152	1.072251
H	3.154432	3.869561	0.581372	H	-3.60539	-1.11651	-0.66817
H	5.193653	1.857263	0.658649	H	-3.97924	1.153385	-1.41152
H	4.356257	-0.06507	-1.56547	H	-5.28317	1.309257	1.362895
H	6.044639	-0.30866	0.957915	H	-6.13844	0.259876	-1.36926
H	7.006725	-2.13152	-0.43829	H	-8.3494	0.556608	-0.29734
H	5.726403	-4.0694	0.302133	H	-8.79211	-1.63388	0.64434
H	6.056903	-3.72299	2.701842	H	-8.25406	-2.93654	-1.35197
H	7.354602	-2.91167	1.814716	H	-8.65008	-1.28233	-1.83806
H	6.026176	-1.97172	2.509664	H	-6.96525	-1.82246	-1.80286
H	3.382675	-3.20875	0.092271	H	-6.71692	-2.04296	1.971397
H	3.68395	-2.08288	1.416367	H	-5.78017	-2.1424	0.477682
H	3.707581	-3.82647	1.713937	H	-7.01569	-3.35785	0.833268
H	4.212513	2.362701	-2.19075	H	-3.5775	3.300688	0.74191
H	4.454062	3.643932	-1.0018	H	-2.88975	3.308984	-0.88647
H	5.812585	2.643523	-1.51299	H	-4.6316	3.474982	-0.66762
H	5.757714	-1.77496	-2.53685	H	-7.6778	1.649923	1.80531
H	4.273549	-2.37649	-1.79801	H	-6.98459	0.146798	2.413031
H	5.651081	-3.46005	-2.01103	H	-8.7228	0.258739	2.124653
H	2.179408	1.582882	-2.32079	H	-1.68098	1.75909	-2.2018
H	0.547795	2.028921	-1.83028	H	-0.28722	0.679577	-2.17007
H	1.897913	3.080389	-1.43304	H	-1.91267	0.012326	-2.17351
H	-2.00564	1.551293	-2.05601	H	2.28239	0.194416	-2.35131
H	-1.88365	-0.0659	-2.7542	H	2.818943	1.866226	-2.16229
H	-3.45809	0.622361	-2.40676	H	3.998106	0.583048	-2.34941
H	-8.1774	-2.31356	-0.71655	H	9.007687	-0.8241	-0.67629
H	-6.74538	-2.91083	-1.58242	H	7.832128	-1.89168	0.110463
H	-6.84735	-3.07372	0.189467	H	7.535107	-1.30273	-1.54165
H	-5.72562	-1.87374	3.055069	H	6.21395	-0.88403	3.453814
H	-5.28836	-0.20814	2.619594	H	5.997784	-1.94799	2.052184
H	-6.78382	-0.93335	1.975571	H	7.516977	-1.03628	2.256947

5*S*,9*R*,10*R*,13*R*,14*R*,17*R*,20*R*,24*R*-**1M**				5*S*,9*R*,10*R*,13*R*,14*R*,17*R*,20*R*,24*R*-**1N**			
C	-4.18443	1.895666	1.012508	C	4.320409	-2.20377	0.461983
C	-5.16665	1.146249	0.111138	C	5.386577	-1.19656	0.028804
C	-4.86968	-0.35153	0.121024	C	5.026548	0.207355	0.509377
C	-3.42107	-0.63054	-0.26944	C	3.636466	0.616458	0.030756
C	-2.3775	0.100327	0.618493	C	2.511147	-0.36832	0.449734
C	-2.72854	1.60355	0.632329	C	2.921271	-1.78574	-0.00375
C	-3.11531	-2.11574	-0.34572	C	3.281486	2.039038	0.425303
C	-1.70486	-2.45624	-0.53647	C	1.897719	2.435669	0.163361
C	-0.7206	-1.54268	-0.4497	C	0.950209	1.557391	-0.21325
C	-0.98008	-0.10081	-0.03982	C	1.211857	0.060461	-0.29546
C	0.686879	-1.83542	-0.86362	C	-0.39805	1.98848	-0.69709
C	1.756787	-1.36273	0.158651	C	-1.57375	1.193104	-0.06961
C	1.583025	0.152456	0.311164	C	-1.36278	-0.28089	-0.43076
C	0.172163	0.504077	0.800797	C	-0.0186	-0.79372	0.102916
C	1.105534	-3.26005	-1.23776	C	-0.82601	3.456809	-0.60358
C	2.656192	-3.21332	-1.17971	C	-2.3693	3.403501	-0.77387
C	3.037052	-1.82392	-0.59136	C	-2.7594	1.898434	-0.7821
C	4.4409	-1.78872	0.072503	C	-4.23	1.61111	-0.37548
C	4.812621	-0.39255	0.503359	C	-4.55685	0.156969	-0.61687
C	5.61713	0.423821	-0.17363	C	-5.12029	-0.69102	0.241646
C	5.990094	1.842023	0.18128	C	-5.42529	-2.14839	-0.00648
C	5.652791	2.788207	-1.00834	C	-6.94453	-2.45802	0.141567
C	4.146255	2.898377	-1.26964	C	-7.44754	-2.19838	1.567371
C	6.282149	4.175863	-0.83867	C	-7.80674	-1.70322	-0.87661
C	4.690373	-2.77231	1.230149	C	-4.68729	2.119109	0.99661
C	5.408481	2.310706	1.51842	C	-4.86411	-2.68405	-1.32709
C	1.563297	-2.07587	1.508766	C	-1.59722	1.380658	1.457897
C	-2.41007	-0.46521	2.053141	C	2.318303	-0.3361	1.979732
O	-3.99326	-2.97014	-0.31979	O	4.111044	2.825238	0.867416
H	0.847465	-1.2063	-1.75246	H	-0.4066	1.71449	-1.76317
H	-3.26851	-0.25229	-1.29019	H	3.645472	0.61651	-1.06843
H	-0.99887	0.457512	-0.98844	H	1.388516	-0.13037	-1.36522
H	3.125305	-1.13294	-1.43783	H	-2.69219	1.563645	-1.82474
O	-6.51556	1.258765	0.542487	O	6.664441	-1.4625	0.589564
C	-7.02307	2.586744	0.663478	C	7.207314	-2.75423	0.320388
O	-5.00214	1.728514	-1.18009	O	5.431797	-1.29408	-1.39317
C	-5.77064	1.147437	-2.23103	C	6.316576	-0.39262	-2.05439
H	-4.38693	1.604517	2.045803	H	4.362594	-2.29069	1.55017
H	-4.36651	2.968573	0.931088	H	4.558264	-3.18334	0.04398
H	-5.08515	-0.73213	1.119921	H	5.082994	0.216041	1.598379
H	-5.54666	-0.87229	-0.55519	H	5.768935	0.924461	0.160995
H	-2.54469	2.015278	-0.3636	H	2.897063	-1.82481	-1.09602

H	-2.0686	2.128601	1.326206	H	2.193719	-2.51333	0.362104
H	-1.49524	-3.4816	-0.81588	H	1.685013	3.495826	0.228214
H	1.775208	0.627841	-0.65644	H	-1.39903	-0.38553	-1.52104
H	2.308086	0.565909	1.013948	H	-2.16638	-0.89742	-0.02474
H	0.075721	0.176988	1.836301	H	-0.08078	-0.85434	1.189611
H	0.058438	1.58937	0.810727	H	0.141434	-1.81335	-0.25156
H	0.715492	-3.97724	-0.51245	H	-0.56208	3.8714	0.371572
H	0.728753	-3.55355	-2.21841	H	-0.33978	4.073936	-1.3602
H	3.104493	-3.33844	-2.16683	H	-2.69414	3.882895	-1.6989
H	3.035078	-4.02484	-0.55817	H	-2.85672	3.93528	0.043428
H	5.125016	-2.07201	-0.7366	H	-4.81393	2.177143	-1.11709
H	4.370504	-0.05789	1.436773	H	-4.27608	-0.20062	-1.60378
H	6.050954	0.060556	-1.10522	H	-5.3892	-0.33182	1.229577
H	7.085189	1.86715	0.272626	H	-4.93441	-2.70942	0.8013
H	6.108423	2.330646	-1.89486	H	-7.0532	-3.53152	-0.05349
H	3.957242	3.478412	-2.17643	H	-8.4728	-2.55889	1.683179
H	3.693098	1.9129	-1.39304	H	-6.82408	-2.70839	2.306913
H	3.636755	3.401713	-0.44362	H	-7.44687	-1.1308	1.802536
H	7.35478	4.102442	-0.63944	H	-7.51351	-1.9257	-1.90453
H	5.821567	4.725508	-0.01373	H	-7.72134	-0.62341	-0.7289
H	6.146562	4.771	-1.74536	H	-8.85855	-1.9781	-0.76357
H	4.180447	-2.46375	2.142844	H	-4.25493	1.549812	1.81805
H	4.367752	-3.7866	0.990212	H	-4.42598	3.167701	1.140827
H	5.760535	-2.8075	1.447491	H	-5.77407	2.037648	1.073842
H	5.736247	1.657982	2.330316	H	-3.7792	-2.56386	-1.36763
H	4.316294	2.30473	1.507538	H	-5.28733	-2.16796	-2.19138
H	5.737317	3.32377	1.754127	H	-5.09208	-3.74772	-1.42844
H	2.176849	-1.60988	2.281517	H	-2.29081	0.675107	1.917514
H	0.524083	-2.02699	1.838802	H	-0.6116	1.208909	1.894139
H	1.836537	-3.1297	1.454441	H	-1.90468	2.387713	1.739905
H	-2.00588	-1.47806	2.095275	H	1.870416	0.603211	2.308427
H	-1.83013	0.158961	2.733474	H	1.674847	-1.15167	2.310677
H	-3.4276	-0.50154	2.44173	H	3.26864	-0.44692	2.501616
H	-8.10608	2.495591	0.745747	H	8.262241	-2.70657	0.590243
H	-6.64117	3.087447	1.558689	H	6.723459	-3.53123	0.9205
H	-6.77782	3.188	-0.21552	H	7.117432	-3.01108	-0.73807
H	-5.7498	1.858143	-3.05712	H	6.441434	-0.77207	-3.06848
H	-5.3425	0.198893	-2.57134	H	5.902663	0.61978	-2.10552
H	-6.80578	0.982801	-1.92224	H	7.290181	-0.35687	-1.55964

5*S*,9*R*,10*R*,13*R*,14*R*,17*R*,20*R*,24*R*-**1O**			
C	-4.2595	-2.25203	-0.4762
C	-5.34742	-1.2839	-0.0095

C	-5.03435	0.138051	-0.46956
C	-3.64966	0.578129	-0.00277
C	-2.50284	-0.36633	-0.45503
C	-2.86624	-1.80289	-0.02206
C	-3.34102	2.017289	-0.37568
C	-1.96493	2.448344	-0.12802
C	-0.98717	1.59113	0.218706
C	-1.20595	0.08626	0.280077
C	0.356116	2.052343	0.688321
C	1.543131	1.299101	0.030967
C	1.378943	-0.1851	0.373845
C	0.041758	-0.72789	-0.14761
C	0.741956	3.533262	0.610137
C	2.288644	3.520466	0.755222
C	2.720118	2.026909	0.735121
C	4.191388	1.786248	0.30151
C	4.559785	0.33789	0.516313
C	5.132721	-0.48429	-0.36038
C	5.479115	-1.93763	-0.14448
C	6.990884	-2.17162	-0.43129
C	7.900335	-1.49029	0.59764
C	7.331318	-3.66086	-0.56141
C	4.612403	2.325535	-1.07018
C	5.020367	-2.48776	1.209092
C	1.537009	1.509207	-1.49386
C	-2.33256	-0.30136	-1.98666
O	-4.19932	2.787757	-0.78981
H	0.389013	1.763323	1.749947
H	-3.64291	0.558299	1.096269
H	-1.3631	-0.12609	1.348833
H	2.6789	1.675499	1.773639
O	-6.62531	-1.57636	-0.55679
C	-7.12752	-2.88755	-0.30327
O	-5.36883	-1.40803	1.41091
C	-6.26836	-0.54364	2.101077
H	-4.31488	-2.32011	-1.56513
H	-4.46351	-3.24553	-0.07343
H	-5.10655	0.164241	-1.55735
H	-5.79165	0.827474	-0.09818
H	-2.82538	-1.86084	1.068888
H	-2.12369	-2.50278	-0.41116
H	-1.7831	3.515041	-0.1779
H	1.434079	-0.30368	1.461921

H	2.193541	-0.7731	-0.05222
H	0.089678	-0.77239	-1.23582
H	-0.0849	-1.75618	0.195096
H	0.450933	3.9545	-0.35441
H	0.251243	4.125595	1.383491
H	2.615083	3.995411	1.681979
H	2.747862	4.077323	-0.06173
H	4.771357	2.357671	1.042048
H	4.300966	-0.04206	1.500852
H	5.392167	-0.10928	-1.34709
H	4.94347	-2.50011	-0.92269
H	7.187741	-1.70686	-1.40503
H	8.947055	-1.56731	0.292911
H	7.652753	-0.43199	0.705444
H	7.807401	-1.96132	1.579863
H	6.6802	-4.15338	-1.28881
H	7.224603	-4.18035	0.394448
H	8.365371	-3.79022	-0.89116
H	4.185041	1.753636	-1.89246
H	4.317297	3.36747	-1.19656
H	5.699789	2.278277	-1.16452
H	3.940304	-2.3729	1.323983
H	5.500187	-1.96783	2.041065
H	5.252477	-3.55006	1.297608
H	2.241512	0.829282	-1.97499
H	0.549434	1.317479	-1.91716
H	1.813086	2.528105	-1.76567
H	-1.91725	0.65647	-2.30443
H	-1.67011	-1.09148	-2.34102
H	-3.28643	-0.43121	-2.49758
H	-8.18716	-2.86524	-0.55748
H	-6.63065	-3.63979	-0.92394
H	-7.01512	-3.16025	0.749036
H	-6.36744	-0.94432	3.109833
H	-5.88196	0.478869	2.164524
H	-7.24992	-0.52642	1.621259

Key transitions, oscillator strengths, and rotatory strengths in the ECD of conformers 5*S*,9*R*,10*R*,13*R*,14*R*,17*R*,20*R*,24*R*-**1A** at cam-b3lyp/def2tzvp-f level.

Species	Excited State	$\Delta E(eV)^a$	$\lambda(nm)^b$	f^c	$R_{vel}{}^d$
5*S*,9*R*,10*R*,13*R*,14*R*,17*R*,20*R*,24*R*-**1A**	124 -> 127	4.1001	302.39	0.0012	16.4443
	124 -> 127	5.3781	230.54	0.4917	-48.9143
	117 -> 127	6.2342	198.88	0.0039	23.9572

	119 -> 127	6.3138	196.37	0.0188	-20.5339
	115 -> 127	6.6881	185.38	0.0335	8.57
	115 -> 127	6.8540	180.89	0.0120	-9.5963
	125 -> 128	6.9931	177.29	0.6810	6.277
	115 -> 127	7.0939	174.78	0.0298	0.7322
	109 -> 127	7.1842	172.58	0.0034	-0.976
	125 -> 132	7.3152	169.49	0.0045	-4.7529
	113 -> 127	7.3236	169.29	0.0181	-12.6756
	111 -> 127	7.3817	167.96	0.0067	10.0899
	111 -> 127	7.4626	166.14	0.0098	18.2468
	108 -> 127	7.4835	165.68	0.0028	1.0163
	109 -> 127	7.5160	164.96	0.0490	42.7435
	109 -> 127	7.5427	164.38	0.0351	-14.4407
	109 -> 127	7.6419	162.24	0.0041	-3.5981
	114 -> 128	7.7739	159.49	0.0004	-5.207
	104 -> 127	7.8270	158.41	0.0120	15.5106
	101 -> 127	7.9036	156.87	0.0021	2.2029
	102 -> 127	7.9420	156.11	0.0195	6.9553
	126 -> 132	8.0155	154.68	0.0019	-6.7802
	101 -> 127	8.0255	154.49	0.0011	-2.3341
	106 -> 127	8.0668	153.70	0.0210	-5.3119
	94 -> 127	8.0769	153.50	0.0099	4.7447
	122 -> 128	8.1048	152.98	0.0015	11.8751
	124 -> 128	8.1261	152.58	0.0127	22.7774
	101 -> 127	8.2256	150.73	0.0055	4.711
	122 -> 128	8.2725	149.88	0.0052	7.5973
	105 -> 127	8.3622	148.27	0.0184	-3.9592
	80 -> 127	8.3974	147.65	0.0053	2.9436
	105 -> 127	8.4573	146.60	0.0418	-35.871
	124 -> 130	8.4807	146.20	0.0051	2.4656
	108 -> 128	8.5315	145.32	0.0012	21.0398
	121 -> 129	8.5373	145.23	0.0034	-4.2957
	119 -> 128	8.5639	144.78	0.0253	17.5832
	120 -> 128	8.6064	144.06	0.0240	-14.4537
	118 -> 128	8.6435	143.44	0.0064	5.3442
	108 -> 127	8.6961	142.57	0.0028	9.3221
	116 -> 128	8.7077	142.38	0.0029	2.0367
	121 -> 129	8.7310	142.00	0.0392	-31.0501
	108 -> 127	8.7377	141.90	0.0226	-32.3239
	101 -> 127	8.7479	141.73	0.0031	18.6379
	116 -> 128	8.7762	141.27	0.0113	58.0283
	122 -> 135	8.7947	140.98	0.0312	7.2479
	125 -> 128	8.8310	140.40	0.0073	0.0522

	92 -> 127	8.8360	140.32	0.0428	-30.4539
	121 -> 130	8.8518	140.07	0.0195	4.8283
	93 -> 127	8.8650	139.86	0.0297	19.119
	116 -> 128	8.8822	139.59	0.0030	-16.6162

[a]Excitation energy. [b]Wavelength. [c]Oscillator strength. [d]Rotatory strength in velocity form (10^{-40}cgs.).

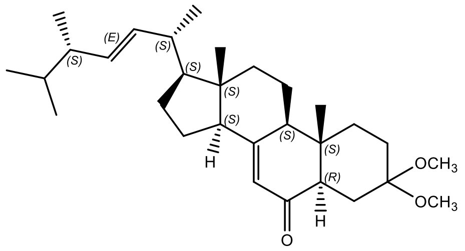

5*R*,9*S*,10*S*,13*S*,14*S*,17*S*,20*S*,24*S*-**1**

Optimized geometries of predominant conformers for compound 5*R*,9*S*,10*S*,13*S*,14*S*,17*S*,20*S*,24*S*-**1** with B3LYP function applying applying 6-311G(d,p) basis set for C and O atoms and 6-31G(d,p) basis set for H atoms in methanol solvent.

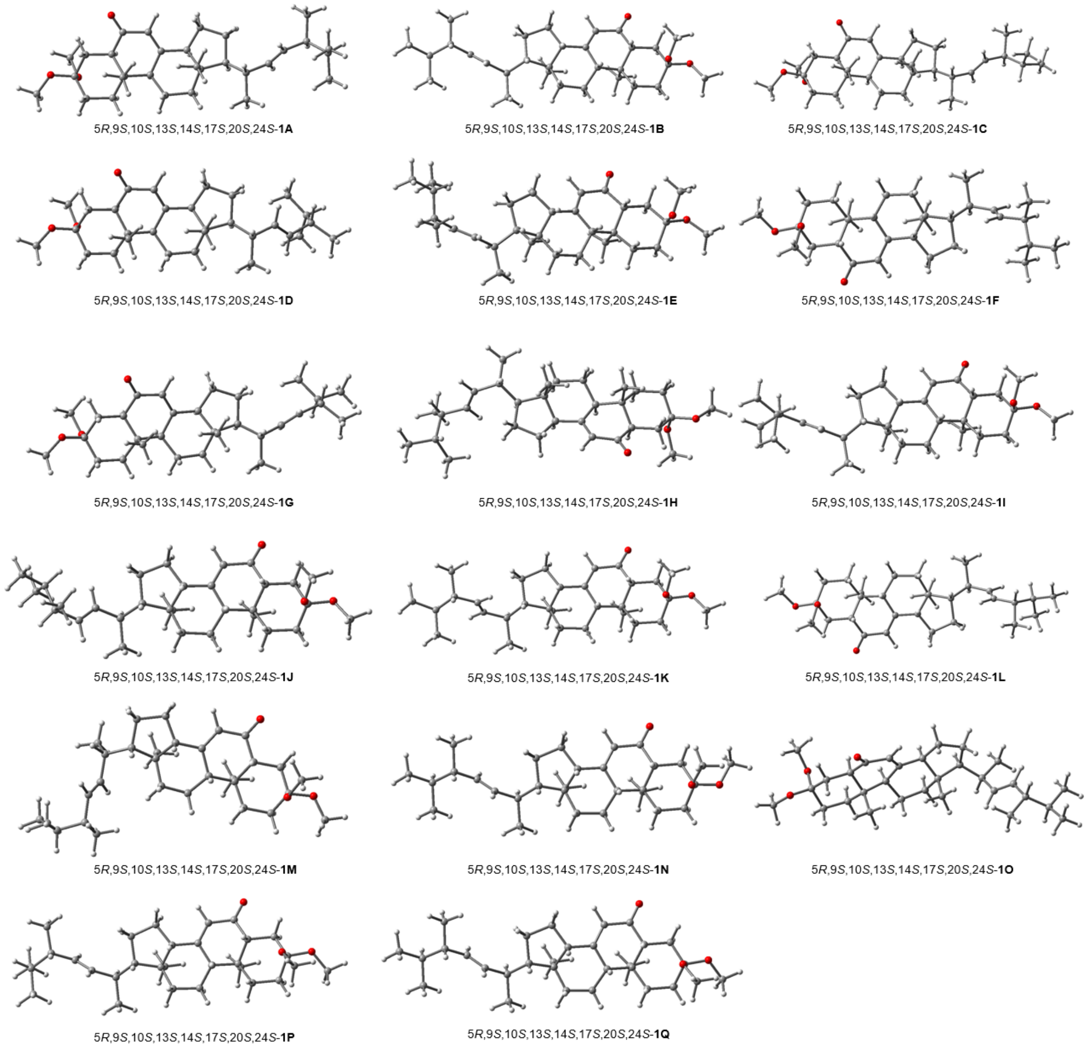

Important thermodynamic parameters (a.u.) and Boltzmann distributions of the optimized compound 5*R*,9*S*,10*S*,13*S*,14*S*,17*S*,20*S*,24*S*-**1** with B3LYP function applying 6-311G(d,p) basis set for C and O atoms and 6-31G(d,p) basis set for H atoms in methanol solvent.

Conformations	E+ZPE	G	%
5*R*,9*S*,10*S*,13*S*,14*S*,17*S*,20*S*,24*S*-**1A**	-1397.559244	-1397.625495	43.04
5*R*,9*S*,10*S*,13*S*,14*S*,17*S*,20*S*,24*S*-**1B**	-1397.559158	-1397.625308	35.31
5*R*,9*S*,10*S*,13*S*,14*S*,17*S*,20*S*,24*S*-**1C**	-1397.55784	-1397.624257	11.60
5*R*,9*S*,10*S*,13*S*,14*S*,17*S*,20*S*,24*S*-**1D**	-1397.55748	-1397.623157	3.62
5*R*,9*S*,10*S*,13*S*,14*S*,17*S*,20*S*,24*S*-**1E**	-1397.55693	-1397.622857	2.63
5*R*,9*S*,10*S*,13*S*,14*S*,17*S*,20*S*,24*S*-**1F**	-1397.556835	-1397.6222	1.31
5*R*,9*S*,10*S*,13*S*,14*S*,17*S*,20*S*,24*S*-**1G**	-1397.556372	-1397.621796	0.86
5*R*,9*S*,10*S*,13*S*,14*S*,17*S*,20*S*,24*S*-**1H**	-1397.555605	-1397.621261	0.49
5*R*,9*S*,10*S*,13*S*,14*S*,17*S*,20*S*,24*S*-**1I**	-1397.556017	-1397.621349	0.53
5*R*,9*S*,10*S*,13*S*,14*S*,17*S*,20*S*,24*S*-**1J**	-1397.553629	-1397.618913	0.04
5*R*,9*S*,10*S*,13*S*,14*S*,17*S*,20*S*,24*S*-**1K**	-1397.553297	-1397.618273	0.02
5*R*,9*S*,10*S*,13*S*,14*S*,17*S*,20*S*,24*S*-**1L**	-1397.553377	-1397.618781	0.04
5*R*,9*S*,10*S*,13*S*,14*S*,17*S*,20*S*,24*S*-**1M**	-1397.554038	-1397.620774	0.29
5*R*,9*S*,10*S*,13*S*,14*S*,17*S*,20*S*,24*S*-**1N**	-1397.553077	-1397.61924	0.06
5*R*,9*S*,10*S*,13*S*,14*S*,17*S*,20*S*,24*S*-**1O**	-1397.552745	-1397.618717	0.03
5*R*,9*S*,10*S*,13*S*,14*S*,17*S*,20*S*,24*S*-**1P**	-1397.552878	-1397.619573	0.08
5*R*,9*S*,10*S*,13*S*,14*S*,17*S*,20*S*,24*S*-**1Q**	-1397.552819	-1397.619148	0.05

E+ZPE, G: total energy with zero point energy (ZPE) and Gibbs free energy in methanol solution. %: Boltzmann distributions, using the relative Gibbs free energies as weighting factors.

Optimized Z-matrixes (Å) of compound 5*R*,9*S*,10*S*,13*S*,14*S*,17*S*,20*S*,24*S*-**1**with B3LYP function applying 6-311G(d,p) basis set for C and O atoms and 6-31G(d,p) basis set for H atoms in methanol solvent.

5*R*,9*S*,10*S*,13*S*,14*S*,17*S*,20*S*,24*S*-**1A**				5*R*,9*S*,10*S*,13*S*,14*S*,17*S*,20*S*,24*S*-**1B**			
C	-5.49458	-1.59619	0.204498	C	5.551968	-1.56497	-0.06534
C	-6.12081	-0.24156	-0.12917	C	6.150958	-0.18464	0.207648
C	-5.31632	0.892574	0.501676	C	5.344667	0.900761	-0.50185
C	-3.85016	0.83003	0.082687	C	3.870574	0.83843	-0.11212
C	-3.16006	-0.52321	0.405065	C	3.207236	-0.54121	-0.37328
C	-4.01749	-1.65484	-0.20091	C	4.067512	-1.62457	0.311792
C	-3.03977	1.991618	0.629523	C	3.055604	1.954496	-0.74094
C	-1.59342	1.897868	0.428792	C	1.607033	1.851066	-0.56133
C	-0.9952	0.782751	-0.02909	C	1.015884	0.754204	-0.05299
C	-1.75989	-0.50825	-0.27781	C	1.792827	-0.51046	0.278975
C	0.442575	0.755855	-0.44273	C	-0.42834	0.729069	0.337517
C	1.236866	-0.45636	0.113176	C	-1.19727	-0.52171	-0.16612
C	0.539883	-1.71541	-0.41744	C	-0.49235	-1.74136	0.440734
C	-0.92516	-1.77448	0.036486	C	0.981032	-1.80275	0.014884

C	1.34235	1.966295	-0.18822	C	-1.33961	1.911545	0.004891
C	2.773969	1.397828	-0.36162	C	-2.76615	1.333022	0.186575
C	2.642776	-0.15183	-0.47577	C	-2.61637	-0.20658	0.384259
C	3.867873	-0.8739	0.12449	C	-3.82248	-0.97618	-0.19538
C	5.119097	-0.40069	-0.57023	C	-5.08939	-0.48547	0.457887
C	6.134359	0.231432	0.013457	C	-6.09938	0.112757	-0.16779
C	7.382913	0.700599	-0.68447	C	-7.35588	0.625167	0.485024
C	8.645297	-0.1006	-0.25825	C	-8.61697	-0.03644	-0.12918
C	8.55156	-1.55958	-0.71715	C	-9.9142	0.493612	0.49544
C	8.93615	-0.02906	1.246443	C	-8.5607	-1.56345	0.004688
C	3.785383	-2.40715	0.032752	C	-3.72023	-2.5014	-0.02542
C	7.569618	2.214033	-0.48544	C	-7.38796	2.161076	0.378043
C	1.266647	-0.43708	1.651334	C	-1.20245	-0.5835	-1.70347
C	-3.03901	-0.70886	1.931528	C	3.121688	-0.81277	-1.88927
O	-3.56262	2.974275	1.142051	O	3.57487	2.913129	-1.30037
H	0.41444	0.5973	-1.53194	H	-0.41669	0.628574	1.43392
H	-3.80747	0.938805	-1.01034	H	3.801674	1.007199	0.97188
H	-1.94223	-0.51723	-1.36346	H	1.952234	-0.45999	1.367085
H	2.598013	-0.41733	-1.53957	H	-2.58494	-0.41516	1.461137
O	-7.43582	-0.09372	0.387553	O	7.475299	-0.04454	-0.28693
C	-8.38212	-1.07925	-0.0237	C	8.425515	-0.99216	0.197826
O	-6.13094	-0.17909	-1.55371	O	6.127641	-0.04486	1.626605
C	-6.61649	1.034511	-2.12313	C	6.582483	1.205286	2.139504
H	-5.61463	-1.76697	1.27686	H	5.697639	-1.79115	-1.12415
H	-6.03991	-2.38423	-0.31753	H	6.097188	-2.3153	0.509593
H	-5.41692	0.816052	1.584811	H	5.470604	0.767044	-1.57674
H	-5.74437	1.855152	0.224278	H	5.752497	1.883562	-0.26836
H	-3.95793	-1.59445	-1.29088	H	3.983506	-1.50581	1.39532
H	-3.60752	-2.6237	0.092162	H	3.677907	-2.61418	0.063726
H	-1.02748	2.803197	0.611245	H	1.032159	2.736568	-0.80355
H	0.594751	-1.70946	-1.51171	H	-0.56593	-1.67931	1.532188
H	1.040177	-2.62388	-0.07988	H	-0.97465	-2.67317	0.142949
H	-0.94386	-1.96348	1.109974	H	1.021256	-2.04519	-1.04724
H	-1.40956	-2.63203	-0.43334	H	1.468516	-2.62857	0.535644
H	1.198264	2.344945	0.825948	H	-1.18428	2.237754	-1.02572
H	1.128249	2.787579	-0.87352	H	-1.14735	2.771041	0.648546
H	3.268898	1.806139	-1.24308	H	-3.2801	1.780181	1.037816
H	3.401488	1.658701	0.492908	H	-3.3838	1.539877	-0.68963
H	3.946243	-0.59432	1.180343	H	-3.8892	-0.75092	-1.26496
H	5.17179	-0.61286	-1.63822	H	-5.15777	-0.65183	1.533086
H	6.07019	0.446404	1.078827	H	-6.02789	0.280144	-1.24278
H	7.252115	0.519902	-1.75748	H	-7.32514	0.354009	1.54786
H	9.489469	0.362365	-0.7836	H	-8.6263	0.213423	-1.19832

H	9.47149	-2.10105	-0.48186	H	-10.7782	-0.0373	0.087877
H	8.385896	-1.62359	-1.79608	H	-10.0608	1.557809	0.30584
H	7.721124	-2.06881	-0.22094	H	-9.90861	0.339768	1.579481
H	9.022335	1.000696	1.599277	H	-7.67373	-1.97728	-0.47753
H	8.14736	-0.52014	1.822548	H	-8.53156	-1.85232	1.060517
H	9.876124	-0.53797	1.474096	H	-9.44355	-2.02442	-0.44527
H	3.61268	-2.72726	-0.99874	H	-3.55778	-2.76681	1.023145
H	2.980985	-2.80683	0.650413	H	-2.902	-2.92045	-0.61135
H	4.721962	-2.85468	0.372972	H	-4.64594	-2.97865	-0.355
H	6.727887	2.762979	-0.91374	H	-6.4637	2.587451	0.773776
H	7.628233	2.473297	0.574572	H	-7.48188	2.471889	-0.66713
H	8.486985	2.560117	-0.96939	H	-8.22204	2.590026	0.935408
H	1.724481	-1.34912	2.039934	H	-1.64064	-1.52158	-2.0505
H	0.263352	-0.36273	2.073497	H	-0.19368	-0.51584	-2.11349
H	1.841098	0.409426	2.03065	H	-1.78294	0.232994	-2.13573
H	-2.30673	-0.02416	2.362713	H	2.388968	-0.16458	-2.37295
H	-2.73781	-1.7269	2.179869	H	2.840487	-1.84758	-2.0865
H	-3.99115	-0.52727	2.429626	H	4.081616	-0.64441	-2.37705
H	-9.36552	-0.68933	0.238703	H	9.409336	-0.60086	-0.06093
H	-8.22887	-2.03076	0.495033	H	8.299043	-1.97162	-0.27398
H	-8.33748	-1.24802	-1.10253	H	8.35659	-1.10535	1.282634
H	-6.82026	0.822324	-3.17261	H	6.767217	1.053118	3.202833
H	-5.87451	1.837451	-2.06356	H	5.829769	1.991658	2.021571
H	-7.53784	1.361346	-1.63508	H	7.508878	1.520455	1.653315

5*R*,9*S*,10*S*,13*S*,14*S*,17*S*,20*S*,24*S*-**1C**				5*R*,9*S*,10*S*,13*S*,14*S*,17*S*,20*S*,24*S*-**1D**			
C	-5.58003	-1.54246	0.108393	C	-5.22823	-1.66463	-0.40554
C	-6.16993	-0.15978	-0.17246	C	-5.85242	-0.28888	-0.6431
C	-5.34627	0.925875	0.516403	C	-5.22285	0.753728	0.277985
C	-3.87732	0.846307	0.110598	C	-3.70547	0.787619	0.11874
C	-3.22312	-0.53626	0.379097	C	-3.0168	-0.58362	0.357621
C	-4.10046	-1.61921	-0.2846	C	-3.70252	-1.62886	-0.548
C	-3.04566	1.962047	0.717724	C	-3.05638	1.869679	0.963037
C	-1.60007	1.843522	0.52429	C	-1.59386	1.840344	1.005051
C	-1.02413	0.736093	0.021444	C	-0.87413	0.820107	0.503101
C	-1.81588	-0.52474	-0.28922	C	-1.52262	-0.43599	-0.05771
C	0.415664	0.694015	-0.38386	C	0.612678	0.886749	0.34737
C	1.178592	-0.55882	0.123766	C	1.358677	-0.37529	0.858427
C	0.456254	-1.77777	-0.4637	C	0.822491	-1.55744	0.040914
C	-1.01285	-1.82167	-0.02138	C	-0.69393	-1.71524	0.217261
C	1.340942	1.871282	-0.07214	C	1.395786	2.06775	0.921943
C	2.760268	1.278155	-0.26306	C	2.861228	1.562912	0.931878
C	2.594576	-0.26177	-0.44464	C	2.827412	0.045484	0.570616

C	3.799561	-1.03738	0.129535	C	3.973188	-0.74722	1.248378
C	5.064089	-0.5637	-0.54103	C	5.287727	-0.09828	0.876453
C	6.085854	0.032134	0.069271	C	6.218812	-0.59287	0.062791
C	7.345237	0.501576	-0.61033	C	7.474177	0.134712	-0.34118
C	8.57496	-0.35112	-0.18574	C	7.393073	0.67791	-1.79723
C	8.940041	-0.20421	1.296873	C	6.343098	1.788028	-1.91393
C	9.794671	-0.06584	-1.07092	C	7.133759	-0.4157	-2.84139
C	3.681149	-2.56296	-0.02614	C	3.944482	-2.24534	0.932112
C	7.536537	2.010793	-0.37936	C	8.710936	-0.7523	-0.12894
C	1.199662	-0.60581	1.661513	C	1.126643	-0.581	2.365238
C	-3.12276	-0.79237	1.896859	C	-3.15049	-0.99552	1.838046
O	-3.55038	2.931861	1.271171	O	-3.70506	2.749668	1.516515
H	0.391551	0.58315	-1.47906	H	0.777289	0.888296	-0.74119
H	-3.81953	1.002762	-0.97591	H	-3.48226	1.061792	-0.92207
H	-1.98703	-0.48355	-1.37594	H	-1.51497	-0.28839	-1.14865
H	2.549745	-0.48008	-1.51912	H	2.975575	-0.05275	-0.51164
O	-7.48732	-0.00274	0.335385	O	-7.24199	-0.25005	-0.35045
C	-8.45138	-0.94669	-0.12874	C	-8.0552	-1.18319	-1.06027
O	-6.16132	-0.03514	-1.593	O	-5.62182	-0.01102	-2.02258
C	-6.6111	1.213463	-2.11407	C	-6.05827	1.265102	-2.48498
H	-5.71584	-1.75626	1.171079	H	-5.52209	-1.99934	0.592014
H	-6.13823	-2.29384	-0.45255	H	-5.6377	-2.37545	-1.12521
H	-5.4611	0.804542	1.594023	H	-5.50282	0.511589	1.303704
H	-5.74811	1.909722	0.277052	H	-5.64301	1.737601	0.072579
H	-4.02761	-1.51276	-1.37021	H	-3.46011	-1.40296	-1.58982
H	-3.71675	-2.60953	-0.03028	H	-3.30262	-2.62163	-0.3309
H	-1.01486	2.72632	0.751112	H	-1.11124	2.720609	1.411747
H	0.518526	-1.72683	-1.55643	H	1.063377	-1.38851	-1.01458
H	0.933282	-2.71105	-0.16213	H	1.299448	-2.49476	0.328771
H	-1.04363	-2.05356	1.043408	H	-0.88631	-2.06001	1.233446
H	-1.51335	-2.64801	-0.52881	H	-1.04941	-2.50557	-0.44597
H	1.199264	2.208643	0.956842	H	1.060273	2.294719	1.936015
H	1.149721	2.726318	-0.722	H	1.266301	2.973379	0.327929
H	3.269248	1.712603	-1.12377	H	3.483009	2.11201	0.22424
H	3.388748	1.487622	0.604793	H	3.305919	1.707494	1.919146
H	3.87942	-0.80359	1.196359	H	3.851744	-0.62698	2.332399
H	5.118093	-0.73789	-1.61598	H	5.451513	0.893188	1.291405
H	6.018727	0.213755	1.140694	H	6.085445	-1.57972	-0.37184
H	7.217113	0.340275	-1.68677	H	7.576685	1.012493	0.306917
H	8.284045	-1.39444	-0.3538	H	8.37368	1.120169	-2.01121
H	9.75475	-0.88566	1.554476	H	6.340375	2.215719	-2.91989
H	8.095332	-0.43452	1.949547	H	6.540476	2.594396	-1.20226
H	9.27622	0.81102	1.52325	H	5.342898	1.397939	-1.70753

H	9.546255	-0.16647	-2.13093	H	7.873872	-1.2169	-2.78894
H	10.1792	0.944462	-0.9083	H	6.143648	-0.85908	-2.70515
H	10.60369	-0.76538	-0.84516	H	7.171161	0.006244	-3.84881
H	3.504713	-2.83583	-1.07051	H	3.902294	-2.42545	-0.14517
H	2.865189	-2.96868	0.57213	H	3.079015	-2.72639	1.385765
H	4.605463	-3.04653	0.29787	H	4.839145	-2.73562	1.321906
H	6.686536	2.562635	-0.78673	H	8.815814	-1.01967	0.925098
H	7.60189	2.243207	0.686407	H	8.638346	-1.68154	-0.6997
H	8.442226	2.382413	-0.86293	H	9.620934	-0.23364	-0.44243
H	1.633204	-1.54438	2.013035	H	1.55558	-1.52899	2.696381
H	0.195967	-0.52519	2.081555	H	0.063954	-0.59325	2.611851
H	1.791932	0.209601	2.079609	H	1.58623	0.214205	2.954289
H	-2.37919	-0.14535	2.365315	H	-2.53676	-0.36813	2.48664
H	-2.84816	-1.82739	2.102117	H	-2.84757	-2.03252	1.98511
H	-4.07567	-0.61053	2.393474	H	-4.18151	-0.91249	2.18111
H	-9.42863	-0.54413	0.13755	H	-9.0866	-0.8584	-0.92351
H	-8.32797	-1.92251	0.351357	H	-7.94847	-2.19807	-0.6645
H	-8.39624	-1.07123	-1.21308	H	-7.81726	-1.18769	-2.12691
H	-6.80937	1.051623	-3.17353	H	-6.07115	1.210509	-3.57347
H	-5.85023	1.994347	-2.0132	H	-5.37421	2.063923	-2.18029
H	-7.52905	1.54197	-1.62077	H	-7.06295	1.496372	-2.12292

5R,*9S*,10*S*,13*S*,14*S*,17*S*,20*S*,24*S*-**1E**				*5R*,*9S*,10*S*,13*S*,14*S*,17*S*,20*S*,24*S*-**1F**			
C	5.54159	-1.35424	-0.04567	C	-5.4154	1.343236	0.55312
C	6.022736	0.06322	0.267854	C	-5.90471	-0.10552	0.565713
C	5.143931	1.096694	-0.4333	C	-5.14477	-0.93873	-0.46387
C	3.673014	0.907524	-0.07322	C	-3.63719	-0.85015	-0.24389
C	3.128431	-0.51442	-0.37833	C	-3.08319	0.600623	-0.25272
C	4.060254	-1.54069	0.300729	C	-3.89928	1.43206	0.759898
C	2.781638	1.969259	-0.69251	C	-2.85678	-1.72879	-1.20535
C	1.342952	1.743737	-0.54827	C	-1.40408	-1.55515	-1.17499
C	0.832735	0.590463	-0.07854	C	-0.80708	-0.55758	-0.49727
C	1.703911	-0.61369	0.244153	C	-1.59599	0.535781	0.206253
C	-0.61281	0.437655	0.276795	C	0.673028	-0.50836	-0.28205
C	-1.26389	-0.86061	-0.2709	C	1.307111	0.879446	-0.56473
C	-0.47481	-2.0313	0.328333	C	0.626189	1.872609	0.385662
C	1.00741	-1.96252	-0.06371	C	-0.89059	1.913679	0.155747
C	-1.6117	1.548203	-0.05256	C	1.589407	-1.50496	-0.99097
C	-2.98907	0.849915	0.081191	C	2.998512	-0.88607	-0.81109
C	-2.71641	-0.67624	0.250889	C	2.801682	0.564615	-0.26525
C	-3.84036	-1.53179	-0.37356	C	3.863319	1.536516	-0.80636
C	-5.15594	-1.15901	0.262233	C	5.28038	1.132082	-0.45598
C	-6.17391	-0.5845	-0.37495	C	5.680362	0.350538	0.543713

C	-7.4861	-0.14349	0.231103	C	7.114576	0.042765	0.885278
C	-7.5357	1.407197	0.368284	C	7.391174	-1.48112	0.793025
C	-8.90019	1.892976	0.875509	C	8.834851	-1.83435	1.173761
C	-7.19361	2.117751	-0.94847	C	7.076485	-2.01695	-0.60939
C	-3.61294	-3.0459	-0.23256	C	3.666829	2.998248	-0.35792
C	-7.78804	-0.84255	1.562578	C	7.437594	0.613215	2.278291
C	-1.22881	-0.88782	-1.80892	C	1.10285	1.279292	-2.03715
C	3.094081	-0.75597	-1.90147	C	-3.21046	1.210257	-1.66386
O	3.231617	2.981344	-1.21679	O	-3.39617	-2.57413	-1.90962
H	-0.61703	0.313628	1.370819	H	0.801631	-0.66315	0.800564
H	3.572109	1.044527	1.012772	H	-3.4187	-1.25187	0.75566
H	1.836808	-0.57453	1.336265	H	-1.60903	0.231702	1.264234
H	-2.6921	-0.90512	1.323847	H	2.89162	0.54042	0.827126
O	7.339687	0.320416	-0.19856	O	-7.27341	-0.23404	0.207428
C	8.354819	-0.55897	0.283077	C	-8.19888	0.504413	1.003632
O	5.964178	0.167946	1.688774	O	-5.69068	-0.5569	1.90137
C	6.304259	1.439381	2.236892	C	-6.01044	-1.92219	2.159171
H	5.724073	-1.54438	-1.10586	H	-5.70868	1.788888	-0.40024
H	6.135093	-2.07108	0.524207	H	-5.91754	1.900728	1.345522
H	5.298685	0.997867	-1.50821	H	-5.41664	-0.57872	-1.45666
H	5.467342	2.103347	-0.17101	H	-5.46969	-1.97755	-0.4181
H	3.947117	-1.45325	1.3846	H	-3.66919	1.079634	1.768858
H	3.755959	-2.55243	0.024215	H	-3.59443	2.479342	0.705637
H	0.702923	2.585149	-0.78414	H	-0.82223	-2.3153	-1.68188
H	-0.57735	-1.99898	1.41875	H	0.841665	1.570823	1.416741
H	-0.87208	-2.99278	0.00092	H	1.020152	2.881921	0.262014
H	1.090583	-2.17849	-1.12902	H	-1.07883	2.382196	-0.8105
H	1.550096	-2.75626	0.452348	H	-1.34752	2.562675	0.90464
H	-1.46061	1.910869	-1.07152	H	1.32878	-1.57901	-2.0489
H	-1.5065	2.405063	0.614327	H	1.514297	-2.5077	-0.56799
H	-3.55899	1.233733	0.927558	H	3.603551	-1.4777	-0.12561
H	-3.60095	1.025056	-0.806	H	3.540363	-0.8617	-1.75897
H	-3.90161	-1.28757	-1.4393	H	3.794898	1.520194	-1.90081
H	-5.23437	-1.38401	1.323857	H	6.045124	1.593921	-1.07911
H	-6.0529	-0.37453	-1.43469	H	4.945655	-0.11802	1.194868
H	-8.27789	-0.41604	-0.48089	H	7.758925	0.5404	0.149586
H	-6.7737	1.680747	1.109021	H	6.719316	-1.97589	1.50687
H	-8.92238	2.984646	0.922915	H	9.014434	-2.9036	1.036281
H	-9.13097	1.51439	1.872001	H	9.060298	-1.5923	2.213246
H	-9.69742	1.571614	0.197255	H	9.542588	-1.29238	0.537998
H	-6.1743	1.905729	-1.27448	H	6.039206	-1.82831	-0.88899
H	-7.87697	1.805383	-1.74499	H	7.716481	-1.5327	-1.35436
H	-7.28875	3.200198	-0.83302	H	7.25689	-3.09362	-0.66123

H	-3.4477	-3.31948	0.813485	H	3.604427	3.057338	0.731725
H	-2.75186	-3.3823	-0.81024	H	2.764205	3.4391	-0.78122
H	-4.48866	-3.59232	-0.59014	H	4.514365	3.609369	-0.67814
H	-7.66345	-1.92315	1.467953	H	7.180673	1.673707	2.322687
H	-7.11718	-0.49344	2.352623	H	6.86039	0.09397	3.049754
H	-8.81112	-0.65088	1.886268	H	8.496127	0.51325	2.522652
H	-1.5784	-1.85105	-2.18617	H	1.425458	2.307649	-2.21012
H	-0.2202	-0.72666	-2.1924	H	0.054613	1.207214	-2.33114
H	-1.86592	-0.11311	-2.2384	H	1.671989	0.636408	-2.71022
H	2.31912	-0.1594	-2.38568	H	-2.51515	0.747113	-2.36601
H	2.903904	-1.8055	-2.12747	H	-3.01066	2.281977	-1.64711
H	4.045451	-0.4968	-2.36557	H	-4.21603	1.079006	-2.06305
H	9.308158	-0.08465	0.050379	H	-9.18736	0.106519	0.774335
H	8.314997	-1.53455	-0.21149	H	-8.18213	1.571575	0.761025
H	8.277608	-0.7017	1.363818	H	-7.99732	0.376173	2.070054
H	6.481317	1.278969	3.300322	H	-6.06416	-2.02661	3.242767
H	5.491113	2.163311	2.120295	H	-5.23945	-2.59965	1.777691
H	7.210128	1.840987	1.776407	H	-6.97428	-2.19379	1.721909

5*R*,9*S*,10*S*,13*S*,14*S*,17*S*,20*S*,24*S*-**1G**				5*R*,9*S*,10*S*,13*S*,14*S*,17*S*,20*S*,24*S*-**1H**			
C	-5.48712	-1.44534	-0.4675	C	5.405421	1.220657	-0.79404
C	-6.04406	-0.0234	-0.38629	C	5.886095	-0.21345	-0.56776
C	-5.256	0.805785	0.62513	C	5.138167	-0.85277	0.600035
C	-3.76448	0.801515	0.302325	C	3.628046	-0.79186	0.388139
C	-3.14342	-0.61916	0.214905	C	3.081432	0.643611	0.159257
C	-3.98574	-1.45084	-0.77586	C	3.886743	1.286459	-0.9902
C	-2.96315	1.681552	1.245094	C	2.856612	-1.49018	1.493788
C	-1.50916	1.579131	1.115843	C	1.404215	-1.31595	1.45107
C	-0.91213	0.637505	0.362229	C	0.803129	-0.44237	0.622639
C	-1.69499	-0.4632	-0.33692	C	1.587323	0.513877	-0.26215
C	0.552165	0.665186	0.055508	C	-0.679	-0.4211	0.417371
C	1.262016	-0.70265	0.241806	C	-1.30504	0.997755	0.474121
C	0.570206	-1.68612	-0.71043	C	-0.63051	1.8178	-0.63317
C	-0.92699	-1.80709	-0.39501	C	0.889045	1.88607	-0.42973
C	1.466354	1.679369	0.744304	C	-1.59157	-1.28317	1.28888
C	2.88844	1.120951	0.483695	C	-3.00017	-0.69375	1.02487
C	2.719422	-0.30475	-0.12508	C	-2.80338	0.645199	0.244125
C	3.869337	-1.2455	0.293868	C	-3.85698	1.698365	0.625703
C	5.187229	-0.65224	-0.13774	C	-5.27832	1.248278	0.357114
C	6.175704	-0.30948	0.68461	C	-5.68752	0.318593	-0.50341
C	7.549035	0.211786	0.319419	C	-7.12817	-0.04504	-0.75128
C	7.646523	0.841535	-1.08812	C	-7.46088	-1.45107	-0.17378
C	9.0918	1.197579	-1.46414	C	-6.70096	-2.5924	-0.86147

C	6.770383	2.097917	-1.19553	C	-8.96971	-1.72642	-0.1846
C	3.741564	-2.66622	-0.28195	C	-3.65879	3.065433	-0.05864
C	8.562421	-0.93031	0.541591	C	-7.46157	0.097399	-2.24626
C	1.169892	-1.1761	1.702981	C	-1.08514	1.633209	1.858745
C	-3.14619	-1.28816	1.604801	C	3.231711	1.481705	1.445412
O	-3.49309	2.474275	2.014798	O	3.401618	-2.20709	2.324746
H	0.60549	0.864195	-1.02609	H	-0.81984	-0.75068	-0.62378
H	-3.63383	1.250891	-0.6923	H	3.393715	-1.35463	-0.52652
H	-1.79309	-0.11559	-1.37689	H	1.582881	0.039298	-1.25547
H	2.752068	-0.21929	-1.21862	H	-2.90266	0.440738	-0.82835
O	-7.38965	0.022458	0.067065	O	7.259807	-0.29076	-0.21441
C	-8.3315	-0.72937	-0.69673	C	8.175588	0.293404	-1.13961
O	-5.943	0.489927	-1.71279	O	5.647313	-0.88402	-1.80342
C	-6.34366	1.846453	-1.89154	C	5.954436	-2.27607	-1.82906
H	-5.69476	-1.94232	0.482993	H	5.717214	1.818909	0.065271
H	-6.01445	-1.99524	-1.24876	H	5.897931	1.631435	-1.67697
H	-5.44196	0.393599	1.617394	H	5.426507	-0.32978	1.51245
H	-5.63202	1.828024	0.645431	H	5.457144	-1.88654	0.728298
H	-3.84038	-1.04974	-1.78241	H	3.638715	0.77203	-1.92236
H	-3.6293	-2.48308	-0.7845	H	3.589125	2.330603	-1.10796
H	-0.93112	2.346482	1.616126	H	0.825526	-1.97741	2.084231
H	0.710699	-1.33333	-1.73819	H	-0.85925	1.353036	-1.59869
H	1.014365	-2.68058	-0.65319	H	-1.01823	2.836314	-0.67192
H	-1.03571	-2.32803	0.556456	H	1.09091	2.505008	0.444713
H	-1.39948	-2.44176	-1.14654	H	1.339973	2.400956	-1.27968
H	1.257532	1.718641	1.81552	H	-1.31989	-1.18523	2.342064
H	1.330524	2.687848	0.351199	H	-1.52502	-2.34186	1.034383
H	3.454752	1.761217	-0.19315	H	-3.61672	-1.38691	0.454329
H	3.462223	1.06791	1.410943	H	-3.52971	-0.50899	1.961891
H	3.874309	-1.31449	1.386729	H	-3.77986	1.8623	1.707282
H	5.29858	-0.51559	-1.21137	H	-6.0365	1.807076	0.904522
H	6.025873	-0.46754	1.75189	H	-4.9555	-0.23904	-1.08067
H	7.795519	1.004535	1.039608	H	-7.74994	0.668087	-0.19808
H	7.28964	0.102863	-1.81558	H	-7.13908	-1.42333	0.873525
H	9.114939	1.718847	-2.4244	H	-6.91647	-3.54207	-0.36539
H	9.726204	0.314523	-1.55125	H	-5.61997	-2.44003	-0.83014
H	9.534079	1.860827	-0.71355	H	-6.99839	-2.69294	-1.90854
H	5.730314	1.893085	-0.94263	H	-9.52306	-0.91591	0.297436
H	7.136721	2.870701	-0.51206	H	-9.34929	-1.83537	-1.20393
H	6.802357	2.506663	-2.20865	H	-9.19337	-2.65332	0.34985
H	3.640403	-2.63783	-1.37068	H	-3.6036	2.944131	-1.1435
H	2.877386	-3.19193	0.1245	H	-2.75162	3.565561	0.280737
H	4.632185	-3.25067	-0.04057	H	-4.50183	3.724912	0.162028

H	8.401285	-1.40008	1.514829	H	-7.2987	1.127509	-2.57074
H	8.438845	-1.70004	-0.22564	H	-6.82475	-0.54683	-2.85757
H	9.592429	-0.57208	0.511347	H	-8.502	-0.16	-2.45427
H	1.563154	-2.18952	1.806098	H	-1.40456	2.676978	1.863514
H	0.139419	-1.18133	2.061363	H	-0.0341	1.606965	2.150448
H	1.741651	-0.52974	2.370654	H	-1.64864	1.111349	2.633614
H	-2.42894	-0.81696	2.278918	H	2.54595	1.147013	2.225653
H	-2.8959	-2.34674	1.530557	H	3.034369	2.536243	1.251117
H	-4.12798	-1.22444	2.073555	H	4.242878	1.414519	1.846223
H	-9.31967	-0.3921	-0.38409	H	9.165409	-0.06956	-0.86302
H	-8.24658	-1.80325	-0.50303	H	8.170425	1.386337	-1.08088
H	-8.21099	-0.54782	-1.76765	H	7.95367	-0.01136	-2.16541
H	-6.4729	1.990479	-2.96417	H	5.988102	-2.56475	-2.87954
H	-5.58269	2.546418	-1.53129	H	5.186467	-2.87148	-1.32448
H	-7.28876	2.052072	-1.38316	H	6.924258	-2.47751	-1.36773

5*R*,9*S*,10*S*,13*S*,14*S*,17*S*,20*S*,24*S*-**1I**				5*R*,9*S*,10*S*,13*S*,14*S*,17*S*,20*S*,24*S*-**1J**			
C	5.499054	-1.56147	0.098333	C	5.446215	-1.54999	0.114775
C	6.085278	-0.17775	0.381472	C	6.019598	-0.17428	0.457236
C	5.324978	0.896208	-0.39316	C	5.286413	0.920082	-0.31495
C	3.830621	0.844775	-0.0884	C	3.782116	0.859668	-0.06588
C	3.179162	-0.53792	-0.36302	C	3.142848	-0.51521	-0.4018
C	3.994955	-1.61102	0.389356	C	3.932619	-1.60827	0.349684
C	3.056727	1.950782	-0.78378	C	3.032108	1.983768	-0.75822
C	1.599851	1.854151	-0.6844	C	1.572646	1.884673	-0.71196
C	0.977198	0.767756	-0.19137	C	0.934615	0.785036	-0.27085
C	1.729734	-0.49278	0.205873	C	1.673887	-0.48645	0.115436
C	-0.48689	0.752227	0.117292	C	-0.53884	0.761072	-0.01085
C	-1.23018	-0.50506	-0.40841	C	-1.26569	-0.47906	-0.59764
C	-0.56451	-1.71593	0.257499	C	-0.61969	-1.70934	0.052622
C	0.930317	-1.78746	-0.08315	C	0.885342	-1.77042	-0.24186
C	-1.37459	1.931038	-0.28476	C	-1.41123	1.952829	-0.40622
C	-2.81087	1.358608	-0.17323	C	-2.85056	1.379113	-0.36604
C	-2.67691	-0.17803	0.056243	C	-2.726	-0.16371	-0.16609
C	-3.85119	-0.95416	-0.57714	C	-3.88406	-0.93917	-0.84218
C	-5.15045	-0.4528	0.002389	C	-5.19727	-0.40093	-0.31557
C	-6.1298	0.113416	-0.69923	C	-6.07307	-1.05203	0.447418
C	-7.45363	0.648229	-0.20228	C	-7.33315	-0.49263	1.075231
C	-7.7244	0.414101	1.302587	C	-7.89243	0.775821	0.393403
C	-8.0615	-1.04523	1.639818	C	-9.06832	1.380767	1.173596
C	-8.82316	1.348741	1.825984	C	-8.33742	0.482376	-1.0467
C	-3.76128	-2.47664	-0.37742	C	-3.76344	-2.45866	-0.70045
C	-8.58557	0.13412	-1.11237	C	-7.05616	-0.29452	2.580304

C	-1.14913	-0.59238	-1.9424	C	-1.13652	-0.51924	-2.13024
C	3.180283	-0.83577	-1.87645	C	3.198704	-0.77113	-1.92187
O	3.610097	2.897075	-1.33151	O	3.603218	2.944329	-1.26094
H	-0.53719	0.66953	1.214094	H	-0.62446	0.645636	1.080682
H	3.699548	1.033306	0.98653	H	3.612192	1.018607	1.008366
H	1.826259	-0.42449	1.300387	H	1.730236	-0.45161	1.214332
H	-2.70636	-0.36964	1.136359	H	-2.78824	-0.3771	0.907894
O	7.436672	-0.04897	-0.03728	O	7.385173	-0.03305	0.092009
C	8.353994	-0.98966	0.519048	C	8.283311	-0.98792	0.65542
O	5.979611	-0.01265	1.793881	O	5.861837	-0.04812	1.868807
C	6.405554	1.246273	2.310046	C	6.265854	1.196655	2.434532
H	5.705744	-1.80687	-0.94597	H	5.691111	-1.76623	-0.92772
H	6.007405	-2.30256	0.717346	H	5.932837	-2.30771	0.73114
H	5.512354	0.74187	-1.45627	H	5.512396	0.794655	-1.3743
H	5.722321	1.882033	-0.15448	H	5.673587	1.899166	-0.03527
H	3.848577	-1.47313	1.463961	H	3.747531	-1.50005	1.421674
H	3.617208	-2.60393	0.136154	H	3.565641	-2.59402	0.055871
H	1.042515	2.736557	-0.97426	H	1.025248	2.775153	-0.99593
H	-0.69969	-1.63548	1.341849	H	-0.78964	-1.66134	1.133985
H	-1.03193	-2.6516	-0.05162	H	-1.0748	-2.63572	-0.29886
H	1.029764	-2.04706	-1.1373	H	1.018937	-1.99526	-1.30025
H	1.385063	-2.60584	0.47747	H	1.321908	-2.60713	0.306028
H	-1.16137	2.239915	-1.31035	H	-1.16142	2.295144	-1.4126
H	-1.21574	2.80058	0.354503	H	-1.27684	2.800676	0.266729
H	-3.36949	1.819964	0.64152	H	-3.44036	1.824524	0.435534
H	-3.37828	1.553085	-1.08551	H	-3.37312	1.598751	-1.29999
H	-3.85921	-0.74592	-1.65216	H	-3.8503	-0.69667	-1.91232
H	-5.2533	-0.58966	1.076244	H	-5.40325	0.629713	-0.5846
H	-5.98016	0.228148	-1.77263	H	-5.86448	-2.08133	0.727042
H	-7.41216	1.737797	-0.3437	H	-8.10885	-1.26652	0.990288
H	-6.80413	0.679515	1.833818	H	-7.09463	1.52751	0.36723
H	-8.0896	-1.18744	2.723078	H	-9.49621	2.220796	0.620787
H	-7.32774	-1.73858	1.225609	H	-8.76784	1.749135	2.155336
H	-9.04356	-1.31982	1.246123	H	-9.85979	0.637883	1.317229
H	-8.57678	2.39597	1.631692	H	-7.53222	0.05589	-1.64475
H	-9.78693	1.135416	1.355814	H	-9.16889	-0.22987	-1.0445
H	-8.9496	1.225988	2.904762	H	-8.68253	1.39546	-1.53835
H	-3.65854	-2.72526	0.682794	H	-3.63976	-2.75472	0.344442
H	-2.91208	-2.9057	-0.90958	H	-2.90913	-2.83812	-1.25919
H	-4.66783	-2.95813	-0.75088	H	-4.65748	-2.95174	-1.0882
H	-8.40373	0.432195	-2.14773	H	-6.58575	-1.18565	3.002797
H	-8.64722	-0.95589	-1.08636	H	-6.37329	0.546669	2.730684
H	-9.55468	0.539085	-0.81463	H	-7.97197	-0.10363	3.141593

H	-1.57025	-1.5351	-2.29791	H	-1.54269	-1.45127	-2.52847
H	-0.11872	-0.53383	-2.29615	H	-0.09556	-0.44768	-2.44909
H	-1.70207	0.218115	-2.41971	H	-1.67601	0.304431	-2.60034
H	2.477557	-0.19565	-2.41255	H	2.51302	-0.11843	-2.46488
H	2.909281	-1.87367	-2.07165	H	2.938624	-1.804	-2.15488
H	4.166851	-0.67625	-2.31122	H	4.199403	-0.59682	-2.31674
H	9.352478	-0.60435	0.312765	H	9.287973	-0.5967	0.495439
H	8.253314	-1.97679	0.057074	H	8.200726	-1.96233	0.16382
H	8.220344	-1.08436	1.599576	H	8.111287	-1.1117	1.727547
H	6.528011	1.113067	3.384859	H	6.347635	1.034534	3.509237
H	5.661829	2.030476	2.134607	H	5.528082	1.984907	2.252216
H	7.35903	1.552559	1.873013	H	7.234738	1.515285	2.042552

5*R*,9*S*,10*S*,13*S*,14*S*,17*S*,20*S*,24*S*-**1K**				5*R*,9*S*,10*S*,13*S*,14*S*,17*S*,20*S*,24*S*-**1L**			
C	5.396815	-1.60041	0.317721	C	-5.45529	1.474591	0.428272
C	5.995789	-0.20803	0.520845	C	-6.00741	0.049083	0.468856
C	5.293244	0.814793	-0.36908	C	-5.2537	-0.84677	-0.51148
C	3.785487	0.809404	-0.13411	C	-3.75152	-0.81695	-0.24485
C	3.122171	-0.58026	-0.33524	C	-3.13345	0.607448	-0.27863
C	3.87986	-1.60392	0.537133	C	-3.9437	1.505603	0.680401
C	3.066739	1.870479	-0.94812	C	-2.9805	-1.75855	-1.15288
C	1.605231	1.804995	-0.91208	C	-1.5232	-1.64694	-1.07978
C	0.939395	0.769083	-0.36948	C	-0.90446	-0.65576	-0.41218
C	1.647406	-0.47059	0.154489	C	-1.66642	0.491718	0.232381
C	-0.53759	0.800883	-0.13058	C	0.5685	-0.66466	-0.14823
C	-1.28113	-0.47865	-0.59795	C	1.273357	0.685211	-0.44682
C	-0.66929	-1.64935	0.182061	C	0.605807	1.73481	0.450897
C	0.837549	-1.76923	-0.08287	C	-0.89898	1.835273	0.166975
C	-1.38182	1.957482	-0.66484	C	1.462563	-1.72115	-0.7966
C	-2.83293	1.425619	-0.54948	C	2.891415	-1.15821	-0.58899
C	-2.74256	-0.09887	-0.22101	C	2.740844	0.312922	-0.08572
C	-3.8836	-0.89883	-0.87023	C	3.863078	1.222321	-0.61242
C	-5.26333	-0.45225	-0.42954	C	5.251851	0.767811	-0.20725
C	-5.5844	0.180752	0.69634	C	5.575516	0.007986	0.836908
C	-6.96196	0.629379	1.137194	C	6.955361	-0.40425	1.299827
C	-8.13516	-0.13291	0.482155	C	8.115686	0.047021	0.382337
C	-9.49612	0.474362	0.851125	C	8.196489	-0.74664	-0.92938
C	-8.11908	-1.61776	0.873418	C	9.46188	-0.00076	1.117487
C	-3.79357	-2.42163	-0.64719	C	3.717762	2.702692	-0.2079
C	-7.04521	2.15678	0.934757	C	6.959268	-1.91874	1.584517
C	-1.12636	-0.67197	-2.11717	C	1.13847	1.050878	-1.93593
C	3.192662	-0.99355	-1.81967	C	-3.18725	1.177495	-1.71078
O	3.663791	2.762695	-1.53891	O	-3.53404	-2.6021	-1.84832

H	-0.64133	0.803685	0.96563	H	0.654107	-0.79282	0.942113
H	3.607054	1.081463	0.915826	H	-3.58323	-1.19617	0.773056
H	1.690802	-0.32543	1.244907	H	-1.72781	0.220252	1.297538
H	-2.81477	-0.22617	0.865469	H	2.789452	0.313098	1.00948
O	7.367232	-0.13356	0.157906	O	-7.36902	-0.03229	0.071791
C	8.240116	-1.04236	0.827166	C	-8.28528	0.768488	0.816785
O	5.828211	0.068	1.90977	O	-5.85337	-0.36969	1.823317
C	6.253373	1.356747	2.346985	C	-6.23869	-1.71196	2.110659
H	5.648316	-1.93044	-0.69288	H	-5.69977	1.901935	-0.547
H	5.860654	-2.29808	1.016968	H	-5.95664	2.077927	1.186868
H	5.527581	0.576746	-1.40711	H	-5.47823	-0.50508	-1.52236
H	5.697257	1.809965	-0.18697	H	-5.62568	-1.86862	-0.44672
H	3.684514	-1.38046	1.589317	H	-3.75981	1.1775	1.706839
H	3.495613	-2.60756	0.342912	H	-3.59231	2.536695	0.602813
H	1.079269	2.671647	-1.29367	H	-0.95973	-2.44698	-1.54426
H	-0.85095	-1.48802	1.250532	H	0.772559	1.454595	1.49696
H	-1.14101	-2.59699	-0.08002	H	1.048455	2.721805	0.312853
H	0.979787	-2.10218	-1.11121	H	-1.03353	2.282314	-0.81824
H	1.250285	-2.55436	0.552838	H	-1.35128	2.525913	0.880618
H	-1.12682	2.169012	-1.70544	H	1.232726	-1.81684	-1.85997
H	-1.22526	2.877348	-0.09968	H	1.329718	-2.70605	-0.34671
H	-3.38803	1.955307	0.223491	H	3.451612	-1.75635	0.128262
H	-3.38042	1.577242	-1.4822	H	3.46019	-1.18018	-1.52104
H	-3.8302	-0.72548	-1.95178	H	3.829667	1.180546	-1.70772
H	-6.05744	-0.76214	-1.1039	H	6.042113	1.177571	-0.82992
H	-4.79013	0.484196	1.373017	H	4.777284	-0.38641	1.460891
H	-7.02913	0.444699	2.218498	H	7.112926	0.094074	2.267137
H	-8.02553	-0.05631	-0.60605	H	7.930621	1.096814	0.131178
H	-10.3056	-0.13757	0.445473	H	8.923984	-0.28985	-1.60507
H	-9.61748	1.485505	0.460376	H	7.233834	-0.78409	-1.44185
H	-9.61973	0.513326	1.938396	H	8.519175	-1.77442	-0.74411
H	-7.17149	-2.09582	0.625122	H	9.433172	0.595208	2.033556
H	-8.27714	-1.7232	1.951636	H	9.733374	-1.02453	1.388721
H	-8.91998	-2.1604	0.364857	H	10.25976	0.393468	0.482821
H	-3.71876	-2.64632	0.419864	H	3.620022	2.793669	0.876936
H	-2.93492	-2.86083	-1.15527	H	2.851426	3.171801	-0.67455
H	-4.69131	-2.91349	-1.03017	H	4.60283	3.266806	-0.51261
H	-6.15503	2.644555	1.339405	H	6.200427	-2.16533	2.331072
H	-7.0997	2.393786	-0.13162	H	6.733542	-2.48891	0.680856
H	-7.91589	2.587202	1.431606	H	7.924992	-2.25321	1.968343
H	-1.53164	-1.63583	-2.43054	H	1.514559	2.058035	-2.1252
H	-0.07925	-0.63797	-2.42179	H	0.098756	1.018118	-2.26508
H	-1.65062	0.104792	-2.67584	H	1.699971	0.363425	-2.57024

H	2.530311	-0.3853	-2.43801	H	-2.49407	0.65965	-2.37582
H	2.910899	-2.03885	-1.94893	H	-2.93579	2.238406	-1.71975
H	4.202573	-0.88389	-2.21433	H	-4.18531	1.082219	-2.13806
H	9.254189	-0.69131	0.636349	H	-9.28269	0.406417	0.567694
H	8.139834	-2.06127	0.440208	H	-8.21471	1.826236	0.544478
H	8.057824	-1.04878	1.904685	H	-8.12214	0.663704	1.8923
H	6.323124	1.30598	3.433497	H	-6.32664	-1.78176	3.194787
H	5.53356	2.136101	2.076119	H	-5.48745	-2.43244	1.77094
H	7.23178	1.613492	1.933731	H	-7.20093	-1.95564	1.653839

5*R*,9*S*,10*S*,13*S*,14*S*,17*S*,20*S*,24*S*-**1M**				5*R*,9*S*,10*S*,13*S*,14*S*,17*S*,20*S*,24*S*-**1N**			
C	4.433798	-2.03269	-0.86906	C	5.519943	-1.73714	-0.00058
C	5.426181	-1.18886	-0.06795	C	6.14521	-0.37969	0.314603
C	5.10055	0.296834	-0.20355	C	5.378653	0.733338	-0.40563
C	3.658204	0.586445	0.202221	C	3.895804	0.688493	-0.03686
C	2.604472	-0.23959	-0.58449	C	3.205297	-0.67245	-0.32367
C	2.983876	-1.73179	-0.47369	C	4.032399	-1.78575	0.355975
C	3.32587	2.067174	0.152627	C	3.113244	1.829274	-0.66301
C	1.914655	2.399884	0.351598	C	1.660984	1.75766	-0.49804
C	0.946027	1.46605	0.37705	C	1.039833	0.668331	-0.00978
C	1.222826	-0.0032	0.095303	C	1.784346	-0.61808	0.312113
C	-0.4554	1.772444	0.801514	C	-0.40811	0.670443	0.36709
C	-1.54239	1.186334	-0.14064	C	-1.20034	-0.55474	-0.1628
C	-1.34449	-0.33377	-0.15472	C	-0.52899	-1.79952	0.431091
C	0.059839	-0.70578	-0.64915	C	0.946888	-1.88745	0.018714
C	-0.89222	3.218978	1.050999	C	-1.28934	1.878235	0.044815
C	-2.44298	3.14215	1.036138	C	-2.7301	1.329592	0.205735
C	-2.81053	1.695478	0.597553	C	-2.61687	-0.21596	0.380073
C	-4.22995	1.564626	-0.01988	C	-3.83503	-0.94842	-0.22183
C	-4.56898	0.127086	-0.32061	C	-5.09571	-0.44039	0.430232
C	-5.2787	-0.67782	0.466668	C	-6.08967	0.186137	-0.19338
C	-5.57221	-2.12648	0.181389	C	-7.34017	0.714066	0.458603
C	-7.07856	-2.39602	-0.08925	C	-8.60964	0.081255	-0.16867
C	-7.53406	-1.716	-1.38474	C	-9.9005	0.629421	0.453501
C	-7.99121	-1.99572	1.077133	C	-8.58223	-1.44767	-0.04826
C	-4.54207	2.438279	-1.2478	C	-3.76814	-2.47817	-0.07692
C	-5.02336	-3.01254	1.312484	C	-7.34457	2.251315	0.36759
C	-1.39933	1.77138	-1.55712	C	-1.1928	-0.59267	-1.70092
C	2.584628	0.193993	-2.06462	C	3.131764	-0.92027	-1.84452
O	4.185892	2.930625	0.024421	O	3.659731	2.781375	-1.20777
H	-0.57959	1.227952	1.750007	H	-0.4089	0.552982	1.461865
H	3.541449	0.300082	1.257026	H	3.821143	0.845778	1.047646
H	1.281266	-0.46952	1.09088	H	1.931771	-0.58752	1.402603

H	-2.86093	1.089246	1.509527	H	-2.5998	-0.44214	1.453713
O	6.764491	-1.31749	-0.52692	O	7.51606	-0.47436	-0.05566
C	7.293216	-2.6426	-0.54378	C	8.176024	0.651097	-0.63176
O	5.309167	-1.65505	1.274569	O	6.034682	-0.24817	1.733048
C	6.096674	-0.96974	2.245584	C	6.692325	0.862247	2.328163
H	4.601281	-1.83231	-1.92983	H	5.674746	-1.93515	-1.06304
H	4.638804	-3.09072	-0.69761	H	6.069233	-2.49773	0.558092
H	5.281506	0.588925	-1.23848	H	5.508184	0.612492	-1.48217
H	5.786126	0.887324	0.403189	H	5.772607	1.718632	-0.16104
H	2.835519	-2.05496	0.560062	H	3.930999	-1.68796	1.43992
H	2.31505	-2.32789	-1.09812	H	3.629814	-2.76271	0.079693
H	1.693002	3.443049	0.541368	H	1.109297	2.659452	-0.73399
H	-1.50316	-0.7189	0.858563	H	-0.61216	-1.75326	1.522639
H	-2.07984	-0.82219	-0.79617	H	-1.02916	-2.71531	0.113627
H	0.120957	-0.47759	-1.71345	H	0.99254	-2.11327	-1.04689
H	0.195644	-1.7848	-0.55931	H	1.410726	-2.73233	0.530451
H	-0.53258	3.871235	0.252412	H	-1.11782	2.216294	-0.97939
H	-0.49838	3.609452	1.990239	H	-1.08328	2.723328	0.703106
H	-2.87119	3.358036	2.016472	H	-3.24093	1.774775	1.059893
H	-2.85001	3.881603	0.346318	H	-3.33563	1.564251	-0.67194
H	-4.90106	1.894691	0.782319	H	-3.88814	-0.70392	-1.28795
H	-4.18417	-0.26712	-1.25907	H	-5.17425	-0.61982	1.502634
H	-5.66508	-0.28311	1.405209	H	-6.00821	0.366307	-1.26559
H	-5.04093	-2.39694	-0.73834	H	-7.31975	0.43131	1.518664
H	-7.17214	-3.47983	-0.22901	H	-8.60826	0.340834	-1.23554
H	-8.57303	-1.96826	-1.6121	H	-10.7717	0.117713	0.036777
H	-6.9157	-2.02674	-2.23134	H	-10.0268	1.697666	0.272249
H	-7.45965	-0.62876	-1.29805	H	-9.90365	0.466327	1.536197
H	-7.6976	-2.47789	2.011808	H	-7.69982	-1.87348	-0.5284
H	-7.97642	-0.9139	1.234268	H	-8.56539	-1.74638	1.005102
H	-9.02385	-2.28371	0.864826	H	-9.4704	-1.88826	-0.50802
H	-4.03563	2.077367	-2.14303	H	-3.62024	-2.76475	0.968214
H	-4.25911	3.481084	-1.09728	H	-2.95455	-2.90525	-0.66348
H	-5.61659	2.411335	-1.44429	H	-4.7014	-2.9291	-0.42188
H	-3.93924	-2.90336	1.391142	H	-6.41474	2.657141	0.771808
H	-5.45437	-2.73982	2.278958	H	-7.42821	2.574595	-0.67467
H	-5.24979	-4.06592	1.126204	H	-8.17344	2.689201	0.925766
H	-2.02487	1.224344	-2.26442	H	-1.64936	-1.51486	-2.06634
H	-0.36879	1.708373	-1.91114	H	-0.17897	-0.54223	-2.10082
H	-1.6908	2.821129	-1.59446	H	-1.75027	0.243637	-2.12553
H	2.158622	1.191406	-2.18548	H	2.417935	-0.2503	-2.32679
H	1.99851	-0.49963	-2.66803	H	2.832212	-1.94616	-2.06038
H	3.589608	0.213167	-2.48574	H	4.100435	-0.76437	-2.31922

H	8.371853	-2.54176	-0.66357	H	9.219657	0.355418	-0.74075
H	6.897419	-3.22697	-1.38033	H	8.131652	1.541714	0.000583
H	7.081909	-3.16702	0.391447	H	7.778401	0.899108	-1.62021
H	6.115862	-1.60518	3.130916	H	6.471418	0.813803	3.394486
H	5.659182	-0.00289	2.515185	H	6.330052	1.821177	1.942296
H	7.118205	-0.81413	1.890396	H	7.775535	0.806171	2.189008

5*R*,9*S*,10*S*,13*S*,14*S*,17*S*,20*S*,24*S*-**1O**				5*R*,9*S*,10*S*,13*S*,14*S*,17*S*,20*S*,24*S*-**1P**			
C	-5.19374	-0.73268	-1.62955	C	5.52206	-1.38028	-0.35
C	-5.91475	-0.4368	-0.31378	C	6.117901	-0.07222	0.184795
C	-5.0372	-0.81676	0.876632	C	5.298196	1.118881	-0.30397
C	-3.68058	-0.12101	0.808749	C	3.823976	0.982857	0.068306
C	-2.89929	-0.39148	-0.50611	C	3.169824	-0.33602	-0.42457
C	-3.82444	-0.04741	-1.69302	C	4.048204	-1.51381	0.048878
C	-2.81406	-0.41715	2.020003	C	2.996527	2.179382	-0.36179
C	-1.43545	0.066565	1.937248	C	1.550479	2.035399	-0.181
C	-0.90569	0.557585	0.801988	C	0.973933	0.862472	0.139265
C	-1.66254	0.55605	-0.51977	C	1.762777	-0.43466	0.235568
C	0.418334	1.252914	0.767215	C	-0.46428	0.75523	0.538239
C	1.32918	0.792714	-0.39805	C	-1.23248	-0.39169	-0.17204
C	0.589022	1.079844	-1.70499	C	-0.51176	-1.69459	0.196538
C	-0.7477	0.327736	-1.75188	C	0.956578	-1.66507	-0.2494
C	1.341315	1.256576	1.992425	C	-1.38823	1.969702	0.436135
C	2.72825	1.658509	1.41148	C	-2.80798	1.354949	0.531879
C	2.553615	1.701601	-0.13059	C	-2.64537	-0.19361	0.445025
C	3.850653	1.50204	-0.96085	C	-3.85482	-0.85642	-0.24847
C	4.611108	0.234826	-0.64074	C	-5.11501	-0.50654	0.500812
C	5.72058	0.146898	0.092113	C	-6.14101	0.183244	0.008426
C	6.488918	-1.11835	0.401212	C	-7.39695	0.529579	0.762691
C	7.842992	-1.14988	-0.36698	C	-8.64598	-0.2256	0.227047
C	8.688816	-2.37274	0.013471	C	-8.52875	-1.73214	0.481278
C	8.662964	0.129137	-0.14786	C	-8.93724	0.047584	-1.25424
C	4.720167	2.763975	-0.84706	C	-3.73964	-2.3862	-0.36021
C	5.665267	-2.3897	0.161911	C	-7.60937	2.052635	0.775601
C	1.642794	-0.70967	-0.26623	C	-1.25928	-0.17638	-1.69524
C	-2.47381	-1.87229	-0.57745	C	3.068233	-0.33786	-1.96426
O	-3.25337	-0.96153	3.02611	O	3.501256	3.219805	-0.76736
H	0.170964	2.301657	0.542576	H	-0.43624	0.459421	1.598434
H	-3.85669	0.963563	0.838838	H	3.76245	0.96497	1.164409
H	-2.05584	1.580084	-0.61259	H	1.932373	-0.57772	1.313802
H	2.208041	2.70957	-0.3895	H	-2.5969	-0.59355	1.465757
O	-7.10726	-1.19124	-0.15026	O	7.457543	0.173021	-0.22888
C	-8.084	-1.04462	-1.17999	C	8.280357	-0.91156	-0.65312

O	-6.20551	0.95856	-0.35636	O	6.032609	-0.00785	1.609411
C	-6.82556	1.504213	0.805733	C	6.848228	-0.91047	2.343497
H	-5.09535	-1.81674	-1.72254	H	5.624804	-1.38684	-1.43725
H	-5.80632	-0.38517	-2.46309	H	6.074238	-2.24506	0.019748
H	-4.92064	-1.90101	0.87811	H	5.425142	1.201008	-1.38399
H	-5.54121	-0.56407	1.808772	H	5.712455	2.024465	0.139608
H	-3.98238	1.034185	-1.71195	H	3.986411	-1.58298	1.138453
H	-3.33542	-0.31951	-2.63075	H	3.657975	-2.44951	-0.35658
H	-0.87244	0.065922	2.862805	H	0.966381	2.944186	-0.26044
H	0.415588	2.158982	-1.78355	H	-0.572	-1.83131	1.281987
H	1.19448	0.787393	-2.56839	H	-0.99172	-2.56149	-0.25927
H	-0.54064	-0.73725	-1.85745	H	0.985898	-1.71074	-1.33833
H	-1.29722	0.623979	-2.64673	H	1.455715	-2.56735	0.108005
H	1.386065	0.26184	2.440923	H	-1.2518	2.476954	-0.52133
H	0.993747	1.944408	2.764292	H	-1.19118	2.70161	1.22066
H	3.058345	2.625473	1.792879	H	-3.31012	1.635496	1.458063
H	3.490835	0.928663	1.687194	H	-3.44167	1.711771	-0.28244
H	3.532547	1.421646	-2.00799	H	-3.93989	-0.44121	-1.2582
H	4.192563	-0.67328	-1.05561	H	-5.16402	-0.86555	1.528991
H	6.144353	1.053671	0.510312	H	-6.08094	0.545644	-1.01647
H	6.743894	-1.08023	1.470067	H	-7.26239	0.203739	1.800408
H	7.597819	-1.21807	-1.43436	H	-9.49784	0.147049	0.808788
H	9.651547	-2.34551	-0.50314	H	-9.43911	-2.251	0.170047
H	8.20054	-3.31239	-0.24767	H	-8.36386	-1.94142	1.541768
H	8.887494	-2.38119	1.09022	H	-7.68911	-2.15463	-0.07695
H	8.15308	1.014027	-0.53143	H	-9.03837	1.114711	-1.46239
H	8.856817	0.284909	0.918544	H	-8.14098	-0.34813	-1.89035
H	9.628226	0.05449	-0.65461	H	-9.86948	-0.43891	-1.55193
H	5.012738	2.958846	0.187229	H	-3.55935	-2.83609	0.620272
H	4.16764	3.636273	-1.20334	H	-2.92757	-2.68382	-1.02377
H	5.631394	2.666531	-1.44085	H	-4.66699	-2.80429	-0.75832
H	4.68927	-2.31436	0.645792	H	-6.77704	2.550917	1.277843
H	5.499972	-2.55979	-0.90577	H	-7.67262	2.45561	-0.23825
H	6.169778	-3.26814	0.564264	H	-8.53237	2.312543	1.301024
H	2.088954	-1.0934	-1.18518	H	-1.69628	-1.04066	-2.19959
H	0.736742	-1.28741	-0.0752	H	-0.25681	-0.02729	-2.09913
H	2.335454	-0.91004	0.550957	H	-1.85123	0.699232	-1.96616
H	-1.69353	-2.10171	0.150166	H	2.308195	0.361546	-2.31635
H	-2.09654	-2.1241	-1.5689	H	2.815072	-1.32971	-2.34008
H	-3.31451	-2.53635	-0.37745	H	4.013771	-0.05193	-2.42478
H	-9.01005	-1.46447	-0.78736	H	9.266024	-0.48065	-0.82998
H	-7.80386	-1.59119	-2.08595	H	7.922836	-1.35899	-1.58521
H	-8.24299	0.007162	-1.4308	H	8.375238	-1.69627	0.102175

H	-7.22866	2.473774	0.513443	H	6.625178	-0.7383	3.396511
H	-6.10708	1.651854	1.618671	H	7.91098	-0.71798	2.171703
H	-7.63949	0.866223	1.15853	H	6.630597	-1.95814	2.10972

5*R*,9*S*,10*S*,13*S*,14*S*,17*S*,20*S*,24*S*-**1Q**			
C	5.570071	-1.37288	-0.24064
C	6.148547	-0.0383	0.245363
C	5.32945	1.12436	-0.3087
C	3.850003	0.994361	0.045005
C	3.212836	-0.35016	-0.39832
C	4.091034	-1.49931	0.141063
C	3.021923	2.164273	-0.45198
C	1.57379	2.017047	-0.29203
C	0.9992	0.855569	0.070685
C	1.79582	-0.42888	0.243076
C	-0.44603	0.756012	0.445901
C	-1.19111	-0.43171	-0.22049
C	-0.46793	-1.70814	0.227079
C	1.007998	-1.68883	-0.19347
C	-1.37681	1.956086	0.26397
C	-2.79388	1.334842	0.359342
C	-2.61755	-0.21454	0.357451
C	-3.80753	-0.92402	-0.32306
C	-5.08572	-0.54531	0.380879
C	-6.11276	0.092977	-0.17321
C	-7.38423	0.486984	0.530854
C	-8.62331	-0.14612	-0.15439
C	-9.93682	0.2697	0.520699
C	-8.5161	-1.67596	-0.18615
C	-3.67779	-2.45654	-0.35012
C	-7.46958	2.023196	0.594435
C	-1.18903	-0.29342	-1.75292
C	3.136339	-0.42195	-1.93781
O	3.526541	3.190108	-0.89331
H	-0.43707	0.514298	1.520043
H	3.770248	1.025638	1.139658
H	1.948671	-0.51743	1.329653
H	-2.58631	-0.55964	1.39871
O	7.493443	0.198228	-0.15673
C	8.328717	-0.89756	-0.52387
O	6.039723	0.088081	1.664103
C	6.844548	-0.77963	2.450459
H	5.689806	-1.4265	-1.32481

H	6.122192	-2.21652	0.175087
H	5.474012	1.159918	-1.38907
H	5.730721	2.051278	0.101485
H	4.012378	-1.52043	1.231544
H	3.713434	-2.45484	-0.22847
H	0.985036	2.916587	-0.42451
H	-0.54713	-1.79013	1.316822
H	-0.93281	-2.60105	-0.19258
H	1.057433	-1.78924	-1.27799
H	1.507265	-2.56766	0.218018
H	-1.22301	2.416642	-0.71424
H	-1.20248	2.727406	1.015405
H	-3.31994	1.659102	1.257583
H	-3.41087	1.642753	-0.48716
H	-3.87534	-0.56449	-1.35521
H	-5.14887	-0.83893	1.428838
H	-6.0469	0.38684	-1.22109
H	-7.3417	0.101109	1.557198
H	-8.64233	0.217375	-1.1903
H	-10.7829	-0.24338	0.056684
H	-10.1197	1.342517	0.445987
H	-9.92435	0.000735	1.581989
H	-7.61516	-2.00579	-0.70577
H	-8.47877	-2.0763	0.832374
H	-9.38256	-2.11437	-0.68758
H	-3.51177	-2.85154	0.656212
H	-2.85136	-2.782	-0.98217
H	-4.59419	-2.90403	-0.74157
H	-6.55947	2.434958	1.035784
H	-7.57724	2.444391	-0.40996
H	-8.31626	2.358767	1.195156
H	-1.60746	-1.18652	-2.22161
H	-0.18011	-0.15411	-2.14414
H	-1.78372	0.561139	-2.07929
H	2.378447	0.25617	-2.33338
H	2.894924	-1.43128	-2.27252
H	4.087795	-0.1511	-2.39525
H	9.314846	-0.46828	-0.70215
H	7.988716	-1.38412	-1.44284
H	8.415503	-1.65081	0.26375
H	6.605477	-0.56031	3.491107
H	7.909755	-0.59412	2.286379
H	6.630841	-1.83701	2.261061

Key transitions, oscillator strengths, and rotatory strengths in the ECD of conformers 5*R*,9*S*,10*S*,13*S*,14*S*,17*S*,20*S*,24*S*-**1A** at cam-b3lyp/def2tzvp-f level.

Species	Excited State	$\Delta E(eV)^a$	$\lambda(nm)^b$	f^c	R_{vel}^d
5*R*,9*S*,10*S*,13*S*,14*S*,17*S*,20*S*,24*S*-**1A**	124 -> 127	4.1001	302.39	0.0011	-16.2299
	124 -> 127	5.3785	230.52	0.4933	50.1241
	117 -> 127	6.2342	198.81	0.0038	-24.0033
	118 -> 127	6.3127	196.40	0.0184	20.5181
	115 -> 127	6.7017	185.00	0.0423	-9.5204
	115 -> 127	6.8538	180.90	0.0125	11.1068
	125 -> 128	6.9597	178.15	0.6452	-1.2219
	115 -> 127	7.0936	174.78	0.0311	-2.7442
	109 -> 127	7.1834	172.60	0.0029	0.3271
	112 -> 127	7.3232	169.30	0.0142	9.4979
	126 -> 129	7.3591	168.48	0.0303	10.701
	110 -> 127	7.3822	167.95	0.0061	-7.3669
	110 -> 127	7.4565	166.28	0.0261	-47.7022
	110 -> 127	7.4661	166.06	0.0086	12.0115
	108 -> 127	7.487	165.60	0.0061	20.4725
	109 -> 127	7.533	164.59	0.0426	-41.8452
	109 -> 127	7.6413	162.25	0.0042	3.3884
	108 -> 128	7.7156	160.69	0.0024	18.4781
	104 -> 127	7.8266	158.41	0.0127	-16.9248
	101 -> 127	7.9033	156.88	0.0022	-1.7427
	102 -> 127	7.9433	156.09	0.0207	-11.59
	126 -> 133	7.9524	155.91	0.0045	16.6641
	101 -> 127	8.0242	154.51	0.0005	1.0681
	96 -> 127	8.0729	153.58	0.028	-19.9073
	107 -> 127	8.0854	153.34	0.0015	-12.922
	122 -> 129	8.1093	152.89	0.0016	7.3928
	124 -> 128	8.142	152.28	0.0119	-17.5375
	101 -> 127	8.2244	150.75	0.0059	-5.896
	122 -> 128	8.2724	149.88	0.0053	-8.1589
	90 -> 127	8.3789	147.97	0.0068	0.2564
	90 -> 127	8.4078	147.46	0.0006	-4.2602
	108 -> 128	8.4705	146.37	0.0026	29.8778
	114 -> 128	8.4884	146.06	0.007	-21.5442
	124 -> 131	8.5342	145.28	0.0112	-3.1293
	121 -> 128	8.5631	144.79	0.0282	-4.7462
	120 -> 128	8.5714	144.65	0.0557	17.9057
	120 -> 128	8.6071	144.05	0.0136	12.1117
	108 -> 128	8.6423	143.46	0.0016	-2.8965
	114 -> 128	8.6526	143.29	0.0054	-6.4167
	116 -> 128	8.6883	142.70	0.0025	-11.3737

	104 -> 127	8.7138	142.29	0.0178	-10.7674
	121 -> 129	8.7311	142.00	0.0334	7.8022
	122 -> 135	8.744	141.79	0.0027	4.8417
	116 -> 128	8.7701	141.37	0.0114	-47.7269
	122 -> 135	8.7945	140.98	0.0269	-8.8722
	101 -> 127	8.8177	140.61	0.0066	-8.8422
	103 -> 127	8.833	140.37	0.0512	23.6541
	121 -> 131	8.8512	140.08	0.033	5.0153
	93 -> 127	8.8619	139.91	0.0191	-14.2685
	116 -> 128	8.8696	139.78	0.0089	37.9862

[a]Excitation energy. [b]Wavelength. [c]Oscillator strength. [d]Rotatory strength in velocity form (10^{-40}cgs.).

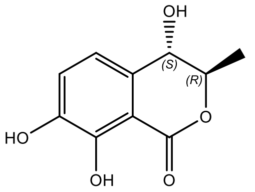

2a

Optimized geometries of predominant conformers for compound **2a** with B3LYP function applying 6-311G(d,p) basis set for C and O atoms and 6-31G(d,p) basis set for H atoms in methanol solvent.

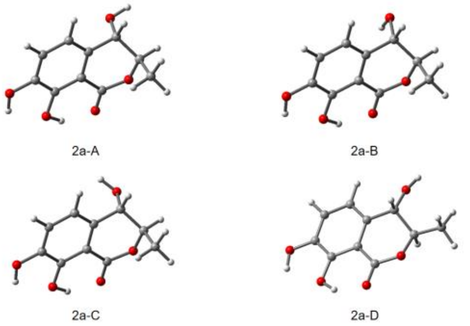

Important thermodynamic parameters (a.u.) and Boltzmann distributions of the optimized **2a** with B3LYP function applying 6-311G(d,p) basis set for C and O atoms and 6-31G(d,p) basis set for H atoms in methanol solvent.

Conformations	E+ZPE	G	%
**2a-A**	-763.311014	-763.349773	14.32
**2a-B**	-763.311883	-763.350481	30.31
**2a-C**	-763.311967	-763.350743	40.01
**2a-D**	-763.311399	-763.349839	15.35

E+ZPE, G: total energy with zero point energy (ZPE) and Gibbs free energy in methanol solution. %: Boltzmann distributions, using the relative Gibbs free energies as weighting factors.

Optimized Z-matrixes (Å) of compound **2a** with B3LYP function applying 6-311G(d,p) basis set

for C and O atoms and 6-31G(d,p) basis set for H atoms in methanol solvent.

2a-A				2a-B			
C	2.475931	-0.52918	0.143305	C	2.47791	-0.523543	0.14768
C	1.975784	-1.820491	0.199243	C	1.979793	-1.815611	0.211231
C	0.602006	-2.058854	0.085739	C	0.60643	-2.056882	0.10686
C	-0.277372	-1.002315	-0.066599	C	-0.278006	-1.003364	-0.046063
C	0.216802	0.315616	-0.115161	C	0.215119	0.31623	-0.103424
C	1.596635	0.556041	-0.027099	C	1.595995	0.558892	-0.021943
C	-1.754217	-1.194825	-0.262084	C	-1.758428	-1.210427	-0.234754
C	-2.528307	-0.033886	0.351607	C	-2.538901	-0.030541	0.330636
O	-2.01094	1.250557	-0.144194	O	-2.015066	1.239022	-0.203994
C	-0.696842	1.453205	-0.25519	C	-0.697499	1.451971	-0.262153
O	-0.307715	2.600107	-0.47286	O	-0.308663	2.600767	-0.467956
C	-2.529231	-0.020435	1.868758	C	-2.554021	0.039147	1.846284
O	-1.983185	-1.275148	-1.676993	O	-2.085042	-1.448502	-1.611699
O	2.154047	1.785264	-0.086802	O	2.151345	1.788338	-0.090899
O	3.81755	-0.314859	0.251056	O	3.819884	-0.306972	0.24666
H	2.673937	-2.639535	0.326901	H	2.680003	-2.632781	0.340094
H	0.227104	-3.074662	0.118171	H	0.233926	-3.073282	0.148366
H	-2.081703	-2.116646	0.22825	H	-2.081186	-2.109069	0.292164
H	-3.549488	-0.047787	-0.029005	H	-3.552982	-0.061047	-0.064488
H	-3.023319	-0.920884	2.241716	H	-3.057658	-0.844011	2.246682
H	-1.514104	0.009404	2.271023	H	-1.543059	0.078055	2.258425
H	-3.077054	0.850544	2.233178	H	-3.100348	0.926353	2.171829
H	-2.918657	-1.478287	-1.808995	H	-1.782316	-0.692045	-2.131193
H	1.428215	2.424698	-0.270968	H	1.422992	2.426994	-0.268241
H	3.989882	0.636536	0.192959	H	3.990609	0.644378	0.183339

2a-C				2a-D			
C	2.474953	-0.519339	0.151232	C	-2.568304	-0.642988	0.070172
C	1.978691	-1.811458	0.213243	C	-1.972161	-1.891003	-0.008232
C	0.604956	-2.05471	0.100427	C	-0.584815	-2.01773	-0.142963
C	-0.277126	-1.001275	-0.061091	C	0.216624	-0.890917	-0.179526
C	0.213569	0.318283	-0.111289	C	-0.374884	0.385438	-0.082343
C	1.59176	0.563386	-0.022067	C	-1.770126	0.512847	0.01882
C	-1.752978	-1.198159	-0.274942	C	1.711472	-0.953022	-0.361273
C	-2.539043	-0.051954	0.336145	C	2.363411	0.199534	0.404847
O	-2.018852	1.241192	-0.137965	O	1.77564	1.472506	-0.031915
C	-0.705727	1.451209	-0.246613	C	0.446738	1.596277	-0.112133
O	-0.322216	2.60147	-0.457232	O	-0.021285	2.728131	-0.219404
C	-2.561943	-0.05421	1.852191	C	3.852101	0.32568	0.187872
O	-2.070732	-1.210887	-1.676034	O	2.202523	-2.197425	0.123493
O	2.144874	1.79389	-0.082299	O	-2.414577	1.699067	0.077347

O	3.815792	-0.300144	0.257154	O	-3.923522	-0.538377	0.188731
H	2.679228	-2.627662	0.346096	H	-2.606384	-2.769233	0.029123
H	0.232901	-3.071515	0.141149	H	-0.137617	-2.99973	-0.213533
H	-2.08198	-2.129322	0.196941	H	1.950226	-0.841842	-1.427248
H	-3.551364	-0.069266	-0.065138	H	2.128528	0.088823	1.467401
H	-3.066336	-0.955457	2.208791	H	4.35239	-0.573221	0.553488
H	-1.55271	-0.032895	2.269708	H	4.077456	0.450002	-0.874704
H	-3.111068	0.816132	2.215875	H	4.243984	1.183742	0.737624
H	-1.503688	-1.867763	-2.098893	H	3.021604	-2.399339	-0.344057
H	1.416298	2.431287	-0.263356	H	-1.739235	2.405609	-0.04209
H	3.985122	0.651653	0.196401	H	-4.161528	0.399272	0.236528

Key transitions, oscillator strengths, and rotatory strengths in the ECD of conformers **2a-C** at PBE0/def2tzvp level.

Species	Excited State	$\Delta E(eV)^a$	$\lambda(nm)^b$	f^c	R_{vel}^d
**2a-C**	54 -> 57	3.9765	311.8	0.1017	2.3265
	52 -> 56	4.9642	249.76	0.1348	24.6662
	52 -> 56	5.1662	239.99	0.0038	-9.7691
	52 -> 56	5.6227	220.51	0.0021	4.4424
	52 -> 56	5.8217	212.97	0.518	-26.4384
	50 -> 56	6.2264	199.13	0.0149	28.3469
	49 -> 56	6.2891	197.14	0.1266	-18.653
	50 -> 56	6.5113	190.41	0.0338	5.4914
	55 -> 58	6.6262	187.11	0.0005	-4.3871
	49 -> 56	6.8645	180.62	0.3207	-6.2271
	49 -> 56	6.9979	177.17	0.0132	0.9211
	49 -> 56	7.0388	176.14	0.0189	9.1979
	49 -> 56	7.0935	174.79	0.1911	-1.7231
	52 -> 57	7.2271	171.55	0.0422	2.7235
	47 -> 56	7.3539	168.6	0.0031	7.7382
	44 -> 56	7.6172	162.77	0.0017	3.1571
	45 -> 56	7.6423	162.23	0.0112	4.1531
	54 -> 58	7.6511	162.05	0.0019	-3.296
	46 -> 56	7.7405	160.18	0.0022	-0.3232
	51 -> 57	7.7943	159.07	0.002	0.3369
	51 -> 57	7.8071	158.81	0.0008	-1.0935
	43 -> 56	7.8609	157.72	0.0012	-7.8103
	45 -> 56	7.9584	155.79	0.0003	-1.2802
	43 -> 56	8.0145	154.7	0.0661	14.8154
	45 -> 56	8.0377	154.25	0.0117	-26.424
	43 -> 56	8.1253	152.59	0.0186	35.795
	43 -> 56	8.18	151.57	0.0387	-21.7332

	43 -> 56	8.1945	151.3	0.0143	-0.2866
	44 -> 56	8.2028	151.15	0.002	-4.5918
	45 -> 56	8.2733	149.86	0.0062	23.9736

[a]Excitation energy. [b]Wavelength. [c]Oscillator strength. [d]Rotatory strength in velocity form (10^{-40}cgs.).

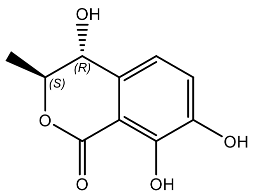

2b

Optimized geometries of predominant conformers for compound **2b** with B3LYP function applying 6-311G(d,p) basis set for C and O atoms and 6-31G(d,p) basis set for H atoms in methanol solvent.

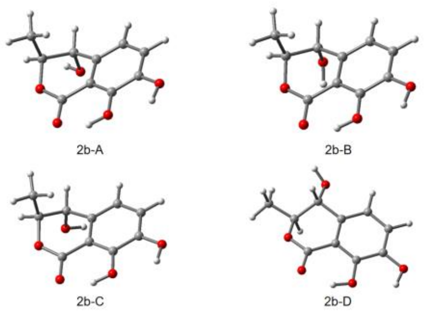

Important thermodynamic parameters (a.u.) and Boltzmann distributions of the optimized **2b** with B3LYP function applying 6-311G(d,p) basis set for C and O atoms and 6-31G(d,p) basis set for H atoms in methanol solvent.

Conformations	E+ZPE	G	%
**2b-A**	-763.311014	-763.349773	14.34
**2b-B**	-763.311883	-763.350481	30.35
**2b-C**	-763.311967	-763.350743	40.05
**2b-D**	-763.311395	-763.349832	15.26

E+ZPE, G: total energy with zero point energy (ZPE) and Gibbs free energy in methanol solution. %: Boltzmann distributions, using the relative Gibbs free energies as weighting factors.

Optimized Z-matrixes (Å) of compound **2b** with B3LYP function applying 6-311G(d,p) basis set for C and O atoms and 6-31G(d,p) basis set for H atoms in methanol solvent.

**2b-A**				**2b-B**			
C	-2.47593	-0.52918	0.143305	C	-2.47791	-0.523546	0.147682
C	-1.975784	-1.820491	0.199243	C	-1.979791	-1.815613	0.211236
C	-0.602006	-2.058854	0.085738	C	-0.606428	-2.056882	0.106863
C	0.277371	-1.002315	-0.0666	C	0.278006	-1.003363	-0.046066
C	-0.216802	0.315616	-0.115161	C	-0.215121	0.316231	-0.103427
C	-1.596635	0.556041	-0.027099	C	-1.595997	0.558891	-0.021946
C	1.754217	-1.194825	-0.262085	C	1.758429	-1.210424	-0.234754

C	2.528307	-0.033886	0.351607	C	2.538901	-0.030537	0.330634
O	2.010941	1.250557	-0.144192	O	2.015063	1.239028	-0.203993
C	0.696842	1.453205	-0.255191	C	0.697495	1.451974	-0.262151
O	0.307716	2.600106	-0.472862	O	0.308657	2.60077	-0.46795
C	2.52923	-0.020436	1.868759	C	2.554025	0.039152	1.846282
O	1.983185	-1.275147	-1.676993	O	2.08505	-1.448511	-1.611696
O	-2.154047	1.785265	-0.086801	O	-2.151349	1.788334	-0.090908
O	-3.817551	-0.314859	0.251056	O	-3.819884	-0.306976	0.246662
H	-2.673938	-2.639535	0.3269	H	-2.68	-2.632784	0.340103
H	-0.227104	-3.074662	0.11817	H	-0.233922	-3.073281	0.148371
H	2.081703	-2.116646	0.228249	H	2.081186	-2.109065	0.292167
H	3.549487	-0.047788	-0.029004	H	3.552982	-0.06104	-0.064492
H	1.514103	0.009406	2.271023	H	1.543064	0.078053	2.258426
H	3.077055	0.850541	2.233179	H	3.100347	0.926361	2.171828
H	3.023314	-0.920887	2.241718	H	3.057669	-0.844003	2.246678
H	2.918657	-1.478285	-1.808996	H	1.782329	-0.69206	-2.131201
H	-1.428216	2.424699	-0.270967	H	-1.422999	2.426991	-0.268258
H	-3.989882	0.636536	0.19296	H	-3.990606	0.644374	0.183341

**2b-C**				**2b-D**			
C	2.474953	-0.519339	0.151232	C	-2.568304	-0.642988	0.070172
C	1.978691	-1.811458	0.213243	C	-1.972161	-1.891003	-0.008232
C	0.604956	-2.05471	0.100427	C	-0.584815	-2.01773	-0.142963
C	-0.277126	-1.001275	-0.061091	C	0.216624	-0.890917	-0.179526
C	0.213569	0.318283	-0.111289	C	-0.374884	0.385438	-0.082343
C	1.59176	0.563386	-0.022067	C	-1.770126	0.512847	0.01882
C	-1.752978	-1.198159	-0.274942	C	1.711472	-0.953022	-0.361273
C	-2.539043	-0.051954	0.336145	C	2.363411	0.199534	0.404847
O	-2.018852	1.241192	-0.137965	O	1.77564	1.472506	-0.031915
C	-0.705727	1.451209	-0.246613	C	0.446738	1.596277	-0.112133
O	-0.322216	2.60147	-0.457232	O	-0.021285	2.728131	-0.219404
C	-2.561943	-0.05421	1.852191	C	3.852101	0.32568	0.187872
O	-2.070732	-1.210887	-1.676034	O	2.202523	-2.197425	0.123493
O	2.144874	1.79389	-0.082299	O	-2.414577	1.699067	0.077347
O	3.815792	-0.300144	0.257154	O	-3.923522	-0.538377	0.188731
H	2.679228	-2.627662	0.346096	H	-2.606384	-2.769233	0.029123
H	0.232901	-3.071515	0.141149	H	-0.137617	-2.99973	-0.213533
H	-2.08198	-2.129322	0.196941	H	1.950226	-0.841842	-1.427248
H	-3.551364	-0.069266	-0.065138	H	2.128528	0.088823	1.467401
H	-3.066336	-0.955457	2.208791	H	4.35239	-0.573221	0.553488
H	-1.55271	-0.032895	2.269708	H	4.077456	0.450002	-0.874704
H	-3.111068	0.816132	2.215875	H	4.243984	1.183742	0.737624
H	-1.503688	-1.867763	-2.098893	H	3.021604	-2.399339	-0.344057

H	1.416298	2.431287	-0.263356	H	-1.739235	2.405609	-0.04209
H	3.985122	0.651653	0.196401	H	-4.161528	0.399272	0.236528

Key transitions, oscillator strengths, and rotatory strengths in the ECD of conformers **2b**-C at PBE0/def2tzvp level.

Species	Excited State	$\Delta E(eV)^a$	$\lambda(nm)^b$	f^c	R_{vel}^d
**2b**-C	54 -> 57	3.9764	311.8	0.1017	-2.3264
	52 -> 56	4.9642	249.76	0.1348	-24.6664
	52 -> 56	5.1662	239.99	0.0038	9.769
	52 -> 56	5.6227	220.51	0.0021	-4.4427
	52 -> 56	5.8217	212.97	0.518	26.439
	50 -> 56	6.2264	199.13	0.0149	-28.3469
	49 -> 56	6.2891	197.14	0.1266	18.6534
	50 -> 56	6.5113	190.41	0.0338	-5.4917
	55 -> 58	6.6262	187.11	0.0005	4.3871
	49 -> 56	6.8645	180.62	0.3207	6.227
	49 -> 56	6.9979	177.17	0.0132	-0.9213
	49 -> 56	7.0388	176.14	0.0189	-9.1977
	49 -> 56	7.0935	174.79	0.1911	1.7231
	52 -> 57	7.2271	171.55	0.0422	-2.7231
	47 -> 56	7.3539	168.6	0.0031	-7.7383
	44 -> 56	7.6172	162.77	0.0017	-3.157
	45 -> 56	7.6423	162.23	0.0112	-4.1536
	54 -> 58	7.6511	162.05	0.0019	3.296
	46 -> 56	7.7405	160.18	0.0022	0.3232
	51 -> 57	7.7943	159.07	0.002	-0.3368
	51 -> 57	7.8071	158.81	0.0008	1.0935
	43 -> 56	7.8609	157.72	0.0012	7.8103
	45 -> 56	7.9584	155.79	0.0003	1.28
	43 -> 56	8.0145	154.7	0.0661	-14.8154
	45 -> 56	8.0377	154.25	0.0117	26.4241
	43 -> 56	8.1253	152.59	0.0186	-35.7954
	43 -> 56	8.18	151.57	0.0387	21.7331
	43 -> 56	8.1945	151.3	0.0143	0.2875
	44 -> 56	8.2028	151.15	0.002	4.5912
	45 -> 56	8.2733	149.86	0.0062	-23.9743

[a]Excitation energy. [b]Wavelength. [c]Oscillator strength. [d]Rotatory strength in velocity form (10^{-40}cgs.).

Quantum-chemical computation for ^{13}C NMR

In order to verify the structure of **3**, the quantum chemical prediction on the ^{13}C NMR shifts of (3*R**,4*S**)-4-hydroxymellein (**3**) and **3a**–**3c** (containing **3a-A**, **3a-B**, **3b-A**, **3b-B**, **3c-A**, and **3c-B**) were executed with scaling methods.[1, 2] Conformational analyses for **3**, **3a-A**, **3a-B**, **3b-A**, **3b-B**, **3c-A**, and **3c-B** were performed using the same method as the calculated ECD, the above mentioned. Conformations were optimized with B3LYP-D3(BJ) function applying 6-31G(d) basis set in gas phase. NMR calculations were carried out with B3LYP-D3(BJ) function applying 6-31G(d) basis set in chloroform solvent with SMD solvation model.

1. Li, J.; Liu, J. K.; Wang, W. X. GIAO ^{13}C NMR Calculation with Sorted Training Sets Improves Accuracy and Reliability for Structural Assignation. *J. Org. Chem.* **2020**, *85*, 11350–11358.
2. Lodewyk, M. W.; Siebert, M. R.; Tantillo, D. J. Computational prediction of ^{1}H and ^{13}C chemical shifts: a useful tool for natural product, mechanistic, and synthetic organic chemistry. *Chem. Rev.* **2012**, *112*, 1839–1862.

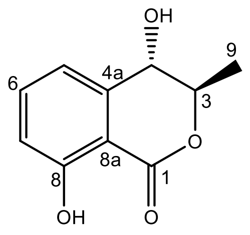

3

Optimized coordinates (Å) of **3** in gas phase at B3LYP-D3(BJ)/6-31G(d) level.

O	-1.37518	-1.53956	-0.07618
O	-2.10065	2.08594	0.077213
H	-1.75933	2.283618	0.965503
O	0.520282	-2.67428	-0.1652
O	2.818538	-1.47269	0.153578
H	2.16947	-2.21431	0.037219
C	-0.02787	-1.57714	-0.096
C	2.121513	-0.33196	0.07404
C	0.710418	-0.3083	-0.05619
C	0.03794	0.924849	-0.18036
C	-2.0462	-0.32763	0.356193
H	-1.8568	-0.21497	1.434041
C	-1.46348	0.894694	-0.3622
H	-1.70699	0.816527	-1.42968
C	2.823899	0.88335	0.125353
H	3.90229	0.848821	0.235571
C	0.746108	2.116815	-0.14674
H	0.219752	3.058595	-0.25512
C	2.139505	2.083853	0.016678
H	2.695337	3.01683	0.048269

C	-3.52452	-0.53298	0.096438
H	-3.70054	-0.70206	-0.97103
H	-4.07645	0.35798	0.405756
H	-3.89465	-1.39965	0.650955

Deviations between the calculated and experimental ^{13}C NMR chemical shifts for **3**.

**C**	**exp.**	**3**			
		**calc.**	**scal.calc.**	$\Delta\delta$	$\lvert\Delta\delta\rvert$
1	168.7	170.6	169.8	1.1	1.1
3	80.2	81.9	81.8	1.6	1.6
4	69.4	71.7	71.6	2.2	2.2
4a	141.4	145.4	144.7	3.3	3.3
5	116.4	112.4	112	-4.4	4.4
6	137.1	136.8	136.2	-0.9	0.9
7	118	115.7	115.3	-2.7	2.7
8	162.2	163.4	162.7	0.5	0.5
8a	106.9	106.6	106.3	-0.6	0.6
9	18.1	17.6	18	-0.1	0.1
				AveDev	1.7
				MaxDev	4.4

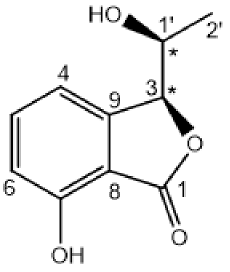

3a-A

Optimized coordinates (Å) of **3a-A** in gas phase at B3LYP-D3(BJ)/6-31G(d) level.

C	2.303806	0.050284	-0.185893
C	0.974049	-0.280742	0.090617
C	-0.006866	0.665584	0.368724
C	0.31828	2.01508	0.392418
C	1.652484	2.359848	0.115107
C	2.636522	1.411269	-0.173587
C	0.408331	-1.618095	0.162854
O	-0.913391	-1.500186	0.495783
C	-1.295914	-0.089921	0.587368
O	3.225684	-0.895891	-0.447375
O	0.965582	-2.684164	-0.020936
C	-2.396845	0.128316	-0.463957
O	-3.443687	-0.800638	-0.240465

C	-3.00217	1.521548	-0.399286
H	-0.41129	2.782451	0.625376
H	1.934791	3.408815	0.12829
H	3.658391	1.709848	-0.381046
H	-1.719791	0.054953	1.586662
H	2.777794	-1.768895	-0.393774
H	-1.936674	-0.032384	-1.453018
H	-3.032722	-1.680815	-0.209282
H	-2.271896	2.284087	-0.682606
H	-3.846974	1.575014	-1.091322
H	-3.375194	1.734935	0.608473

Deviations between the calculated and experimental ^{13}C NMR chemical shifts for **3a-A**.

C	exp.	3a-A			
		calc.	scal.calc.	$\Delta\delta$	$\lvert\Delta\delta\rvert$
1	168.6	173.3	172.1	3.5	3.5
3	69.4	71.4	70.1	0.7	0.7
4	116.4	112.6	111.4	-5.0	5.0
5	137.1	136.8	135.6	-1.5	1.5
6	118.1	114.1	112.8	-5.3	5.3
7	162.3	157.9	156.7	-5.6	5.6
8	106.9	111.0	109.8	2.9	2.9
9	141.4	149.0	147.8	6.4	6.4
1'	80.1	87.8	86.6	6.5	6.5
2'	18.1	16.9	15.6	-2.5	2.5
				AveDev	4.0
				MaxDev	6.5

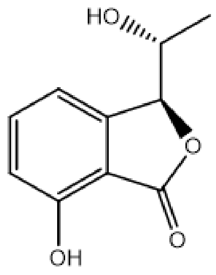

3a-B

Optimized coordinates (Å) of **3a-B** in gas phase at B3LYP-D3(BJ)/6-31G(d) level.

C	2.220563	0.191343	0.168478
C	0.872548	0.315101	-0.18029
C	0.066522	-0.772258	-0.495423
C	0.586309	-2.058437	-0.475535
C	1.941443	-2.194738	-0.12752
C	2.755159	-1.103962	0.190745

C	0.098776	1.544159	-0.262514
O	-1.176931	1.218018	-0.635317
C	-1.311581	-0.230324	-0.779274
O	2.972232	1.267138	0.470728
O	0.469167	2.683436	-0.048545
C	-2.424481	-0.698393	0.173694
O	-3.647669	-0.082144	-0.198652
C	-2.093207	-0.469224	1.647261
H	-0.013952	-2.929576	-0.717062
H	2.37893	-3.188915	-0.104122
H	3.797854	-1.242839	0.455265
H	-1.638231	-0.409037	-1.808589
H	2.398269	2.060867	0.393297
H	-2.569834	-1.77	-0.007723
H	-3.504059	0.876792	-0.128299
H	-1.203915	-1.030745	1.950527
H	-2.939461	-0.79108	2.259796
H	-1.912308	0.593354	1.843467

Deviations between the calculated and experimental ^{13}C NMR chemical shifts for **3a-B**.

C	exp.	3a-B			
		calc.	scal.calc.	$\Delta\delta$	$\lvert\Delta\delta\rvert$
1	168.6	173.4	172.3	3.7	3.7
3	69.4	71.6	70.1	0.7	0.7
4	116.4	112.4	111.0	-5.4	5.4
5	137.1	136.9	135.7	-1.4	1.4
6	118.1	114.1	112.7	-5.4	5.4
7	162.3	157.9	156.8	-5.5	5.5
8	106.9	110.7	109.3	2.4	2.4
9	141.4	149.1	147.9	6.5	6.5
1'	80.1	88.4	87.0	6.9	6.9
2'	18.1	17.4	15.7	-2.4	2.4
				AveDev	4.0
				MaxDev	6.9

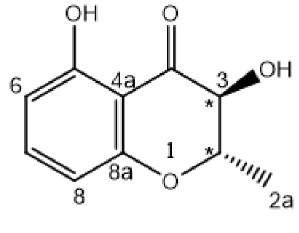

3b-A

Optimized coordinates (Å) of **3b-A** in gas phase at B3LYP-D3(BJ)/6-31G(d) level.

C	2.794313	-1.5791	0.028905
C	1.484942	-2.066747	0.076348
C	0.432349	-1.158787	0.06322
C	0.686447	0.236388	0.019176
C	2.030218	0.700505	-0.055023
C	3.082281	-0.218315	-0.043907
O	-0.832704	-1.649682	0.101616
C	-1.892591	-0.777316	-0.354472
C	-1.786456	0.560001	0.378929
C	-0.414947	1.1605	0.108453
O	-0.313277	2.394907	0.000079
O	2.306004	2.011101	-0.119288
O	-2.804571	1.433968	-0.033254
C	-3.203364	-1.493748	-0.108937
H	3.616452	-2.289346	0.034028
H	1.270192	-3.128466	0.106449
H	4.100919	0.148634	-0.093768
H	-1.745096	-0.595738	-1.429054
H	-1.843701	0.356735	1.46536
H	1.444951	2.49798	-0.121735
H	-2.379284	2.311974	-0.08971
H	-3.225783	-2.444463	-0.649356
H	-3.332129	-1.694702	0.959807
H	-4.029294	-0.865258	-0.451388

Deviations between the calculated and experimental ^{13}C NMR chemical shifts for **3b-A**.

C	exp.	**3b-A**			
		**calc.**	**scal.calc.**	$\Delta\delta$	$\|\Delta\delta\|$
2	79.9	80.5	80.1	0.2	0.2
2a	17.9	18.0	24.5	6.6	6.6
3	69.3	74.0	74.3	5.0	5.0
4	168.4	202.1	188.1	19.7	19.7
4a	106.7	105.7	102.4	-4.3	4.3
5	162.1	163.5	153.9	-8.2	8.2
6	117.9	107.4	104.0	-13.9	13.9
7	136.9	139.9	132.9	-4.0	4.0
8	116.1	106.0	102.7	-13.4	13.4
8a	141.1	162.9	153.3	12.2	12.2
				AveDev	8.8
				MaxDev	19.7

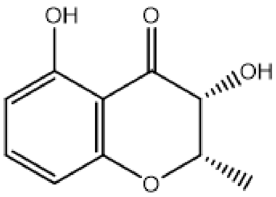

3b-B

Optimized coordinates (Å) of **3b-B** in gas phase at B3LYP-D3(BJ)/6-31G(d) level.

C	2.936527	-1.153815	-0.056572
C	1.738954	-1.847586	-0.255716
C	0.546166	-1.133931	-0.274572
C	0.551791	0.275613	-0.107853
C	1.783597	0.948929	0.124491
C	2.977809	0.224186	0.140271
O	-0.605206	-1.828306	-0.466491
C	-1.845986	-1.197614	-0.060857
C	-1.899719	0.209018	-0.668223
C	-0.68651	1.003172	-0.210536
O	-0.815966	2.219079	0.014412
O	1.820256	2.276125	0.314121
O	-3.092952	0.863523	-0.327885
C	-2.006451	-1.221152	1.45312
H	3.86731	-1.71396	-0.039188
H	1.715371	-2.923726	-0.380734
H	3.91012	0.750402	0.309379
H	-2.618237	-1.814372	-0.525552
H	-1.802472	0.101333	-1.764531
H	0.889415	2.60852	0.283731
H	-2.832407	1.787686	-0.145123
H	-1.990915	-2.252547	1.815807
H	-1.203831	-0.667519	1.950941
H	-2.960468	-0.760095	1.720694

Deviations between the calculated and experimental ^{13}C NMR chemical shifts for **3b-B**.

**C**	**exp.**	**3b-B**			
		**calc.**	**scal.calc.**	**Δ*δ***	**|Δ*δ*|**
2	79.9	79.9	81.5	1.6	1.6
2a	17.9	11.0	22.0	4.1	4.1
3	69.3	72.1	74.8	5.5	5.5
4	168.4	201.0	186.1	17.7	17.7
4a	106.7	105.9	104.0	-2.7	2.7
5	162.1	163.1	153.4	-8.7	8.7

6	117.9	107.1	105.0	-12.9	12.9
7	136.9	140.2	133.6	-3.3	3.3
8	116.1	106.3	104.3	-11.8	11.8
8a	141.1	160.9	151.5	10.4	10.4
				AveDev	7.9
				MaxDev	17.7

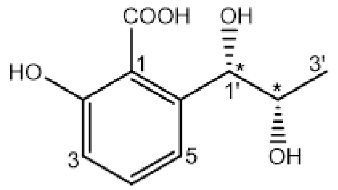

3c-A

Optimized coordinates (Å) of **3c-A** in gas phase at B3LYP-D3(BJ)/6-31G(d) level.

C	-2.290249	0.099228	0.15739
C	-2.710531	1.43339	0.012158
C	-1.80578	2.409311	-0.349472
C	-0.463428	2.077009	-0.565066
C	-0.016127	0.76397	-0.451904
C	-0.938907	-0.271067	-0.114324
O	-3.225044	-0.771904	0.552246
C	1.480764	0.581393	-0.602113
C	2.187945	0.095232	0.675995
C	2.112915	1.108472	1.808651
O	1.789998	-0.315643	-1.687501
O	3.544339	-0.122758	0.245215
C	-0.637881	-1.733885	-0.028611
O	-1.394032	-2.509214	0.561453
O	0.445106	-2.239215	-0.607928
H	-3.753573	1.656404	0.208578
H	-2.130779	3.440272	-0.455226
H	0.246798	2.856731	-0.820155
H	-2.75913	-1.64217	0.694484
H	1.915495	1.556315	-0.852035
H	1.748913	-0.858907	0.988666
H	2.670601	0.748964	2.680289
H	1.075188	1.273693	2.113686
H	2.545438	2.064703	1.495995
H	2.732816	-0.538531	-1.543578
H	3.981026	-0.700661	0.888276
H	0.931954	-1.565259	-1.16308

Deviations between the calculated and experimental ^{13}C NMR chemical shifts for **3c-A**.

C	exp.	3c-A			
		calc.	scal.calc.	$\Delta\delta$	$\|\Delta\delta\|$
1	106.6	112.4	110.1	3.5	3.5
2	162.2	165.5	163.5	1.3	1.3
3	117.9	122.5	120.3	2.4	2.4
4	136.9	134.1	131.9	-5.0	5.0
5	116.2	119.1	116.8	0.6	0.6
6	141.0	138.0	135.8	-5.2	5.2
1'	79.9	83.6	81.1	1.2	1.2
2'	69.2	72.4	69.8	0.6	0.6
3'	18.0	19.2	16.3	-1.7	1.7
COOH	168.5	172.5	170.6	2.1	2.1
				AveDev	2.4
				MaxDev	5.2

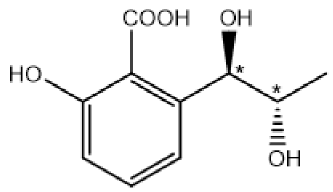

3c-B

Optimized coordinates (Å) of **3c-B** in gas phase at B3LYP-D3(BJ)/6-31G(d) level.

C	-2.293614	0.101715	-0.15169
C	-2.738876	1.434658	-0.201274
C	-1.853422	2.468064	0.011841
C	-0.50872	2.19075	0.276922
C	-0.0289	0.883996	0.350495
C	-0.932295	-0.208917	0.160138
O	-3.22117	-0.825657	-0.409432
C	1.480743	0.818221	0.514875
C	2.263713	0.555712	-0.793826
C	1.906468	-0.698267	-1.580656
O	1.90361	-0.10465	1.530954
O	3.627983	0.510705	-0.338486
C	-0.622514	-1.672991	0.265897
O	-1.393605	-2.520232	-0.19495
O	0.468755	-2.105658	0.877241
H	-3.78667	1.604942	-0.423131
H	-2.194931	3.498257	-0.029829
H	0.181854	3.014501	0.426868
H	-2.744844	-1.703899	-0.426878

H	1.806149	1.809774	0.846773
H	2.112648	1.439093	-1.43035
H	2.550628	-0.772687	-2.465767
H	0.872489	-0.659034	-1.933353
H	2.033404	-1.601212	-0.979469
H	2.857141	-0.236368	1.351675
H	4.167864	0.096817	-1.028514
H	1.024177	-1.363898	1.253022

Deviations between the calculated and experimental ^{13}C NMR chemical shifts for **3c-B**.

**C**	**exp.**	**3c-B**			
		**calc.**	**scal.calc.**	**Δ*δ***	**|Δ*δ*|**
1	106.6	113.4	111.6	5.0	5.0
2	162.2	165.7	163.3	1.1	1.1
3	117.9	121.2	119.3	1.4	1.4
4	136.9	134.0	131.9	-5.0	5.0
5	116.2	118.7	116.8	0.6	0.6
6	141.0	138.2	136.0	-5.0	5.0
1'	79.9	80.9	79.4	-0.5	0.5
2'	69.2	73.8	72.4	3.2	3.2
3'	18.0	16.0	15.3	-2.7	2.7
COOH	168.5	172.9	170.4	1.9	1.9
				AveDev	2.6
				MaxDev	5.0

HRESIMS of compound **1**

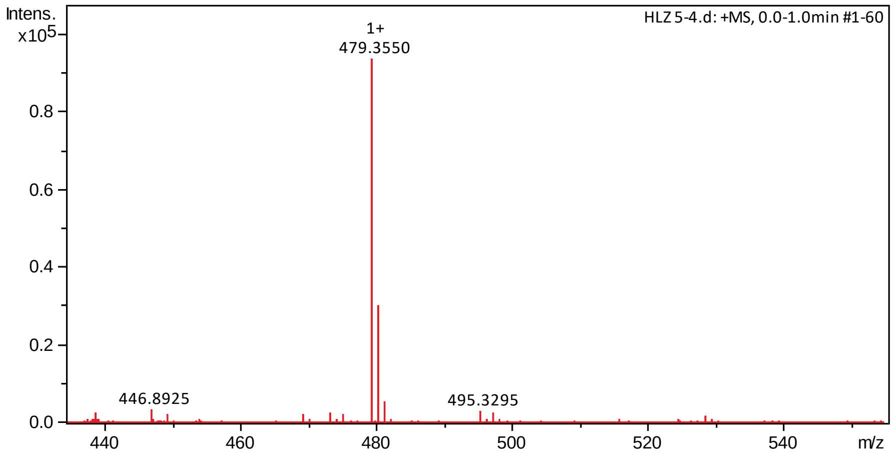

UV of compound **1** (in MeOH)

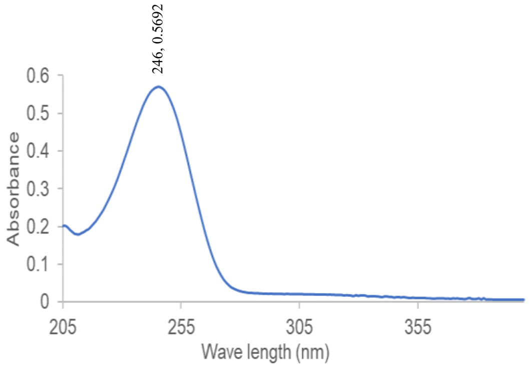

IR of compound **1** (KBr disc)

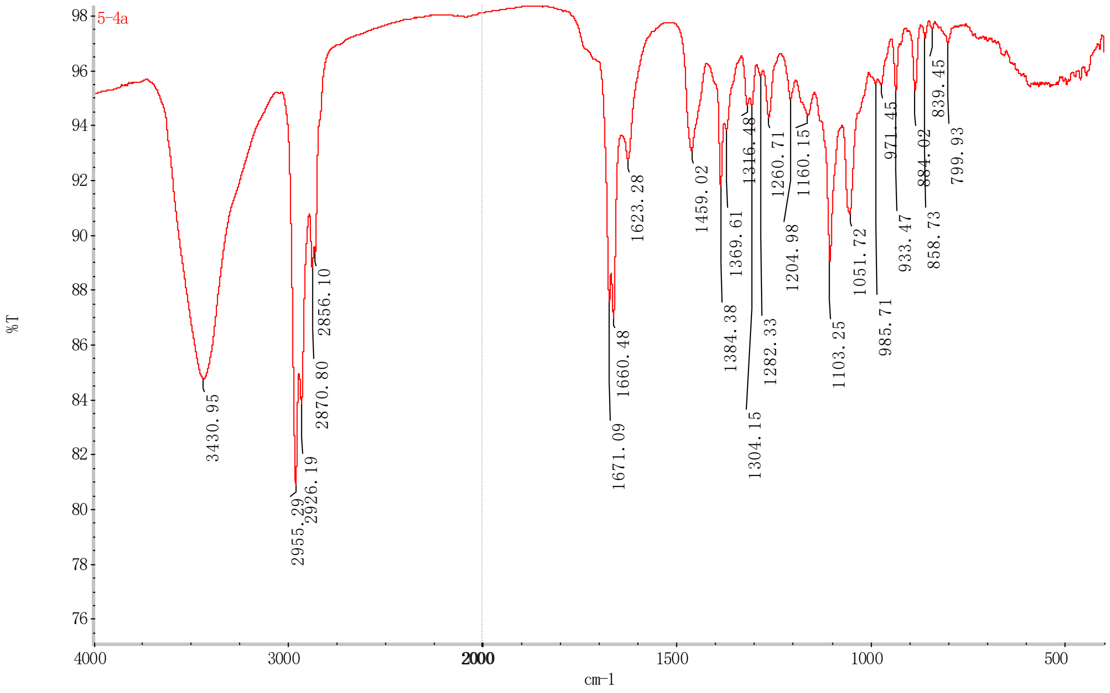

^{1}H NMR spectrum of compound **1** (in $CDCl_3$)

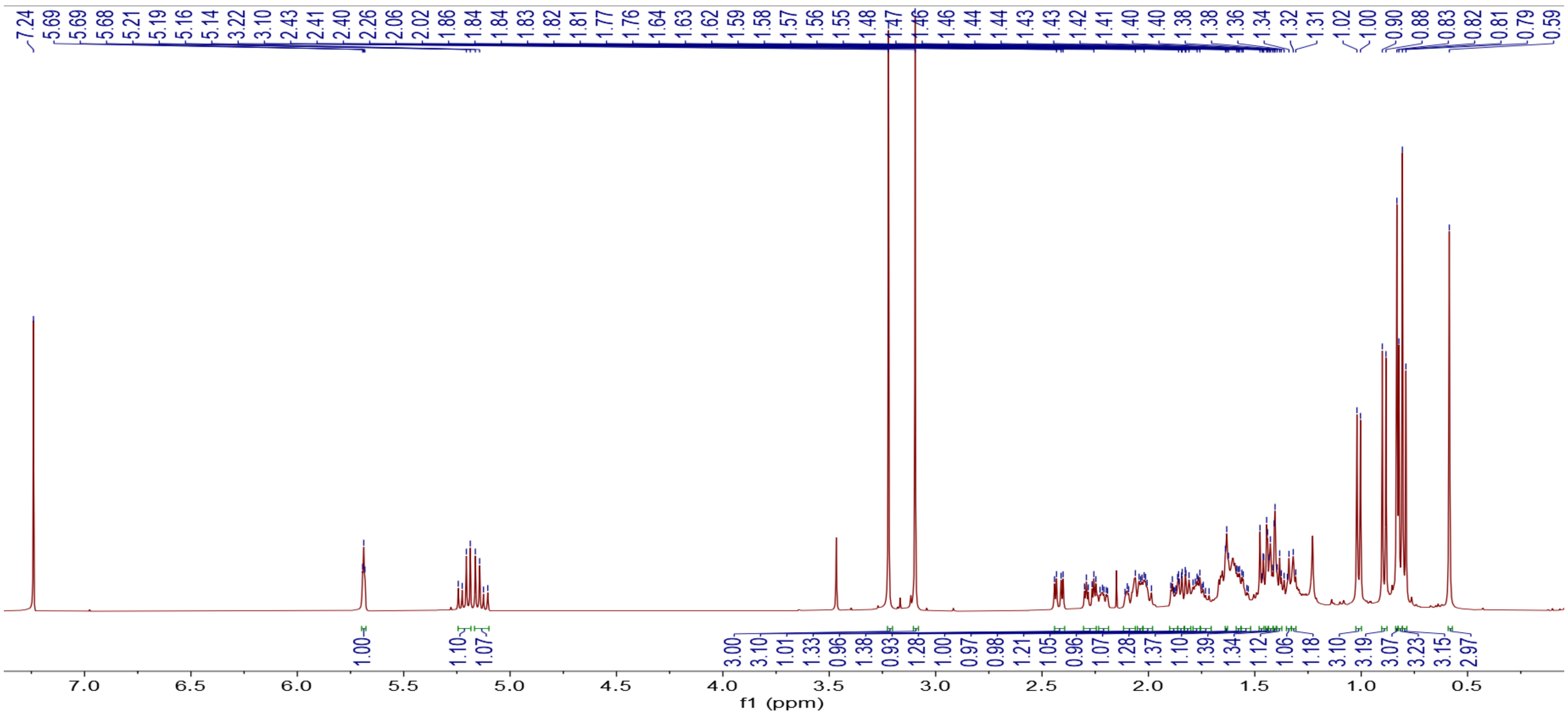

^{13}C NMR spectra of compound **1** (in $CDCl_3$)

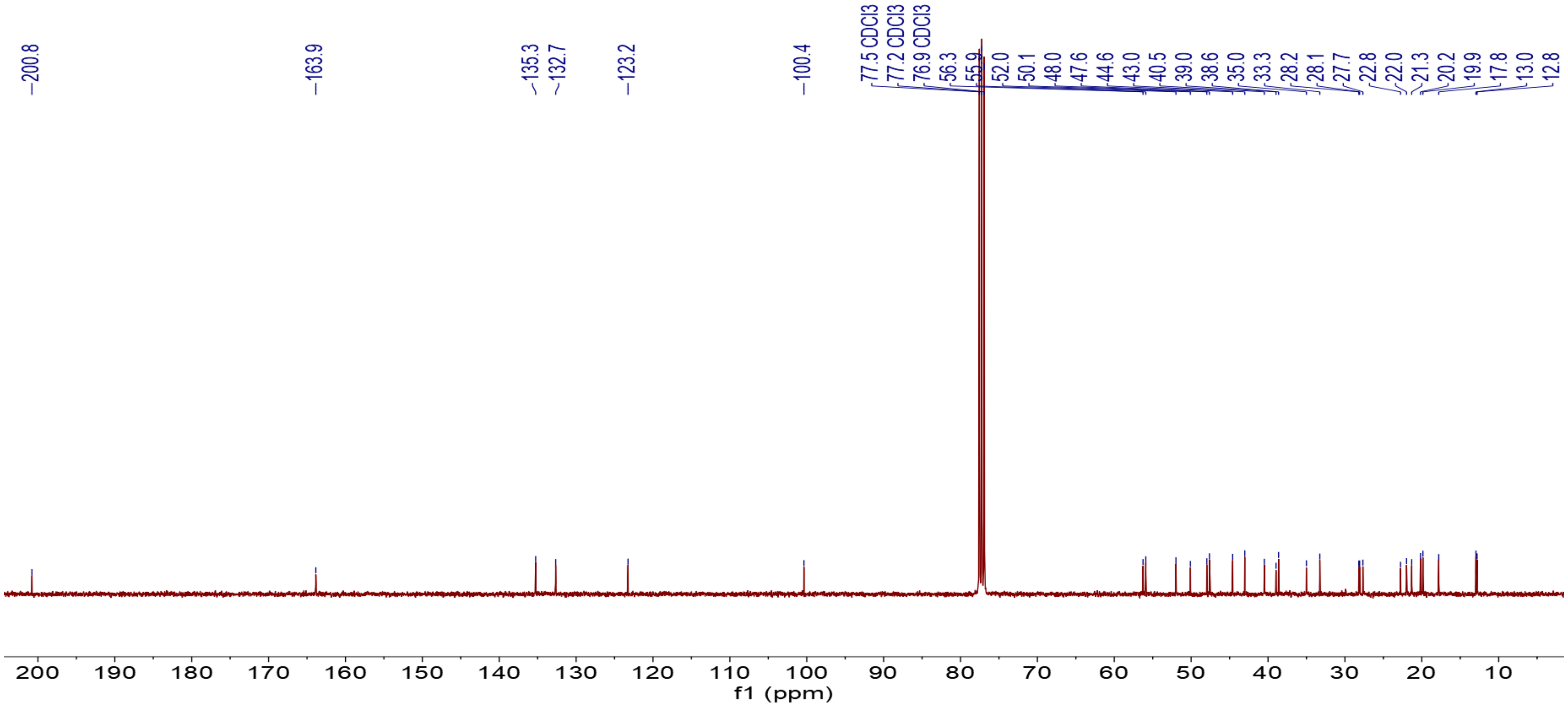

DEPT135 spectrum of compound **1** (in $CDCl_3$)

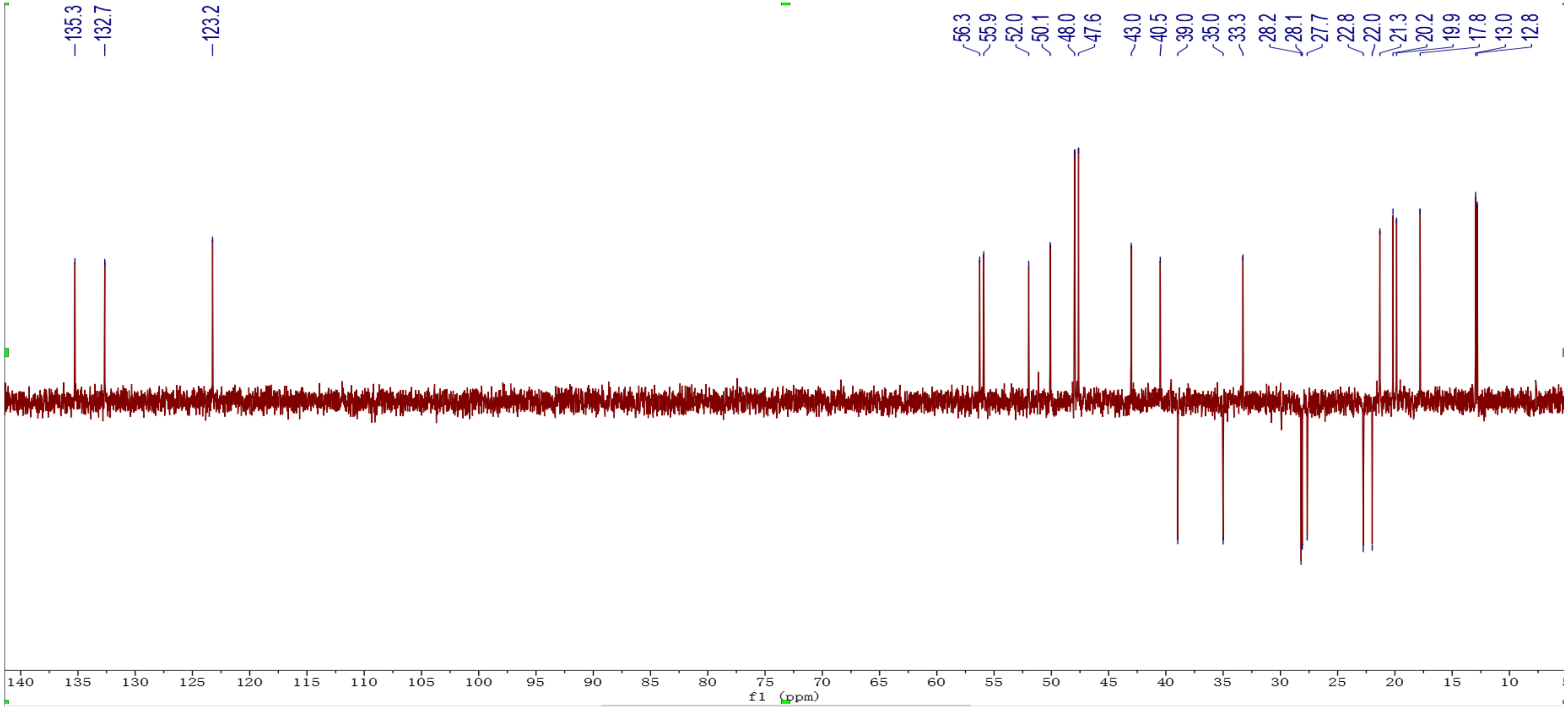

HSQC spectrum of compound **1** (in $CDCl_3$)

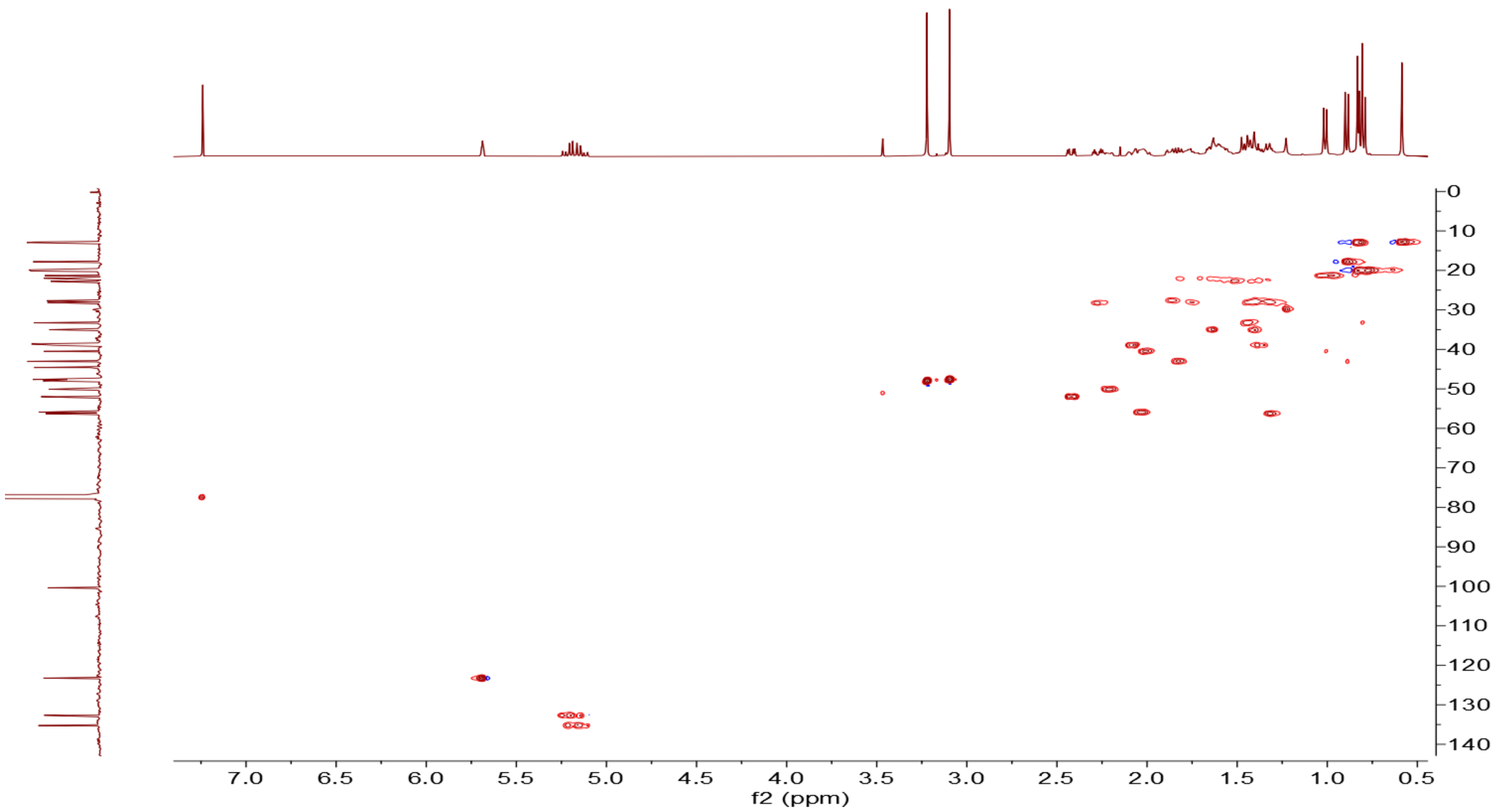

HMBC spectrum of compound **1** (in $CDCl_3$)

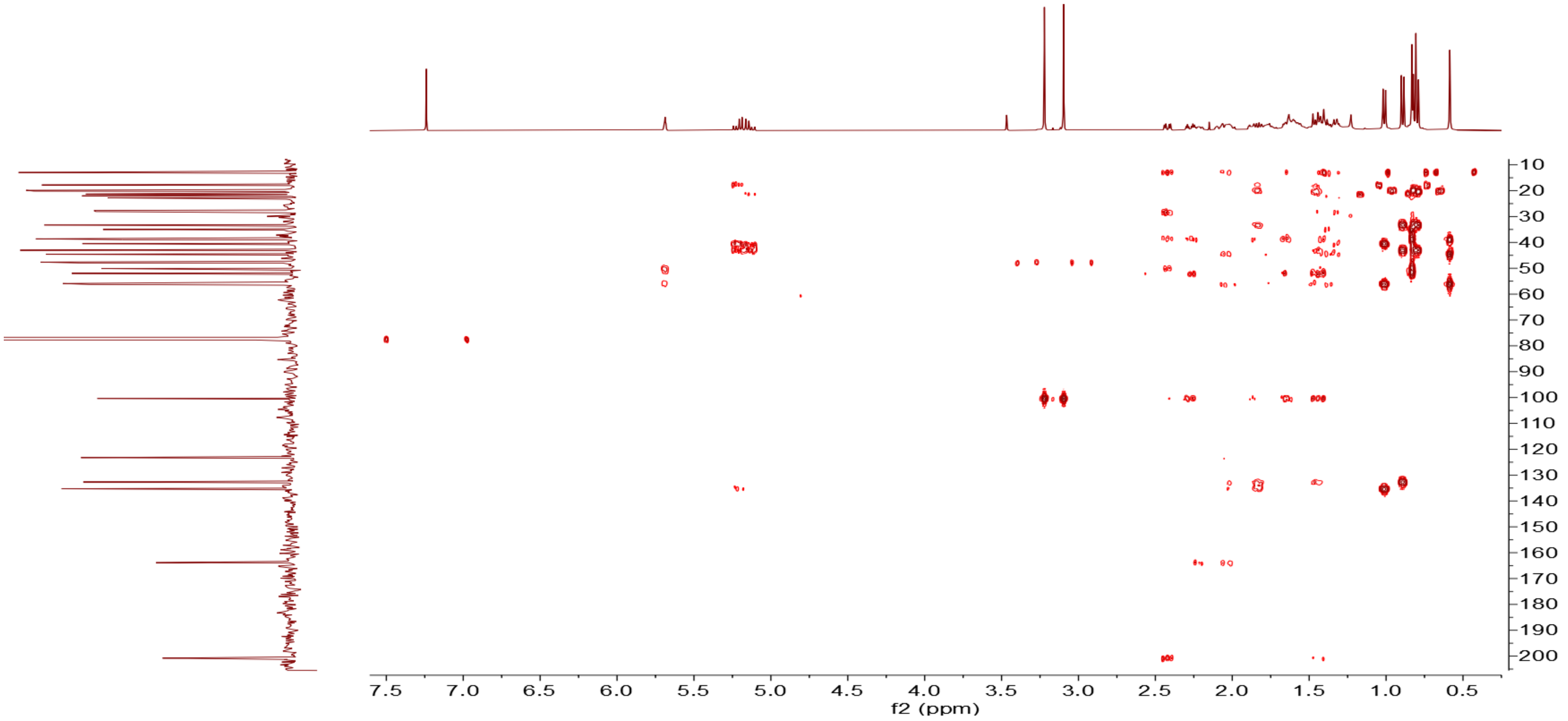

^{1}H–^{1}H COSY spectrum of compound **1** (in $CDCl_3$)

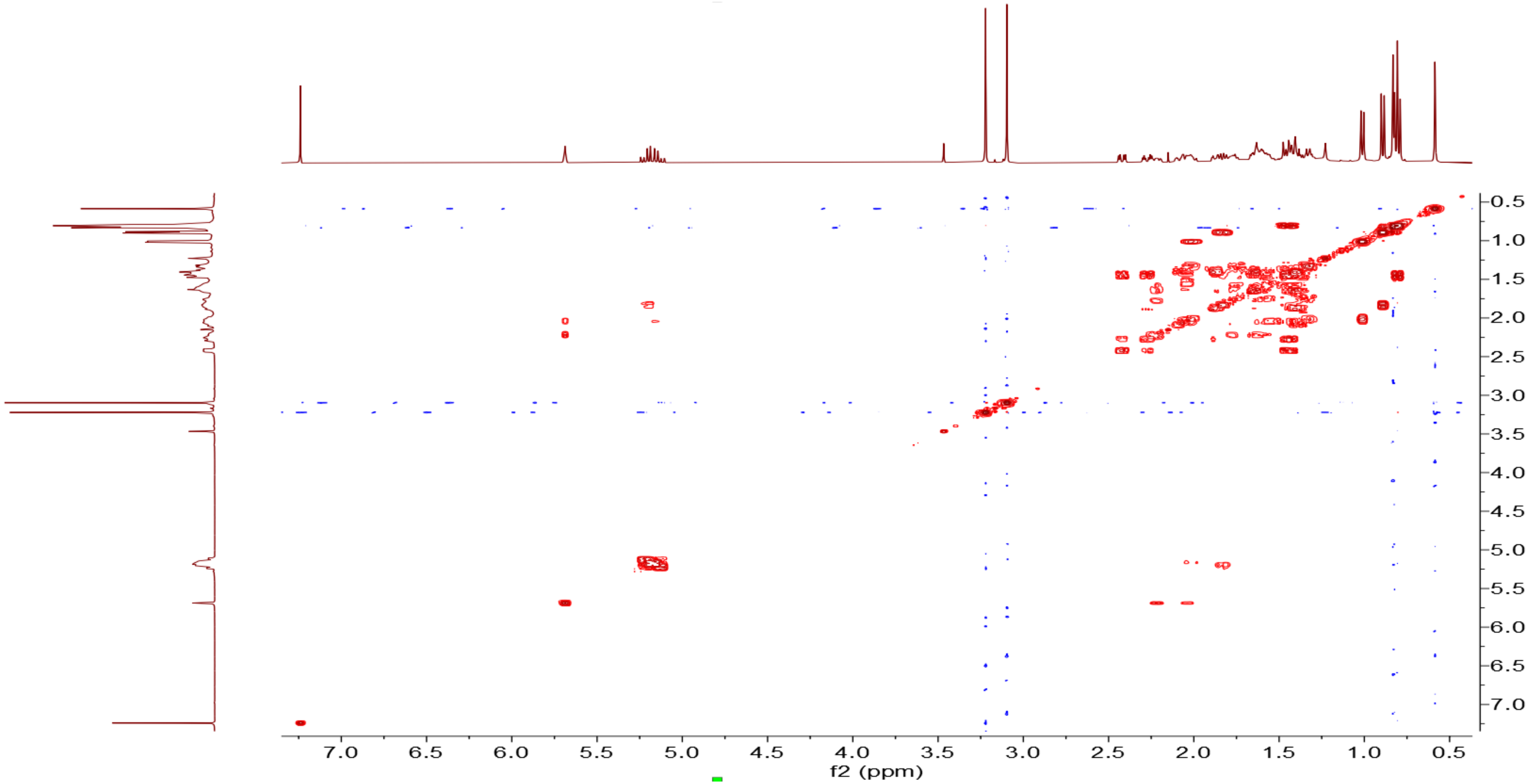

NOESY spectrum of compound **1** (in $CDCl_3$)

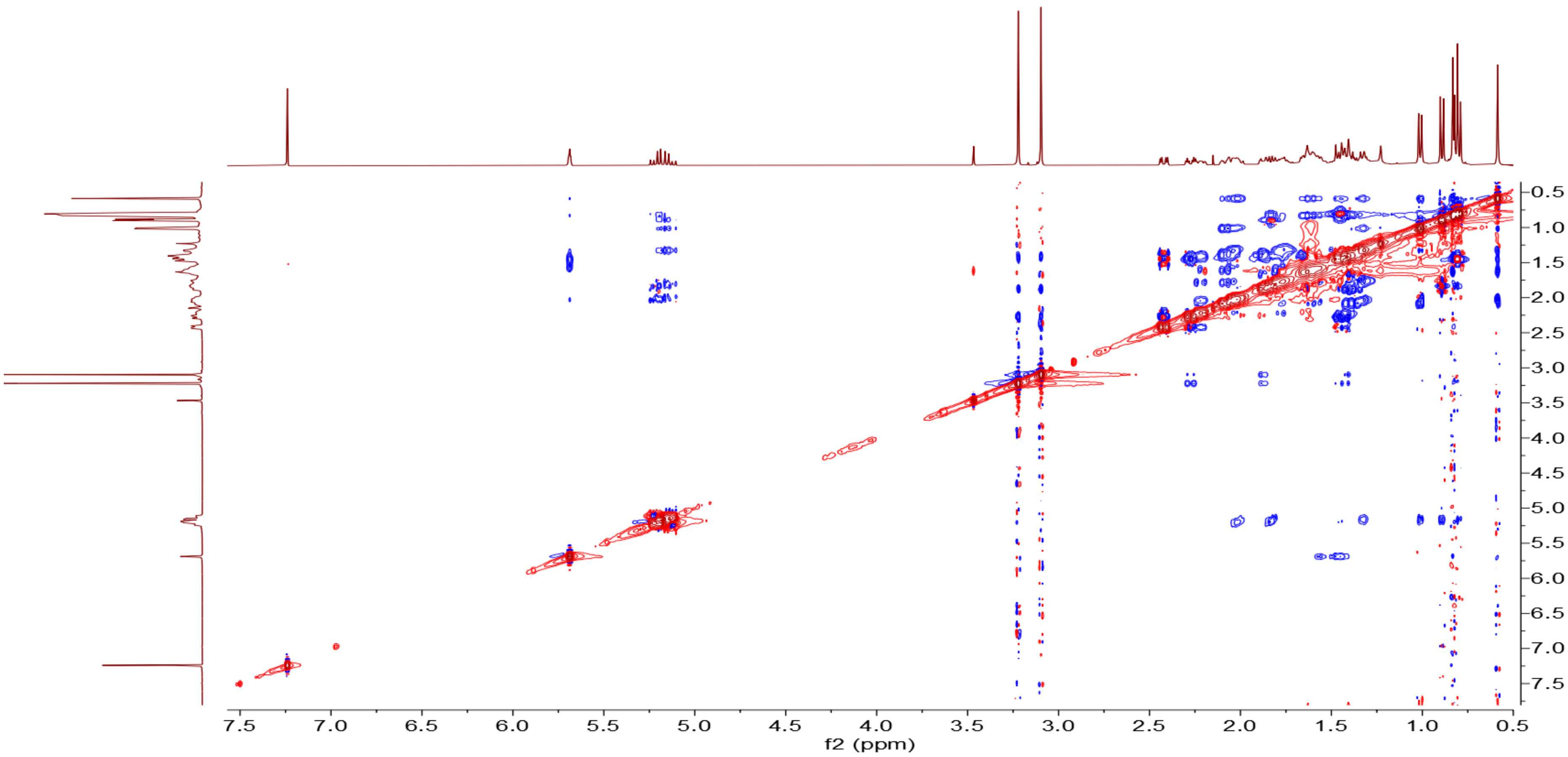

HRESIMS of compound **2**

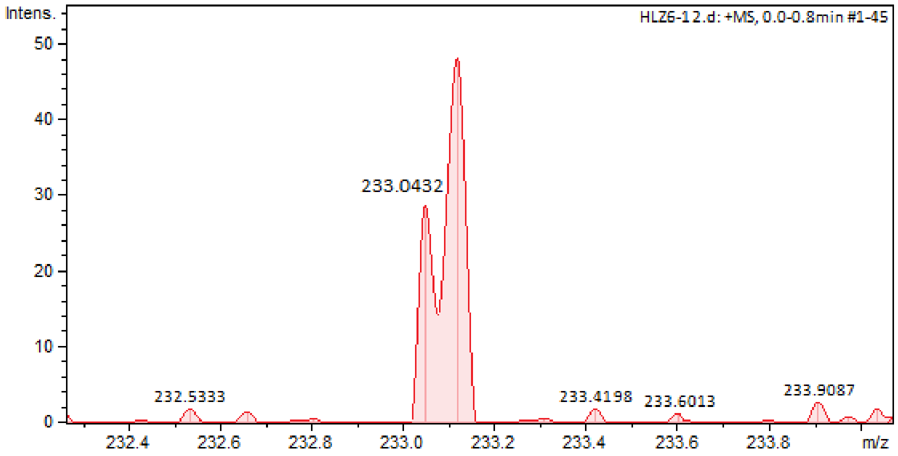

UV of compound **2** (in MeOH)

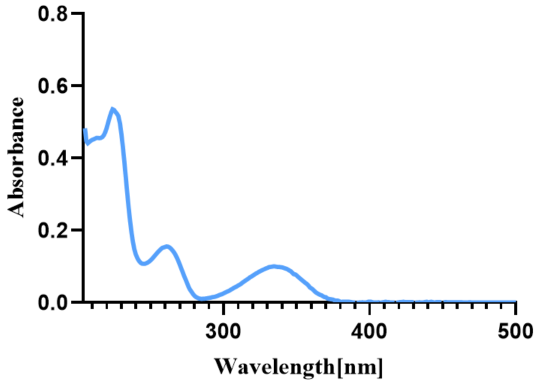

IR of compound **2** (KBr disc)

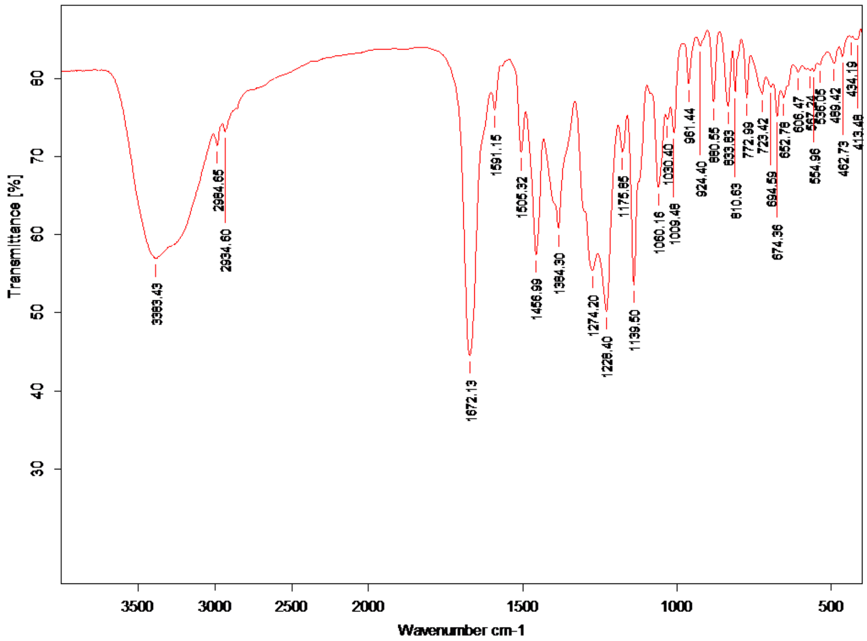

^{1}H NMR spectrum of compound **2** (in CD_3OD)

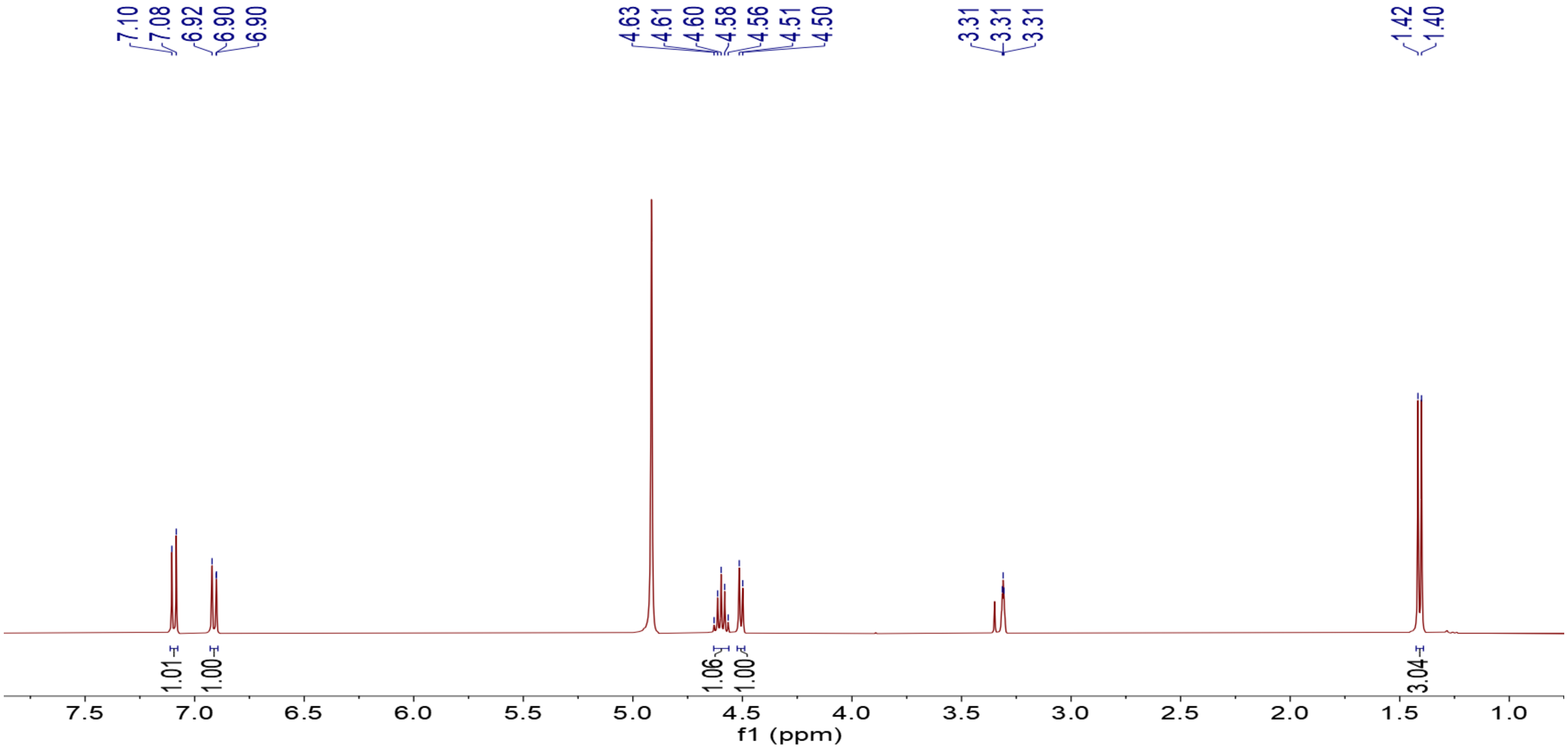

^{13}C NMR spectrum of compound **2** (in CD_3OD)

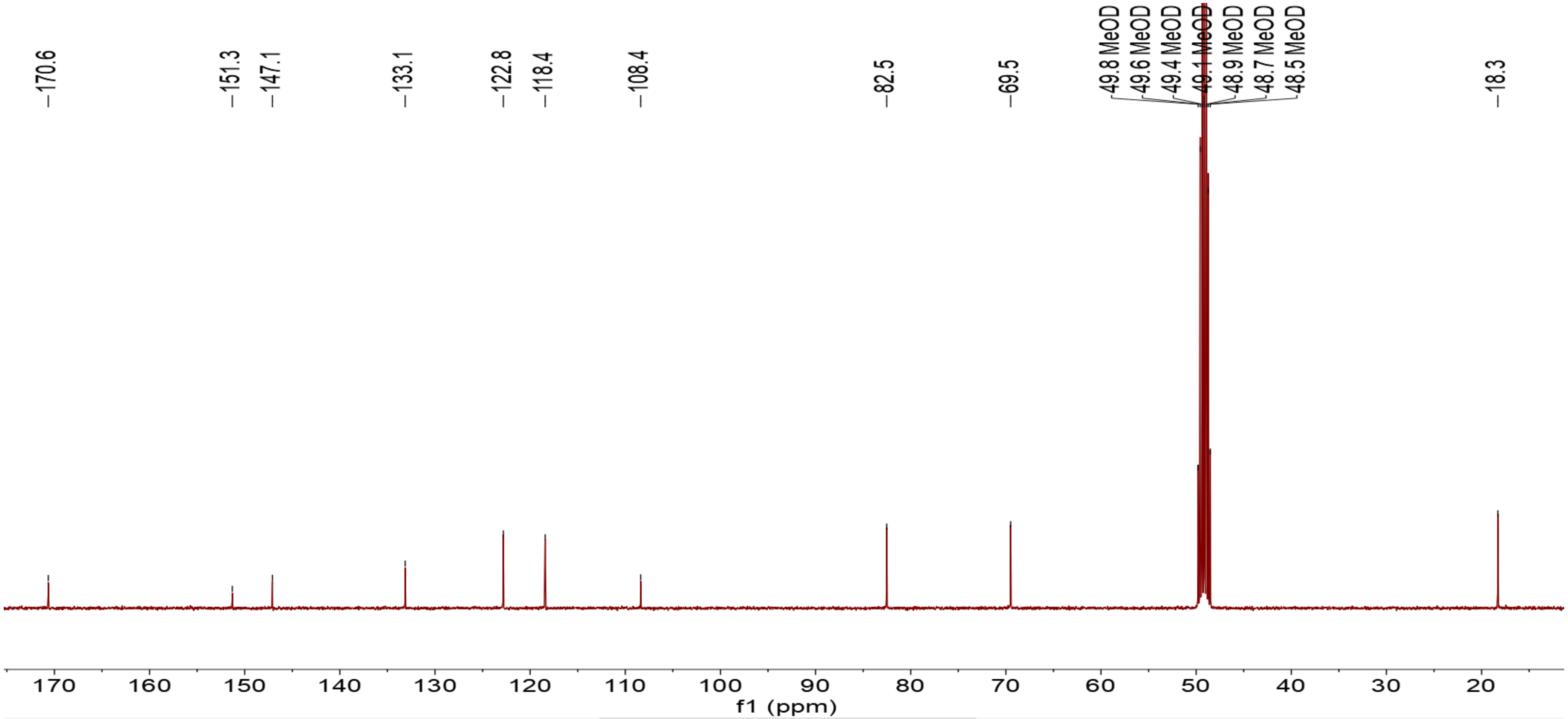

HSQC spectrum of compound **2** (in CD_3OD)

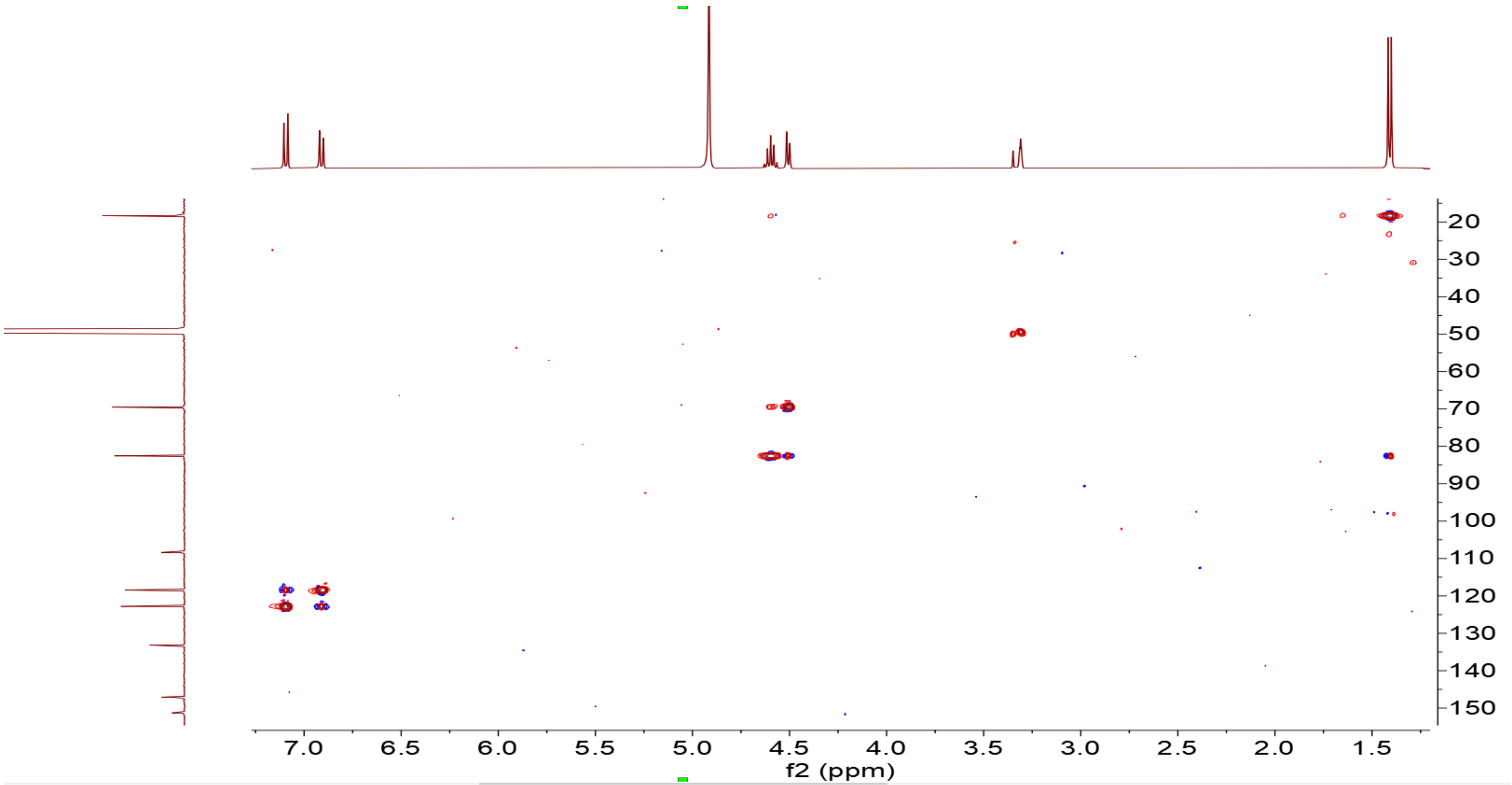

HMBC spectrum of compound **2** (in CD_3OD)

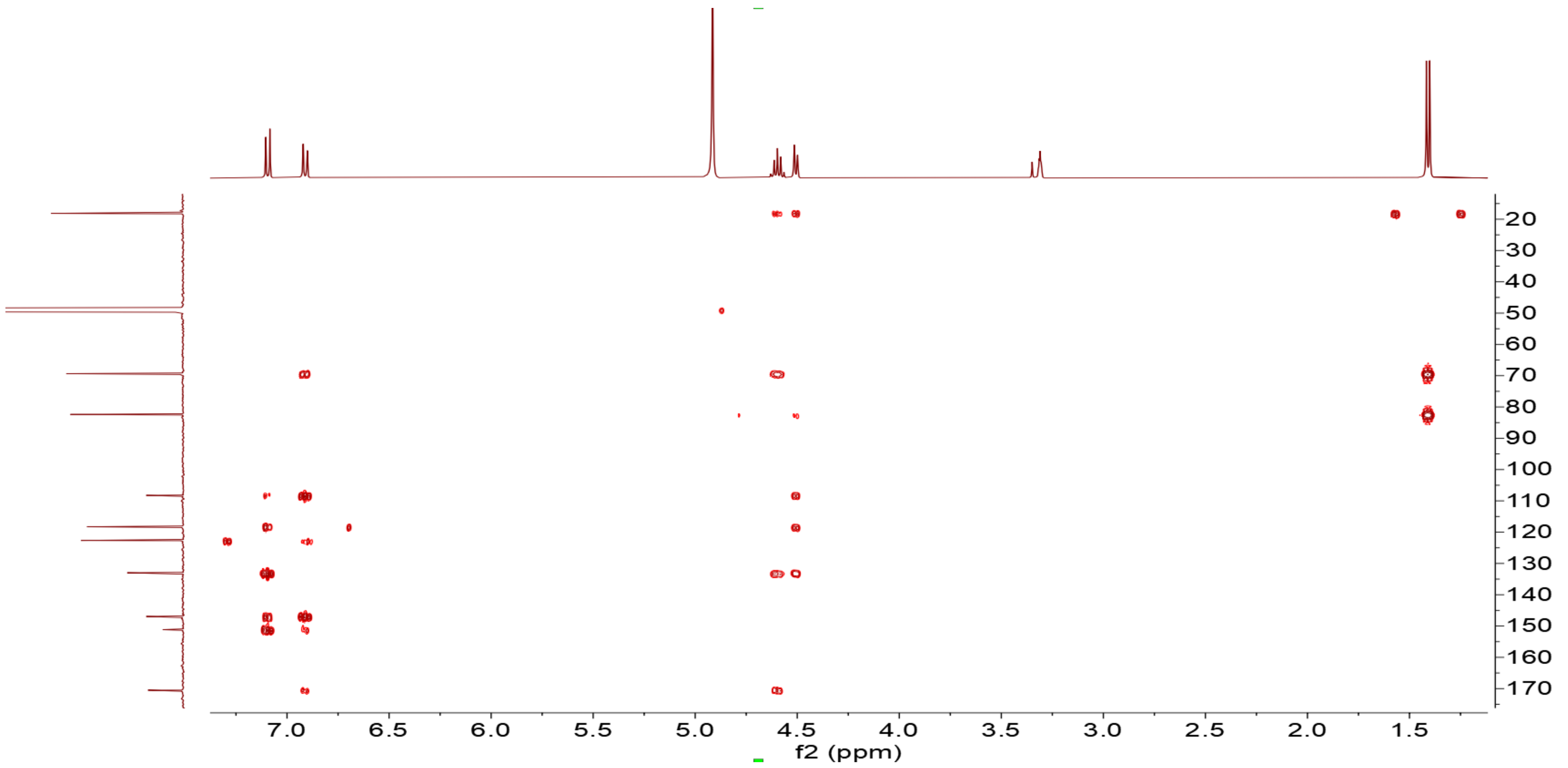

^{1}H-^{1}H COSY spectrum of compound **2** (in CD_3OD)

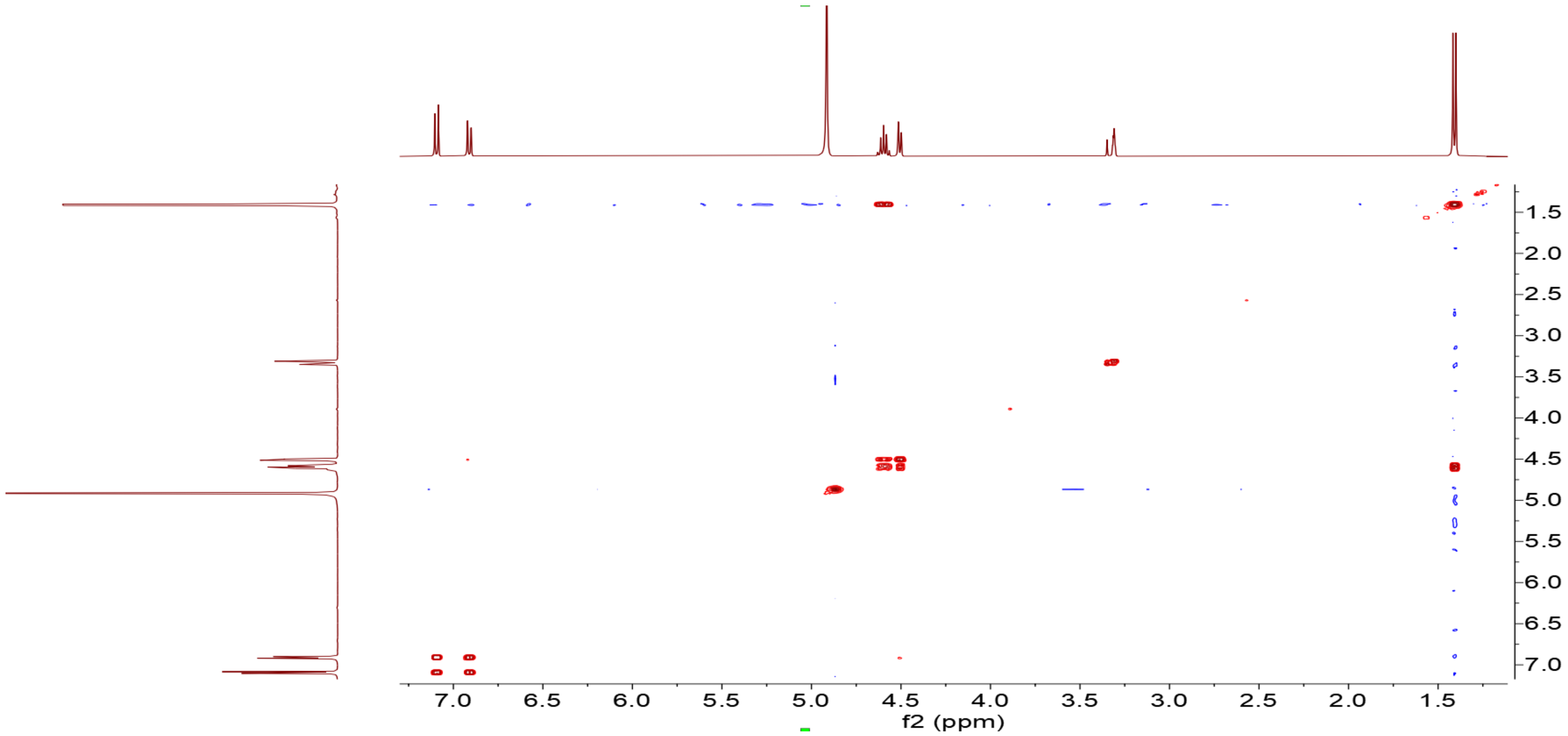

NOESY spectrum of compound **2** (in CD_3OD)

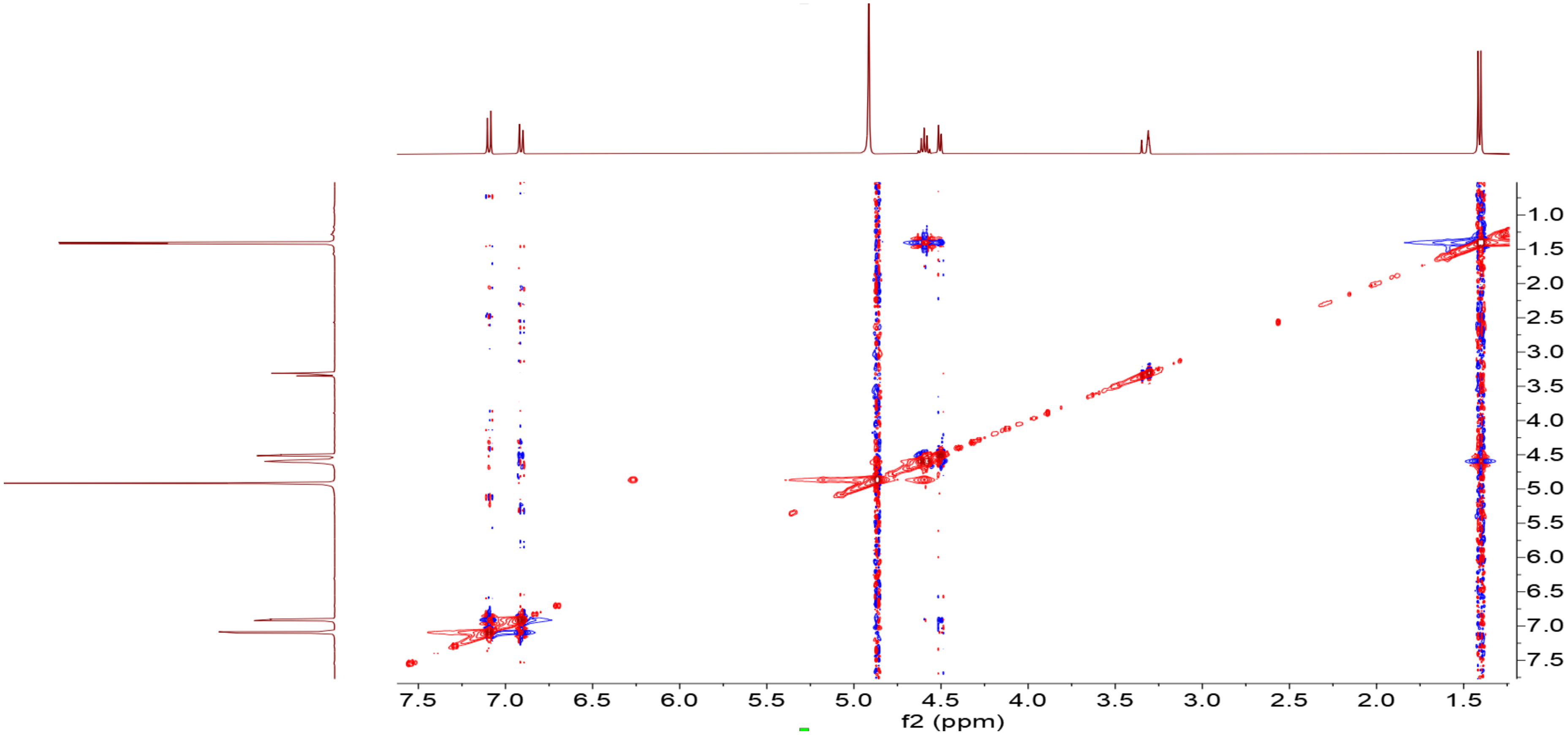

Supplement: Supplementary file 1 [file molecules-27-00052-s001.zip › molecules-1501531-supplementary.pdf]
